# Robust and efficient single-cell Hi-C clustering with approximate k-nearest neighbor graphs

**DOI:** 10.1093/bioinformatics/btab394

**Published:** 2021-05-22

**Authors:** Joachim Wolff, Rolf Backofen, Björn Grüning

**Affiliations:** Bioinformatics Group, Department of Computer Science, University of Freiburg, 79110 Freiburg, Germany; Bioinformatics Group, Department of Computer Science, University of Freiburg, 79110 Freiburg, Germany; Signalling Research Centre CIBSS, University of Freiburg, 79104 Freiburg, Germany; Bioinformatics Group, Department of Computer Science, University of Freiburg, 79110 Freiburg, Germany

## Abstract

**Motivation:**

Hi-C technology provides insights into the 3D organization of the chromatin, and the single-cell Hi-C method enables researchers to gain knowledge about the chromatin state in individual cell levels. Single-cell Hi-C interaction matrices are high dimensional and very sparse. To cluster thousands of single-cell Hi-C interaction matrices, they are flattened and compiled into one matrix. Depending on the resolution, this matrix can have a few million or even billions of features; therefore, computations can be memory intensive. We present a single-cell Hi-C clustering approach using an approximate nearest neighbors method based on locality-sensitive hashing to reduce the dimensions and the computational resources.

**Results:**

The presented method can process a 10 kb single-cell Hi-C dataset with 2600 cells and needs 40 GB of memory, while competitive approaches are not computable even with 1 TB of memory. It can be shown that the differentiation of the cells by their chromatin folding properties and, therefore, the quality of the clustering of single-cell Hi-C data is advantageous compared to competitive algorithms.

**Availability and implementation:**

The presented clustering algorithm is part of the scHiCExplorer, is available on Github https://github.com/joachimwolff/scHiCExplorer, and as a conda package via the bioconda channel. The approximate nearest neighbors implementation is available via https://github.com/joachimwolff/sparse-neighbors-search and as a conda package via the bioconda channel.

**Supplementary information:**

Supplementary data are available at *Bioinformatics* online.

## 1 Introduction

The chromosome conformation capture technique 3C (Dekker *et al.*, 2002) and its successors 4C (Simonis *et al.*, 2006; Zhao *et al.*, 2006), 5C (Dostie *et al.*, 2006) and Hi-C (Lieberman-Aiden *et al.*, 2009) have given insights into the organization of the 3D structure of the DNA and its impact on gene regulation over the last few years. Direct chromatin interactions can provide evidence, for example, for enhancer-promoter interactions and their contribution to the regulation process. Several reviews have been published in recent years, giving a broad overview of different Hi-C techniques and their abilities: Kempfer and Pombo (2020), McCord *et al.* (2020) and Bonev and Cavalli (2016). Single-cell Hi-C (Flyamer *et al.*, 2017; Gassler *et al.*, 2017; Nagano *et al.*, 2013; 2017; Ramani *et al.*, 2017; Stevens *et al.*, 2017) extends Hi-C to individual cells and provides insights into the processes of cell differentiation and division with respect to the dynamics of chromosome conformation. While Hi-C data analysis demands high computational resources, single-cell Hi-C increases this demand further due to the need to not only process one interaction matrix but potentially several thousands of them. Cell clustering, based on the interaction matrices to differentiate by the chromatin folding properties, is one of the most important parts of single-cell Hi-C data analysis to gain information about similarity and, therefore, the linkage between different cells. Hi-C interaction matrices are two-dimensional, representing the contacts between each pair of genomic positions. The interaction matrices do not represent a per base-pair interaction between loci but a binned one; i.e. multiple continuous base-pairs are counted as one interaction. This is referred to as a *resolution*, the fewer base-pairs per bin, the higher the resolution. The presented approach flattens the interaction matrices of a cell to a single dimension. It creates a new matrix where each row represents one cell to use classical clustering algorithms, such as k-means or spectral clustering. The downside of this approach is a high feature number; for example, with 1 megabase (Mb) resolution matrices and the mice mm9 reference genome (https://www.ncbi.nlm.nih.gov/assembly/GCF_000001635.26), 7.6 million features are present while using 10 kilobases (kb) matrices the matrix has 76 billion features.

Dimension reduction is a well-known approach to improve the clustering quality (Deegalla and Boström, 2007; DeTomaso *et al.*, 2019; Lee *et al.*, 2007). Computing a k-nearest neighbors graph, represented as a matrix, is one of them. A k-nearest neighbors graph connects nodes with *k* other nodes, and the edge weights represent the similarity between two nodes. In this work, each cell is considered a node, and the edge weight is the similarity between the two cells. With a k-nearest neighbors graph, the number of features is reduced to the number of cells. The exact k-nearest neighbor’s graph algorithm has a run time of $O({\left(n\times f\right)}^{2})$, with *n* the number of cells and *f* the number of features. As long as *f* is reasonably small, the computation time will mainly depend on the number of cells *n*, but as the number of features rises to the millions, the compute time becomes more dependent on the features rather than the number of cells. Moreover, the higher the features, the less meaningful similarity between two cells is. Both phenomenons are known in the context of the curse of dimensionality (Aggarwal *et al.*, 2001; Bellman, 2015; Beyer *et al.*, 1999; Chen, 2009; Hammer, 1962; Hinneburg *et al.*, 2000; Houle *et al.*, 2010). For many k-nearest neighbor graphs, distance metrics such as the Euclidean distance or similar metrics are used to compute the relation of two instances. In Hi-C, using the Euclidean distance or similar metrics is, in our opinion, problematic. Consider the following: one cell has 0 interactions at a specific location, a second cell has 100 and a third cell 200. Using the Euclidean distance, the first and third cells are equidistant from the second cell. However, in our opinion, the results must be interpreted so that the second and third cells have recorded interactions and are therefore closer to each other than a cell without any interactions. To generalize this argument, Hi-C matrices with similar structures like A/B compartments, TADs or loops should, in our opinion, considered as more similar to each other, independent of the interaction intensity. Metrics like the Euclidean distance cannot guarantee this property; however, due to Hi-C matrices’ very sparse nature, the Jaccard index can provide this. Similar observations concerning the sparsity of the data and the problematical usage of the Euclidean distance have been made in single-cell RNA-seq.

In this article, we propose, therefore, an algorithm to overcome these limitations. A k-nearest neighbor graph is computed to reduce the high number of features with respect to the number of cells. Instead of the problematic Euclidean distance, a measurement with a binary interpretation of the contacts, the Jaccard index, is used. Concerning the expected increasing read coverage and cell number, the quadratic run time to construct the k-nearest neighbor graph is replaced by a linear run time solution. The linearity is achieved by exchanging the Jaccard index by its approximation, MinHash (Broder, 1997), a locality-sensitive hash function technique.

## 2 Materials and methods

The interaction matrices of cells need to be compiled into one interaction matrix to cluster single-cell Hi-C data. Each individual single-cell matrix’s dimensions depend on the used reference genome and the resolution of the Hi-C data. To compile the individual single-cell matrices with (n×n) dimensions to one matrix without losing any information, each interaction matrix is flattened to 1×(n×n) dimensions:

(1) $$\left[\begin{array}{ccc} 1 & 2 & 3\\ 4 & 5 & 6\\ 7 & 8 & 9 \end{array}\right]\to \left[\begin{array}{ccccccccc} 1 & 2 & 3 & 4 & 5 & 6 & 7 & 8 & 9 \end{array}\right]$$

Subsequently all *m* flattened interaction matrices are compiled to one interaction matrix with (m×(n×n)) or $\left(m\times n^{2}\right)$:

(2) $$\begin{array}{c} \begin{array}{c} \left[\begin{array}{ccccccccc} 1 & 2 & 3 & 4 & 5 & 6 & 7 & 8 & 9 \end{array}\right]\\ \begin{array}{ccccccccc} & & & & \vdots & & & & \end{array}\\ \left[\begin{array}{ccccccccc} 1 & 2 & 3 & 4 & 5 & 6 & 7 & 8 & 9 \end{array}\right] \end{array} \end{array}\begin{array}{c} \Rightarrow \end{array}\begin{array}{c} \left[\begin{array}{ccccccccc} 1 & 2 & 3 & 4 & 5 & 6 & 7 & 8 & 9\\ & & & & \vdots & & & & \\ 1 & 2 & 3 & 4 & 5 & 6 & 7 & 8 & 9 \end{array}\right] \end{array}$$

**Fig. 1.**
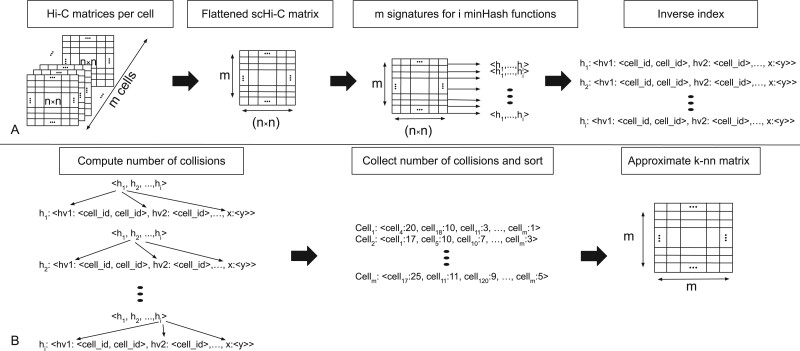
(**A**) Pre-processing and fitting: All *n *×* n* Hi-C matrices of the *m* cells are flattened to one single-cell Hi-C (scHi-C) matrix with m×(n×n) dimensions. For each row a signature is computed and inserted into the inverse index. (**B**) K-nearest neighbors computation: Per signature, the hash function *h_i_* is checked if the hash value at signature index *i* is present in the inverse index. If such a collision is detected, the associated cell ids are stored. After all hash functions are checked, the number of occurrences for the cell_ids is counted and sorted. This order gives the nearest neighbor’s relationship

Figure 1(A) provides an abstract graphical description.

This new compiled single-cell Hi-C matrix can be used to apply well-known clustering algorithms like k-means or spectral clustering directly. However, research on the curse of dimensionality shows that the more features are available, the less meaningful a similarity is (Aggarwal *et al.*, 2001; Beyer *et al.*, 1999; Hinneburg *et al.*, 2000). Our approach reduces the number of features before a clustering algorithm is applied. For this, we compute a k-nearest neighbors graph using the approximation of the Jaccard index, MinHash, as a similarity measure. Subsequently, a principal component analysis (PCA) and a UMAP embedding (McInnes *et al.*, 2020) are used to reduce the dimensions of the k-nearest neighbor’s graph to low dimensional space.

### 2.1 Jaccard index

The Jaccard index of two cells is given by their sets *A*, *B* of non-zero feature ids. A non-zero feature id is the feature index position of a feature which cell has at its index at least one recorded Hi-C interaction.

(3) $$J(A,B)=\frac{\left|A\cap B\right|}{\left|A\cup B\right|}$$

Based on the Jaccard index, the similarity between two cells in terms of how many features they share can be used to compute a k-nearest neighbors graph where the edge weight is the similarity. However, the computation of a k-nearest neighbors graph is in $O(n^{2})$. Its approximation replaces the Jaccard index with MinHash (Broder, 1997) to compute in linear time.

### 2.2 MinHash

Cells which share features are more likely to be similar to each other compared to cells with less common features. MinHash uses this fact; for each cell, only a set of features’ id *A* of non-zero features (non-zero Hi-C interactions) are considered (similar to Heyne *et al.*, 2012), and the hash value per MinHash function *h* is computed as the argmin over all non-zero features a∈A of a hash function *f*. A set of MinHash functions *H* and hash functions *F* are used; h∈H and f∈F. The similarity between two cells is computed by counting the number of collisions overall MinHash functions.

(4) $$h(A)=\mathrm{argmi}n_{a\in A}f(a)$$

Broder shows that MinHash is an unbiased estimator of the Jaccard index:

(5) P(h(A)=h(B))=(|A∩B|)/(|A∪B|)=J(A,B)

### 2.3 Clustering

Multiple options are available to process the Hi-C contacts to compute the k-nearest neighbor’s graph with MinHash. The first option uses inter- and intra-chromosomal contacts; the second option only intra-chromosomal contacts. The first option has the benefit of considering potential important long-range contacts; however, distinguishing them from noise is only possible with a high read coverage. It might be, therefore, beneficial for the cluster results to consider only intra-chromosomal contacts. The parameters used to compute the k-nearest neighbor’s graph are the number of employed hash functions and, therefore, how many collisions occur. The number *k* of neighbors to be computed and if the additional Euclidean distance based on the pre-selection of candidates should be considered. The number of features of the k-nearest neighbor graph is still considered as high dimensional. A principal component analysis followed by a UMAP embedding is applied before the clustering to reduce the number of dimensions further. For the clustering algorithms, we use the algorithms offered by scikit-learn (Pedregosa *et al.*, 2011) and limit ourselves to the clustering algorithms that support a user-specified fixed number of clusters. These are K-means, spectral clustering, birch and agglomerative clustering.

### 2.4 Implementation

#### 2.4.1 Inverse index

Fast computation of a k-nearest neighbors graph requires a linear query time and a significant reduction of the number of features to overcome the curse of dimensionality. A regular index stores the computed hash values of a hash function per cell, leading to $O(n\times n\times h)\in O(n^{2})$ to create the k-nearest neighbor graph. In order to reduce the construction to linear time, an *inverse index* is used. Per hash function, the hash values with the corresponding cell id are stored. To construct a k-nearest neighbors graph, for each cell, the hash functions have to be checked for collisions which is per hash function in *O*(1) and for all cells O(n×h)∈O(n).

#### 2.4.2 Fitting

**Fig. 2.**
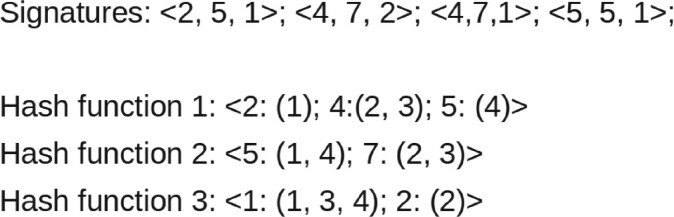
An example signature and inverse index: The signature is created for four cells and three hash functions. The inverse index stores the computed hash value and the id of the cell for each hash function. For example, for the second cell <4,7,2> the first hash function Hash function 1 stores the computed hash value *4* and associates the id of the cell: Hash function 1:<2:(1);4:(2,3);5:(4)>. The same hash function and hash value occur for cell number three again; this is a *collision* of hash function 1 for cell 2 and cell 3

The MinHash values of all hash functions together are called the *signature* of the cell; these signatures are inserted into the inverse index to achieve a fast query time. The run time of the fitting depends on the number of cells *n*, the number of hash functions *h* and the number of non-zero features per cell *f* and is given as O(n×h×f)∈O(n). For an example of the fitting and the inverse index structure, refer to Figure 2.

#### 2.4.3 Collision based approximate nearest neighbors graph

The number of *hash collisions* between two cells gives an estimate of their similarity. The signature of a cell is used to search for *hash collisions* in the inverse index to compute the estimate. A *hash collision* between two cells is defined as the same hash value for the same hash function. The more collisions two cells have, the more similar they are. The query time of this approach depends only on the number of used hash functions and, if not stored in memory from the fitting phase, the computation of signatures. The effect of sorting all occurrences of collisions and the query time of the used data structures of the inverse index on the run time should also be considered, although it is negligible from the user’s point of view.

#### 2.4.4 Technical implementation

For this implementation, we use the hash function ‘32 bit mix function’ designed by Thomas Wang (https://gist.github.com/badboy/6267743#32-bit-mix-functions) published in 1997/2007. The hash function is always the same; however, for each hash function f∈F the seed differs. The index values for a 10 kb resolution matrix exceed the data range of 32-bit by 5 bit. The index values are modified via modulo operation to fit the 32-bit range to avoid a 64-bit hashing. The sparsity of the data is advantageous and results in more than 98% unique indices after the modulo operation. To compute the approximate nearest neighbors with MinHash, a highly optimized library, ‘sparse-neighbors-search’, was implemented in C++ with SSE and OpenMP support. To ensure user accessibility, the C++ library is embedded in a Python 3.6, 3.7 and 3.8 interface. The MinHash approximation of a k-nearest neighbors graph is part of the scHiCExplorer (Wolff *et al.*, 2020a); a software to process, analyze and visualize single-cell Hi-C data.

## 3 Results

The algorithm is tested with differing properties and settings to evaluate the clustering abilities of the proposed algorithm. The clustering is tested on the matrices at different levels of processing. Compared here is the ability to detect the different cell cycle phases (Nagano *et al.*, 2017) respectively the cell types (Ramani *et al.*, 2017) based on the low dimensional embedding of the Hi-C cells. First, the MinHash approach and its differentiation ability is discussed. Second, the best settings for the algorithm are investigated, and third, the proposed solution is compared to the competing algorithm *scHiCluster* from Zhou *et al.* (2019); also a clustering based on a principal component analysis on the raw matrices, and a k-nearest neighbor graph computed with scikit-learns implementation are considered.

### 3.1 Embedding and differentiability of MinHash

**Fig. 3.**
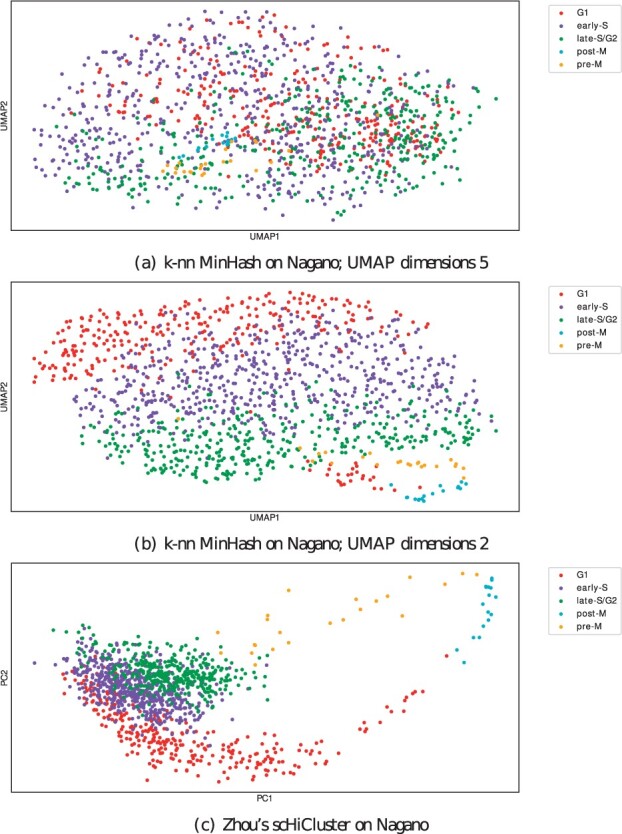
Embedding into a two dimensional space based on cell cycle data from Nagano *et al.* (2017). Computed on 1275 cell cycle phase cells with their cell cycle phase label. (**a** and **b**) are computed with the proposed algorithm. (a) is with 5 UMAP dimensions and plotted with the first two, (b) uses the same parameters but with two UMAP dimensions. The second approach is better for a visualization, however, Supplementary Tables S1 and S2 clearly indicate the clustering result with the first approach is better. (**c**) shows the first two principal components of Zhou’s scHiCluster

**Fig. 4.**
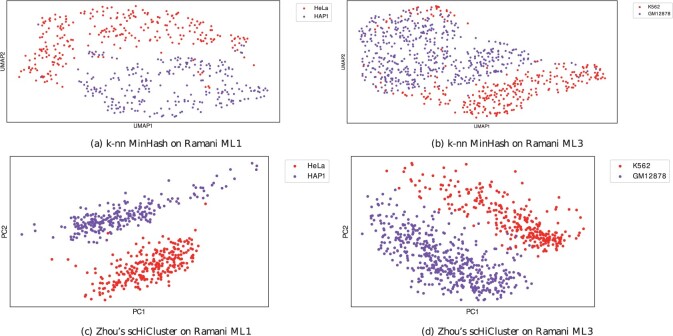
Embedding into a two dimensional space for cell type data from Ramani *et al.* (2017). Separated by the two batches ML1 (**a** and **c**) and ML3 (**b** and **d**) and labeled by their cell types

The Jaccard index-based approach with its approximation via MinHash, combined with a consecutively PCA and UMAP embedding for a further dimension reduction, provides good differentiability of the test data. The 1 MB cell cycle data from Nagano *et al.* (2017) shown in Figure 3a is reduced to five UMAP components and visualized are the first two dimensions. The visualization with the first two UMAP dimensions is not indicating a good clustering result (Fig. 3a), but an embedding with the same parameters, but reducing to two UMAP dimensions instead of five, improves this (Fig. 3b). However, the clustering results of this approach are not as good as for the five UMAP dimensions (Supplementary Tables S1 versus S2). Early-S (purple), late-S/G2 (green) and G1 (red) cell cycles are differentiated, and post-M (cyan) and pre-M (yellow) are projected to a similar location; an overlap of the different cell cycle phases is given. Good clustering results are confirmed by validating the detect clusters by Nagano *et al.* (2017) provided cell cycle labels (Supplementary Table S1). A batch effect is slightly visible (Supplementary Fig. S1a) but is not dominating. The 1 MB cell type data from Ramani *et al.* (2017) are displayed in Figure 4a and b. The four cell lines are provided from two batches, and a strong batch effect is visible (Supplementary Fig. S2a). The embedding of the ML1 batch with HeLa and HAP1 cells show a clear differentiation of the two cells (Fig. 4a), and the ML3 batch with K562 and GM12878 provides a good differentiation too (Fig. 4b). However, the ML3 embedding has some minor issues: K562 cells are projected to the top to the area of GM12878 cells. It requires further investigation if this is an error by the embedding approach or if, as the spatial separation indicates, further subtypes are present within the dataset. Ramani *et al.* (2017) provides only the cell type labels, but it is not unlikely that the cell type data itself contains cells with a different cell cycle phase.

### 3.2 Jaccard versus Euclidean distance

The proposed algorithm’s primary aim is to reduce the high dimensional space of the single-cell Hi-C data from millions and billions of dimensions to a lower-dimensional space to improve the clustering abilities. This involves several dimension reduction steps: The reduction of the single-cell Hi-C interaction data via a k-nearest neighbors graph to (*cell* × *cell*) dimensions. The two measures to compute the k-nearest neighbor graph, namely the approximate Jaccard index and Euclidean distance, have a different impact on the embedding results. On the 1275 cells from Nagano *et al.* (2017) with a 1 Mb resolution and the five pre-classified cell cycle phases (G1, early-S, late-S/G2, post-M and pre-M), the approximate Jaccard index can create a distinguishable clustering, while the Euclidean based approach falls behind in terms of accuracy. For example, for an accuracy level of at least 70% of uniquely classified cells of a cell phase per cluster: the Jaccard index-based approach detects 73% of G1, 61% of early-S, 87% of late-S/G2, 94% of post-M and 91% of pre-M; while for the Euclidean distance only 37% of G1, 35% of early-S and 32% of late-S/G2 and both post-M and pre-M are not detected (Supplementary Tables S1 and S3). The Euclidean distance’s performance can be explained by its behavior in high dimensions (Aggarwal *et al.*, 2001; Beyer *et al.*, 1999; Hinneburg *et al.*, 2000). Moreover, the Euclidean distance does not differ between no-contacts and contacts, whereas the Jaccard index, on the other hand, exactly makes this distinction and is, therefore, more suitable.

### 3.3 Embedding via UMAP with and without prior PCA

The principal component analysis reduces the matrix dimensions from (*cells* × *cells*) to a user-defined number of components (PC) (cells×|PC|). The problem of not using a principal component analysis is present for the pre-M and post-M cells: The post-M cells are mixed with pre-M cells (cluster 10), and the pre-M cells vanish in cluster 4, which is dominated by late-S/G2 cells (Supplementary Table S4). Third, using UMAP in combination with the metric ’Canberra’ (Lance and Williams, 1966) reduces the number of dimensions to a user-defined number of UMAP components (UMAP_COMP) with |PC|>|UMAP_COMP|: (cells×|UMAP_COMP|). This creates better clustering results in comparison to the dataset that was only using principal component analysis (Supplementary Tables S1 versus S4). Performing no principal component analysis followed by UMAP has a worse detection rate and does not recognize any pre-M and post-M cells (Supplementary Table S5). The situation is identical if the clustering is directly applied to the approximate k-nearest neighbor’s graph without an additional PCA and UMAP embedding (Supplementary Table S6).

### 3.4 Other parameters properties

The ideal parameter setting to compute the approximate k-nearest neighbor graph is investigated; it is beneficial to initially use only intra-chromosomal contacts (Supplementary Table S7), as well as more hash functions to contribute to a better differentiation (Supplementary Tables S8 and S9). In this context, the density of a matrix is also essential. For example, the density distribution of the cells in a 30 Mb context around the main diagonal of a 1 kb matrix (from Gassler *et al.*, 2017) with a density of 0.000002 is too sparse to create a substantial amount of hash collisions, independent of the number of hash functions used (Supplementary Figs S5–S13). It is beneficial to compute a full k-nearest neighbor graph and not, e.g. a 100-nearest neighbor or a 1000-nearest neighbors graph (Supplementary Tables S10 and S11). Last, the method to cluster the data is investigated; spectral clustering is compared to the other tested approaches, the algorithm with the best precision (Supplementary Tables S1 and 12–S17).

### 3.5 Comparison with competing approaches

The differentiation ability of the proposed algorithm is, compared to Zhou’s scHiCluster, on a more advanced level. Considering a unique level of 70% of a cell phase per cluster, Zhou’s scHiCluster detects 53% of G1 (versus 73%), 50% of early-S (versus 61%), 54% late-S/G2 (versus 87%) and is not able to detect any of the pre-M and post-M cells. Considering a uniqueness level of 80%, Zhou’s scHiCluster detects more G1 cells (53% versus 51%) but less early-S (50% versus 60%), late-S/G2 (54% versus 87%), post-M (0% versus 94%) and pre-M (0% versus 91%); consider Supplementary Tables S1 and S18. For both embedding approaches, a distorted relation of the number of cells from each cell phase could be problematic. Three phases are present 1235 out of 1275 times (G1 300, early-S 573, late-S/G2 362), while post-M is present 17 and pre-M 23 times.

Besides the clusters with a high amount of a unique cell phase, the clustering result shows that mixed clusters do not have a random structure but represent the cell cycle’s dynamic process. Cluster 5 of the proposed algorithm contains 11% of early-S and 88% late-S cells; Cluster 2, 6, 8 and 10 a mix of G1 and early-S cells. The two major phases in each cluster are consecutive in the cell cycle, and a strict separation with no overlaps of phases would be an unexpected result.

A batch effect is slightly visible (Supplementary Fig. S1a–c) but does not dominate the differentiation of the embedding. Furthermore, the detection rates of the clustering directly applied on a k-nearest neighbor graph computed by scikit-learn’s implementation (Supplementary Table S19), on a principal component analysis reduced dataset (Supplementary Table S20) or on the raw data (Supplementary Table S21) are significantly worse and cannot compete with the proposed algorithm. The embedding on 10 kb resolution is different. While Zhou’s scHiCluster cannot perform the computation within a reasonable time nor operate within generous memory requirements (Supplementary Tables S22 and S23), the proposed algorithm has significant issues distinguishing the cell phases. Two cell cycle phases (early-S and late-S/G2) are partially differentiated; however, they have significant overlaps with each other, especially for the G1 phase, and not all are embedded in a particular region. Post-M cells are embedded into one region, but the pre-M cells are distributed over the embedding, with no exact region, and therefore, no clustering can be achieved for this cell cycle phase (Supplementary Tables S24–S26). Investigating the batch relation shows no correlation between the batch and the embedded region (Supplementary Fig. S3). The bad detection rate can be explained by a too sparse dataset with a density of 0–0.0006 (Supplementary Fig. S6 (right)). Even a high number of hash functions does not help to create a meaningful similarity between the cells (Supplementary Tables S24–S26 with 20 000; 40 000 and 50 000 hash functions).

Considering the different cell type data from Ramani *et al.* (2017), both the proposed algorithm and Zhou’s scHiCluster show a separation by the two batches, ML1 and ML3 (Supplementary Fig. S2a and b). For this reason, the cells of the two batches are separately computed. While per batch, only two cell types are present (ML1: HeLa and HAP1; ML3: K562 and GM12878), the results indicate subtypes in the data. Both Zhou’s scHiCluster and the proposed algorithm benefit from using more clusters. For ML1, the proposed algorithm outperforms Zhou’s scHiCluster if two clusters are used: Considering a uniqueness of at least 70%, the proposed algorithm detects 91% of HeLa cells and 94% of HAP1, while Zhou’s scHiCluster detects 72% for both cell types. A uniqueness level of 80% or 90% keeps the results at an equivalent level for the proposed algorithm but let it drop to 0% for Zhou’s approach. However, the situation is different if three clusters are used: at a level of 90%, the proposed algorithm detects 95% of HeLa and 92% of HAP1 while Zhou’s approach detects 96% and 100% (Supplementary Tables S27 and S28). The situation is similar for ML3: Using two clusters, the proposed algorithm detects slightly more cells for GM12878 (73% versus 72%), but both detect 0% of the K562 at a uniqueness level of 70%. Using five clusters shows an advantage of Zhou’s scHiCluster, where it detects 94% for K562 and 98%for GM12878 at a uniqueness level of 80%; the proposed algorithm detects 78% for K562 and 94% for GM12878 (Supplementary Tables S29 and S30). Working on 10 kb data from Ramani *et al.* (2017), Zhou’s scHiCluster cannot compute it within a reasonable time and memory constraints; however, the results of the proposed algorithm are mixed. A batch effect of ML1 and ML3 is visible (Supplementary Fig. S4), but a clear differentiation of the cell types not (Supplementary Tables S31 and S32). A differentiation of more extensive parts of the GM12878 cells for a uniqueness level of 70% is possible with 74%, but upon a closer investigation of the clusters, it is evident that a high mixture of the cells is given. This is especially true for ML1, and the cell types HeLa and HAP1, where no clear differentiation is possible. We assume the density of 0–0.00004 for most of the cells (Supplementary Fig. S6 (right)) is too sparse to create a good nearest neighbors computation.

### 3.6 Contact decay profiles

**Fig. 5.**
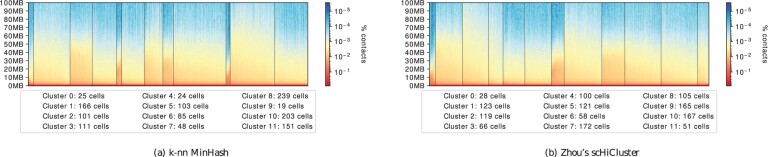
Contact decay profile of the clusters; computed by scHicClusterMinHash with spectral clustering (**a**), Zhou’s scHiCluster (**b**). Computation on 1 Mb resolution, with 1275 cell cycle phase cells from Nagano *et al.* (2017). Numbers indicate the cluster id and how many cells they contain; clusters are to be read from left to right. The number of clusters is 12 to have comparability to the cluster results of Nagano *et al.* (2017)

Contact decay profiles show for each cell in a given cluster the summed number of contacts per genomic distance. Each row is the genomic distance between the Hi-C contacts’ two locations, and the columns are the cells. It is the nature of Hi-C contacts to decay with increasing distances between the two locations. Moreover, the decay of contacts should have a similar pattern for each detected cluster since the clusters’ cells are sorted by the short to long-distance contact ratio. The plot gives a global indication of the detected clusters’ correctness but incorrectly detected individual cells vanish. Figure 5 shows a contact decay plot based on the cluster results as shown in Supplementary Tables S1 (the proposed algorithm) and S18 (Zhou’s scHiCluster approach) on the cell cycle data from Nagano. Both results are very similar from a global perspective. Clear contact decay patterns within the clusters and differences to the other clusters are visible, indicating the dimension reduction, embedding and clustering are general functional. It should be noted that the proposed algorithm can detect the post-M (cluster 9) and pre-M (cluster 0), while Zhou’s scHiCluster mixes both (cluster 0) or mixes it with late-S/G2 cells (cluster 4). In contrast to these results are the contact decay profiles, where clustering was performed on: the raw interaction matrices (Supplementary Fig. S14a), a Euclidean distance-based k-nn (Supplementary Fig. S14g, h), the proposed algorithm using the Euclidean distance (Supplementary Fig. S14b), without an intermediate principal component analysis (Supplementary Fig. S14d) or without UMAP (Supplementary Fig. S14e). All the results in Supplementary Figure S14 have significant differences in the contact decay within the clusters, clearly indicating an overlap of cells that are not consecutive in the cell cycle.

### 3.7 Consensus matrices

**Fig. 6.**
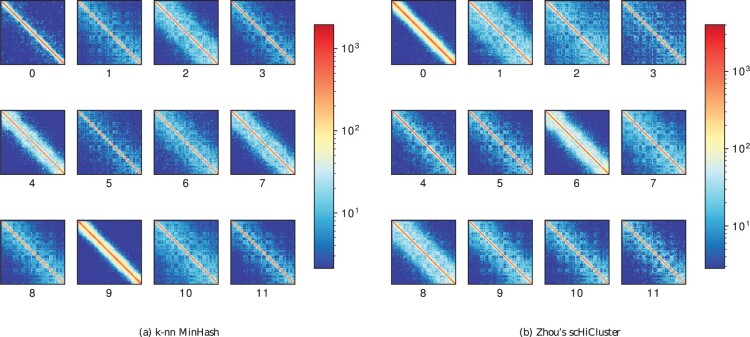
Consensus clusters computed by scHicClusterMinHash with spectral clustering (**a**) and Zhou’s scHiCluster (**b**). Computation on 1 Mb resolution, with 1275 cell cycle phase cells from Nagano *et al.* (2017), with 12 clusters. Numbers under the matrices indicate the cluster id. The number of clusters is 12 to have comparability to the cluster results of Nagano *et al.* (2017)

A consensus matrix is a bulk mean Hi-C matrix of all cells of a cluster. In the best case, all cells of a cluster have a similar chromatin pattern and provide an insight into the chromatin folding properties. The more noisy a consensus matrix is, the more likely the cells from different cell cycle phases or cell types are merged into the same cluster. Figure 6 shows the consensus matrices for chromosome 6 based on the clusters presented in Supplementary Tables S1 and S18. For both the proposed algorithm and Zhou’s scHiCluster, different Hi-C contact matrix patterns and, therefore, different chromatin folding properties are well developed. Given a uniqueness of >80% as shown in Supplementary Tables S1 and S18, the cell cycle stage G1 is represented by clusters 2, 4 and 7 for the proposed algorithm and clusters 6 and 8 for Zhou’s scHiCluster. The patterns for the clusters are similar, and the same is true for the early-S clusters from us (1 and 8) and Zhou (7 and 9), late-S/G2 (3, 5 and 11 respectively 4 and 5); however, post-M and pre-M are identified by the proposed algorithm (cluster 0 and 9), where Zhou’s scHiCluster instead mixes post-M and pre-M cells in cluster 0. A closer look at the consensus matrices for the other investigated approaches confirms the findings of the previous sections that the usage of raw data, the euclidean distance, a 100-nearest neighbors graph, no PCA, no UMAP, inter- and intra-chromosomal contacts, or the usage of the scikit-learn k-nn do not lead to a good differentiation of the cell cycles (Supplementary Figs S15 and S16).

### 3.8 Runtime and memory

The approximate Jaccard index computation achieves faster or similar run times compared to the sklearn implementation of a k-nearest neighbors search, which is based on ball-trees, under consideration of the one megabase resolution single-cell Hi-C dataset as shown in Supplementary Tables S33 and S34. The runtimes can be influenced by the clustering algorithm used. This is especially the case for the clustering on raw data where k-means runs for around 40 min; for all others, a difference is present but is minor. However, we cannot explain the outstanding runtime of k-means on the XEON machine; a run on the AMD Ryzen based computer shows a runtime of 12 min, but a similar runtime for all other algorithms. The classical and naive way to reduce dimensions is a principal component analysis (PCA), but the method uses a high amount of memory (170 GB) even on the low-resolution matrix. For the 10 kb resolution matrix, the PCA method throws an error that it is *’unable to allocate 1.28 PiB for an array with shape (2633, 69647960281) and data type float64’*. All approximate k-nearest neighbor graph approaches use a similar amount of memory caused by the memory consumption at the read-in stage of the individual single-cell Hi-C matrices. The provided Euclidean mode of the proposed algorithm has a little increased run time compared to sklearns implementation. The runtimes of the proposed algorithm computed on a state-of-the-art computer with an SSD compute the clustering on the low-resolution matrix in around a minute and uses less than 8 GB of memory. The clustering on the high-resolution matrix is computed in 3:30 min and uses 40 GB of memory (compare Supplementary Tables S22 and S23). To have reduced memory usage, the mode *–saveMemory* is offered. The proposed algorithm uses one core to load in a batch processing way data; the user can define the share of the to be processed matrices. The 10 kb resolution matrices’ processing with a share of 1% of the data took 13 min, but the memory usage is reduced to 12.5 GB (Supplementary Table S23). The more hash functions are used, the longer the run times are. The runtimes on a 1 Mb resolution using only cells with available labels is faster compared to Zhou’s scHiCluster even for 20 000 hash functions (Supplementary Table S35).

Zhou’s scHiCluster has a runtime of 14 min with the CPU implementation and 7 min on the GPU on a low resolution (1 Mb) matrix (Supplementary Table S34). The benefits of the proposed algorithm in terms of runtime and memory usage are significant under the consideration of a high-resolution single-cell Hi-C dataset. As shown in Supplementary Table S22, all methods besides the proposed algorithm cannot be computed due to their high memory usage of more than one terabyte. Considering Zhou’s scHiCluster, we canceled the computation after 97 h runtime; the computation of the first loaded chromosome (chromosome 10) was not finished but had a peak memory usage of 970 GB. The data for Zhou’s scHiCluster was stored in a RAM disk to exclude potential network file system issues. Only the proposed algorithm can compute a result while using a moderate 40 GB of memory; these resources are available for most researchers.

## 4 Discussion

It was shown that an approximate k-nearest neighbors graph can be used to reduce the number of dimensions required to cluster single-cell Hi-C data, with higher accuracy, faster run times and enabling users to analyze high-resolution data with a vastly reduced memory burden. The approximation of the Jaccard index proves to be a suitable similarity measure to create a base for clustering, while the Euclidean distance, considering the curse of dimensionality and the unique properties of Hi-C data, is shown to be not such an appropriate measure. The cluster results based on the approximate k-nearest neighbors with MinHash, the additional PCA on the computed k-nearest neighbor’s graph, the UMAP embedding and a spectral clustering show a better differentiation of the chromatin folding properties compared to competitive methods. The presented approach to reduce the number of features, especially when dealing with millions to billions of dimensions, is crucial to achieving adequate run time and memory usages. Access to computers with more than 1 TB of memory is currently difficult, but access to computers or cluster nodes with 40 GB of memory is available to most researchers. The presented approximate nearest neighbors graph enables a broader range of researchers to work with single-cell Hi-C data and adds with the approximate Jaccard index a method to create a k-nearest neighbors graph. Moreover, the proposed algorithm is embedded into the scHiCExplorer, a software suite for single-cell Hi-C data analyses, and supports the native single-cell Hi-C format *scool* (Wolff *et al.*, 2020b). Thanks to the availability of the approximate k-nearest neighbor search as an independent software package, it can be easily integrated into other research issues dealing with similar matrix properties, as is the case in single-cell RNA-seq.

## Supplementary Material

btab394_Supplementary_Data
