# Supplementary material for: Robust and efficient single-cell Hi-C clustering with approximate k-nearest neighbor graphs
Source: Bioinformatics. 2021 May 22;37(22):4006–13. doi: 10.1093/bioinformatics/btab394 (PMC9502147; doi:10.1093/bioinformatics/btab394)
Supplement: btab394_Supplementary_Data [file btab394_supplementary_data.pdf]

Supplementary material: Robust and efficient single-cell Hi-C clustering with approximate k-nearest neighbor graphs

Joachim Wolff[1*], Rolf Backofen[1,2], Björn Grüning[1]

[1] Bioinformatics Group, Department of Computer Science, University of Freiburg, Georges-Köhler-Allee 106, 79110 Freiburg, Germany
[2]Signalling Research Centre CIBSS, University of Freiburg, Schänzlestr. 18, 79104 Freiburg, Germany

*To whom correspondence should be addressed.

1 Batch effects

1.1 Nagano 1 Mb

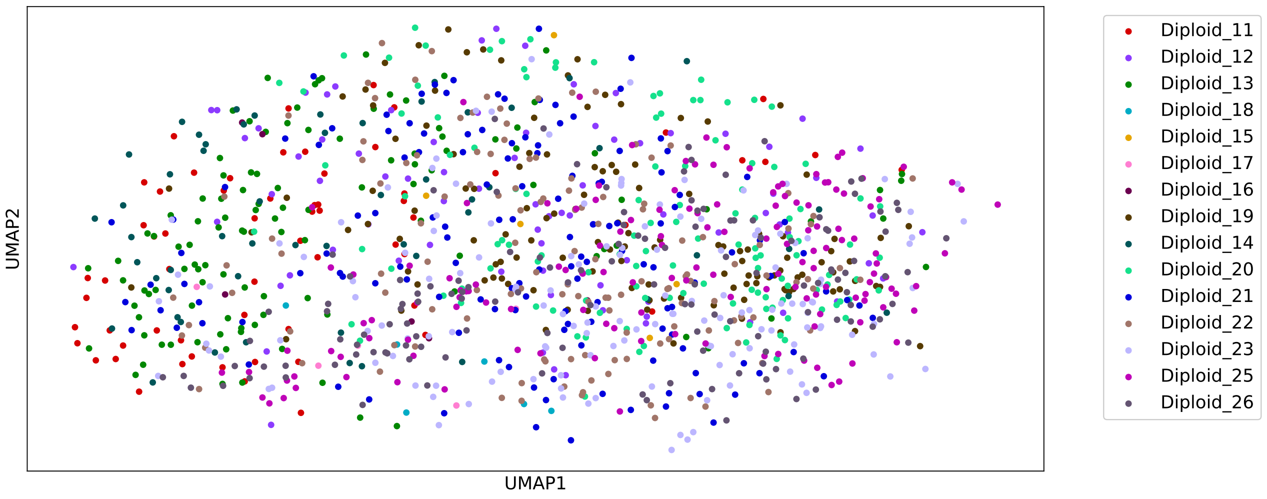

(a) k-nn MinHash on Nagano, UMAP dimensions 5

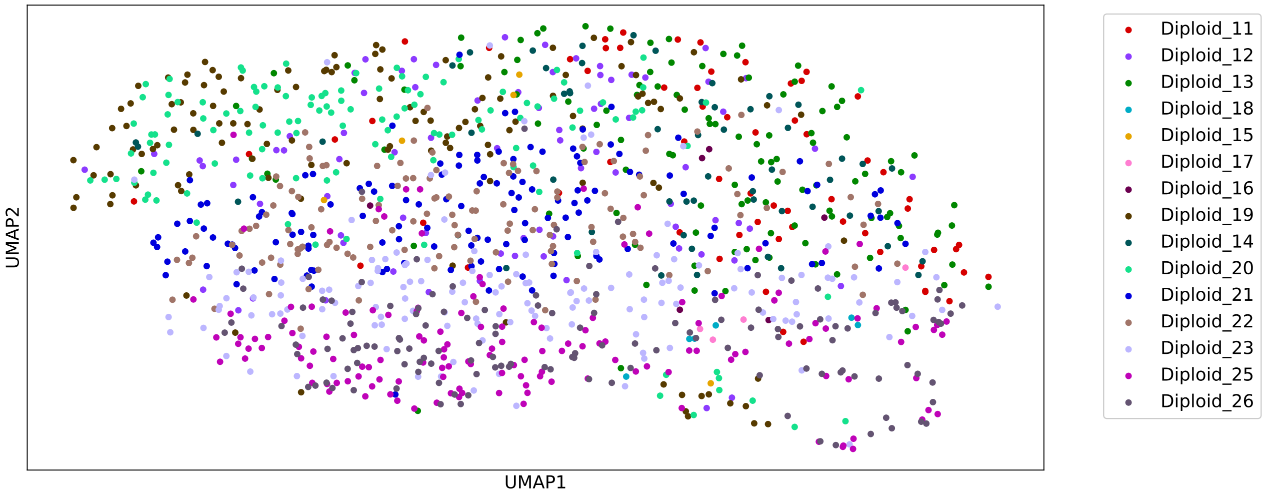

(b) k-nn MinHash on Nagano, UMAP dimensions 2

Figure S 1: Batch effects on Nagano 1 MB

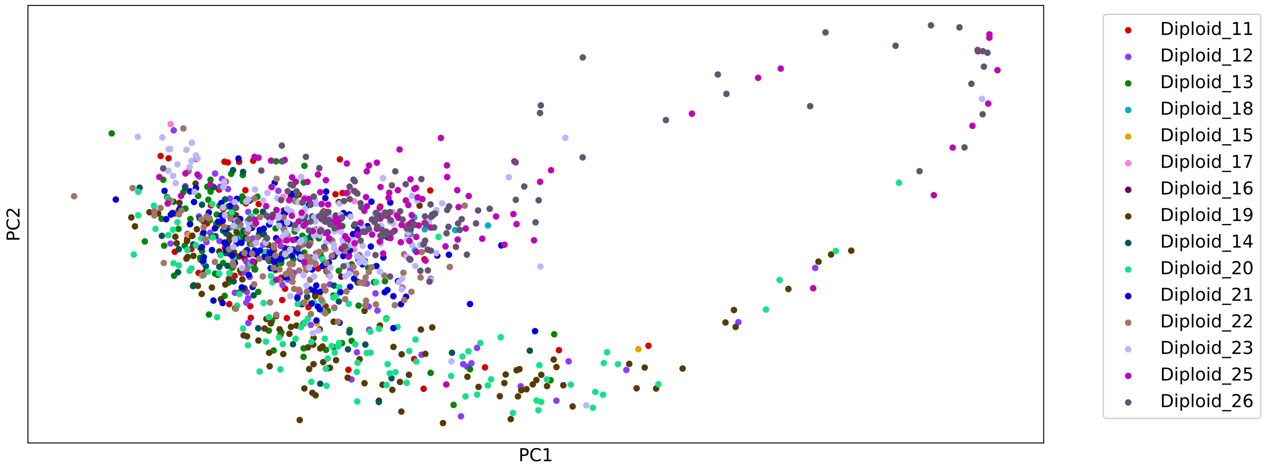

(c) Zhou's scHiCluster on Nagano

Figure S 1: Batch effects on Nagano 1 MB

1.2 Ramani 1 Mb

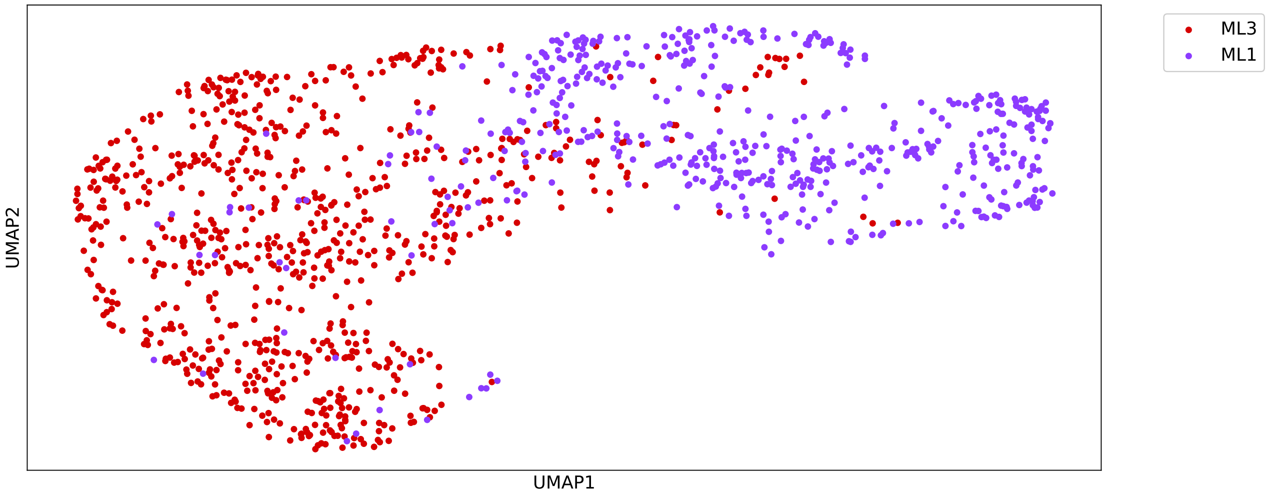

(a) k-nn MinHash on Ramani ML1 and ML3

Figure S 2: Batch effects

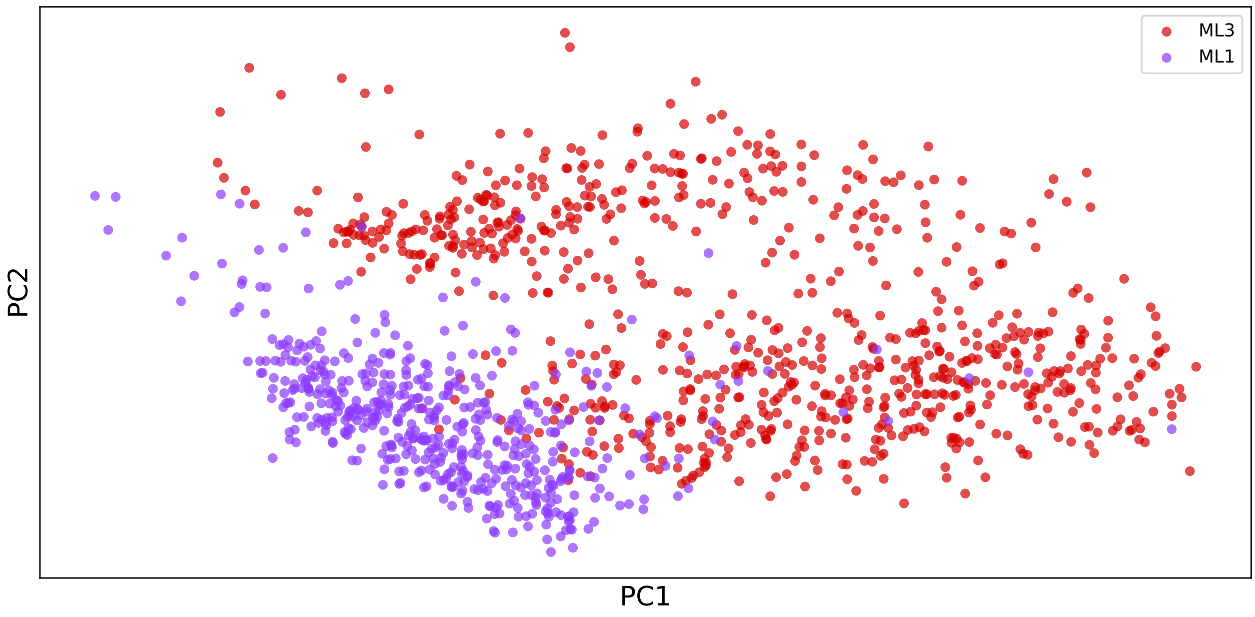

(b) Zhou's scHiCluster ML1 ML3

Figure S 2: Batch effects

1.3 Nagano 10 kb

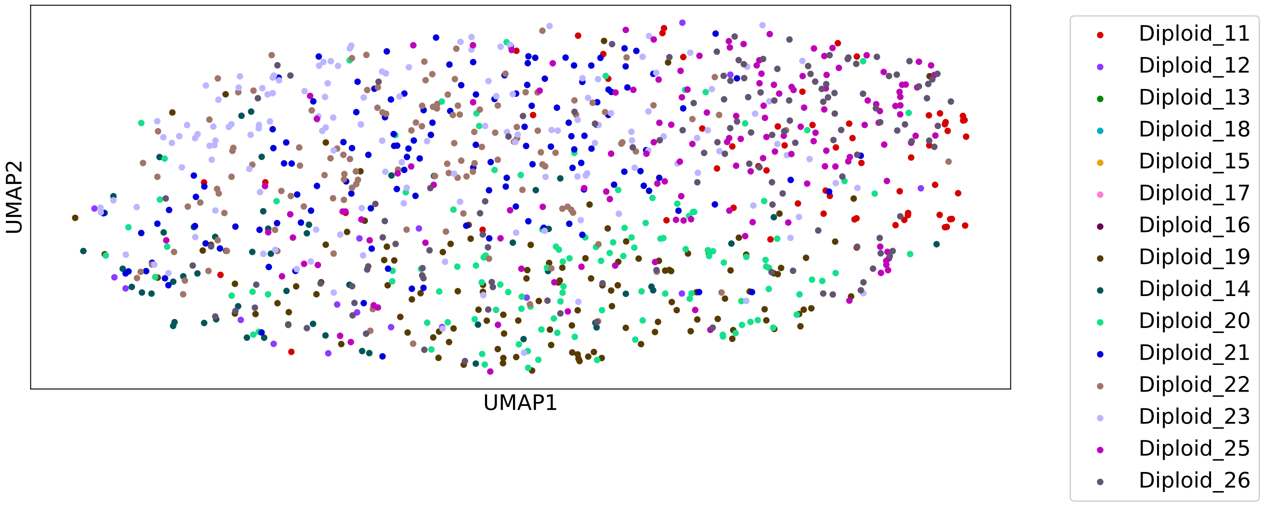

(a) k-nn MinHash on Nagano 10 kb resolution

Figure S 3: Batch effects

1.4 Ramani 10 kb

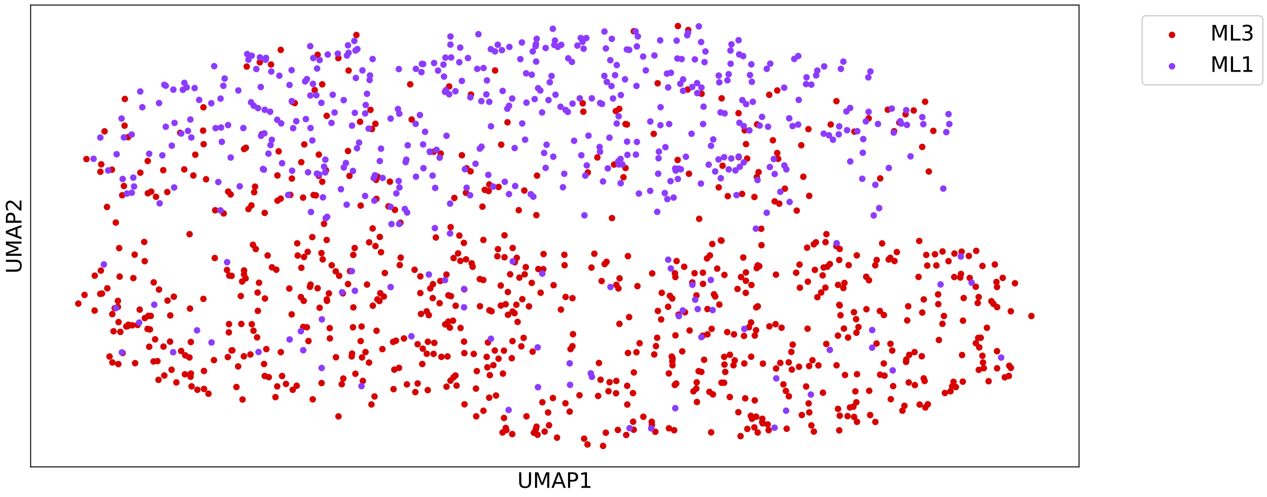

(a) k-nn MinHash on Ramani 10 kb resolution

Figure S 4: Batch effects

2 Density distributions for single-cell interaction matrices

The density of a cell is measured by the number of binary contacts a cell has vs. the number of contacts it could have, i.e., the number of non-zero values of a matrix vs. all values. Single-cell Hi-C matrices have the disadvantage, especially in comparison to regular Hi-C, that their read coverage with around 100,000 reads per cell is low. The majority of the contacts are recorded in close proximity, i.e., around the main diagonal. For these reasons, the density measure is restricted to (possible) interaction pairs within a distance of 30 Mb.

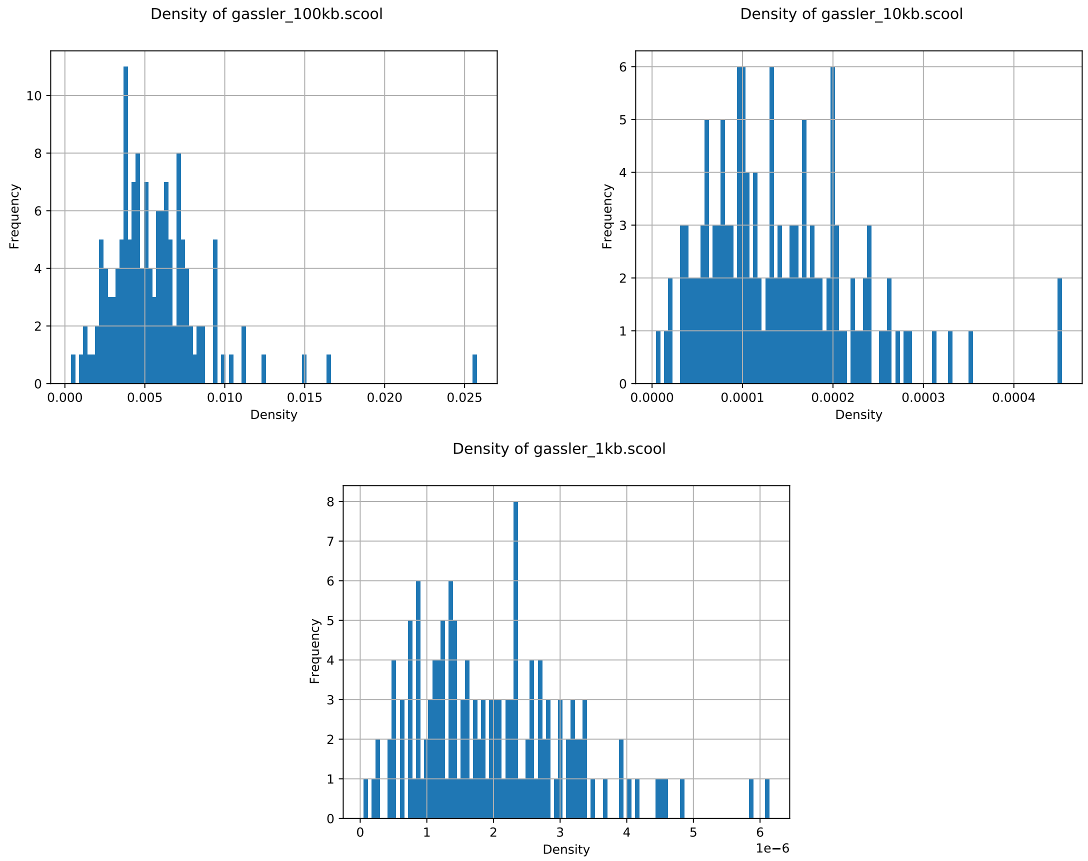

Figure S 5: Density distributions for 100kb, 10kb and 1kb for 144 cells from Gassler *et al.* (2017)

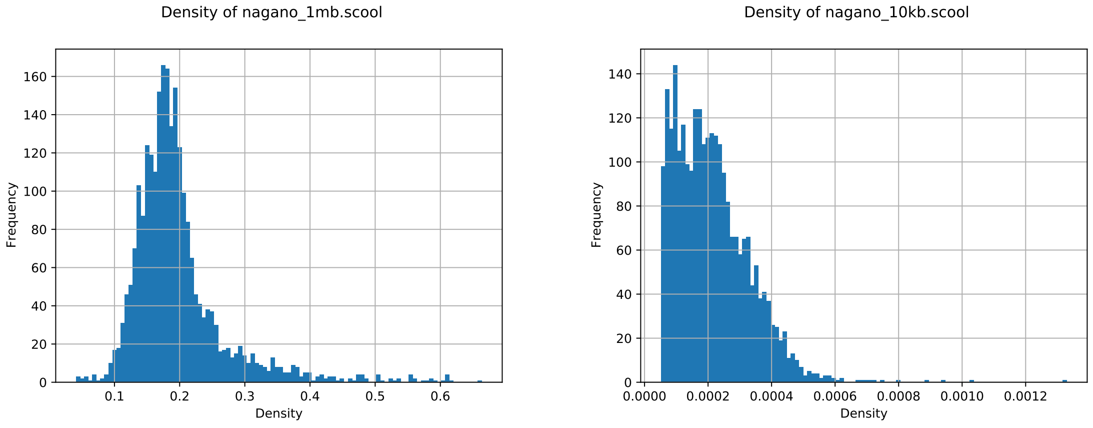

Figure S 6: Density distributions for 1Mb and 10kb for 2472 cells from Nagano *et al.* (2017).

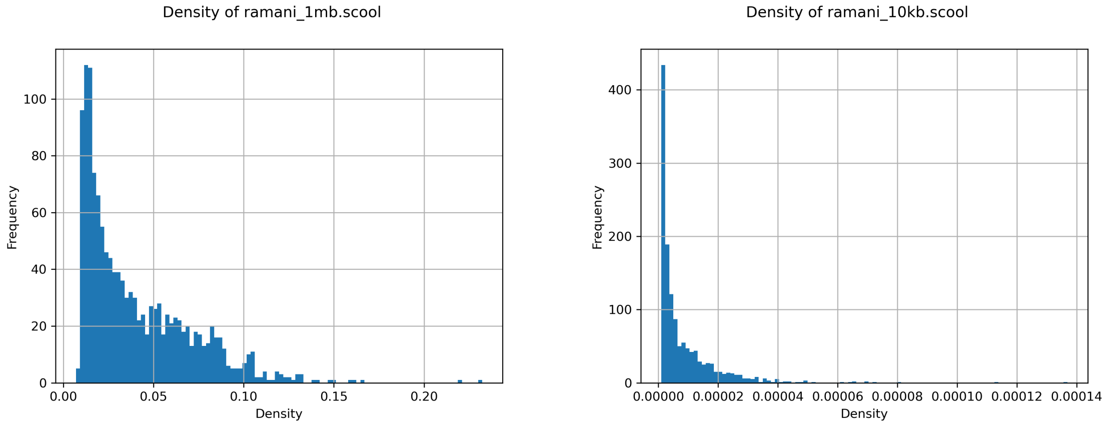

Figure S 7: Density distributions for 1Mb and 10kb for 1329 cells from Ramani *et al.* (2017).

3 MinHash collision statistics

3.1 Collisions per cell

The here shown collision statistics are for different interaction matrix resolutions from Nagano *et al.* (2017) and Gassler *et al.* (2017).

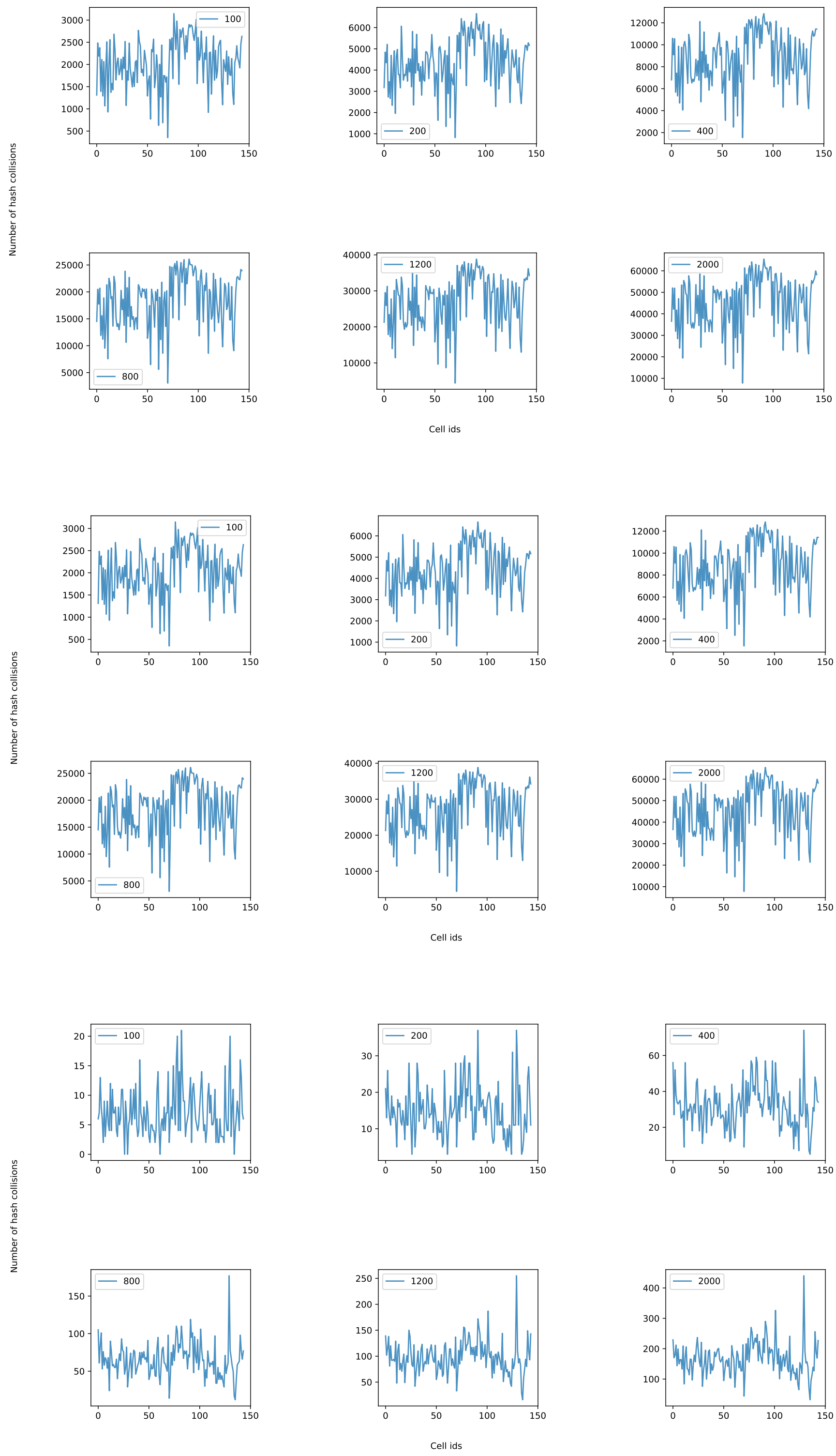

Figure S 8: Number of hash collisions per cell for 100kb (top), 10kb (middle) and 1kb (bottom) for 144 cells from Gassler *et al.* (2017). The number of hash collisions is shown for 100, 200, 400, 800, 1200 and 2000 hash functions. One collision occurs if two cells have the same hash value for a hash function.

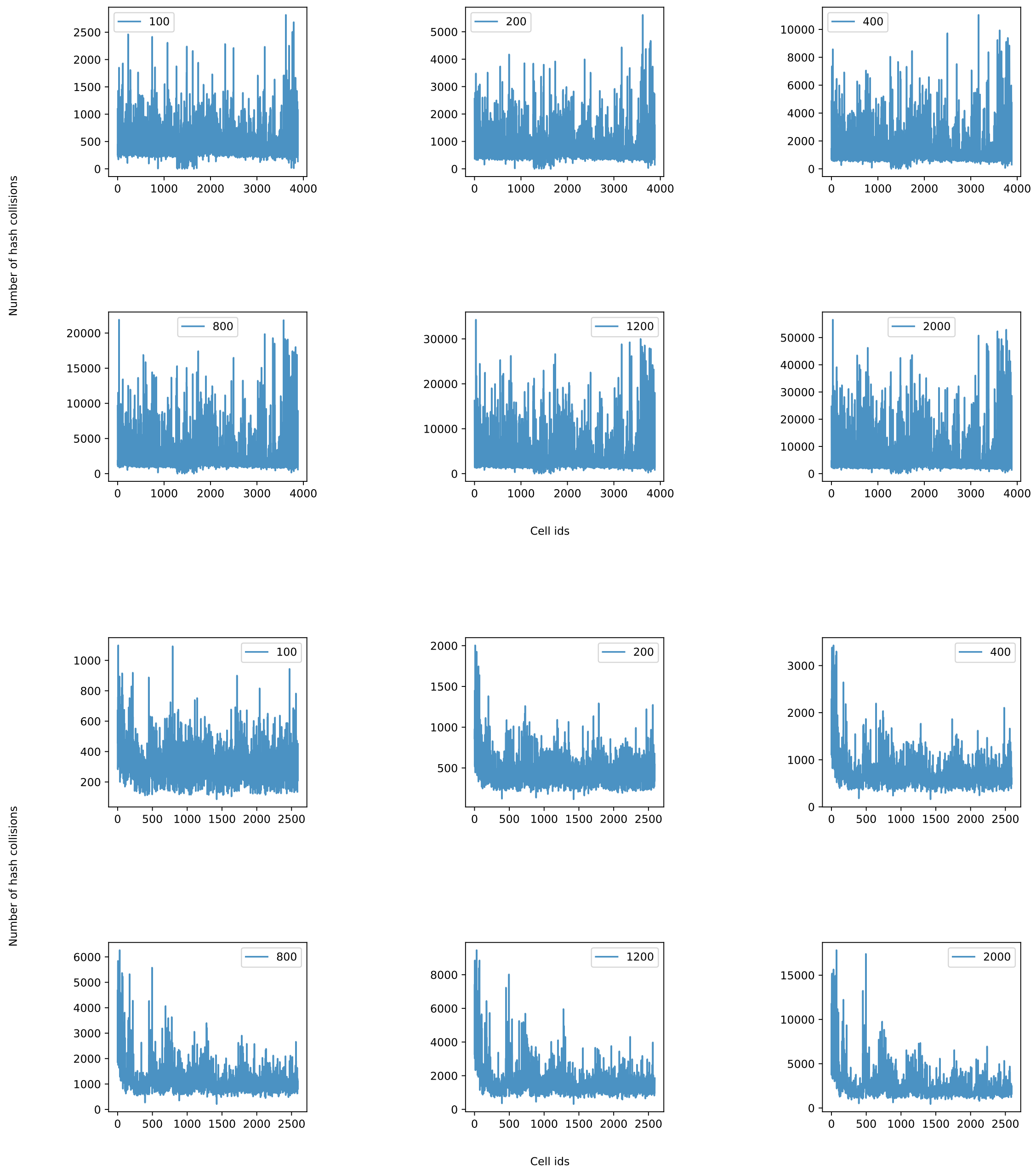

Figure S 9: Number of hash collisions per cell for 1Mb (top) and 10kb (bottom) cells from Nagano *et al.* (2017). The number of hash collisions is shown for 100, 200, 400, 800, 1200 and 2000 hash functions. One collision occurs if two cells have for one hash function the same hash value.

3.2 Collision occurrences

The collision occurrences statistics maps the number of collisions for a hash value of a hash function (x-axis) with the occurrences of the number of collisions' overall hash functions and hash values.

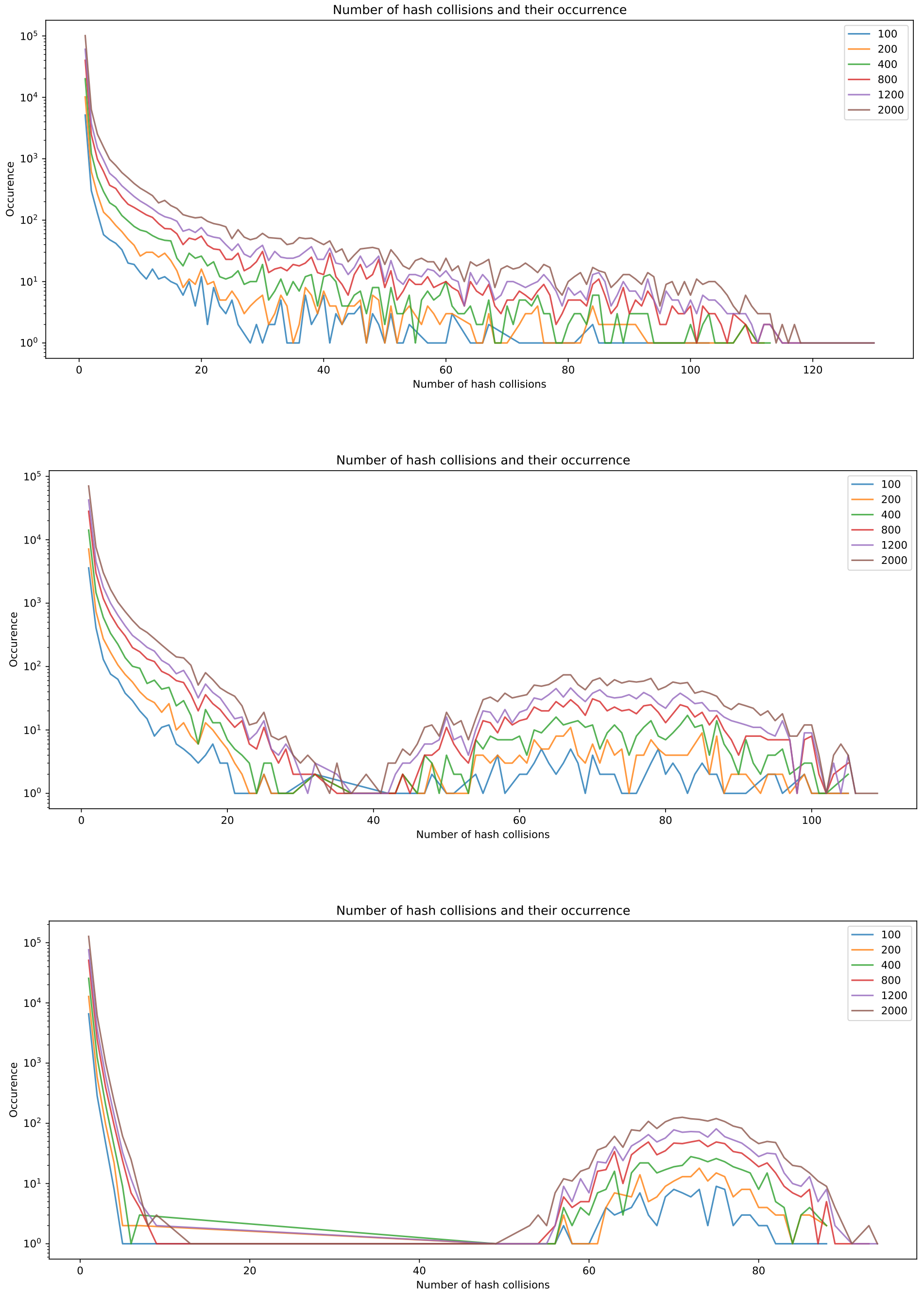

Figure S 10: Size of hash collisions (x-axis) and their collision occurrences (y-axis) for 100kb (top), 10kb (middle) and 1kb (bottom) for 144 cells from Gassler *et al.* (2017). The number of hash collisions is shown for 100, 200, 400, 800, 1200 and 2000 hash functions.

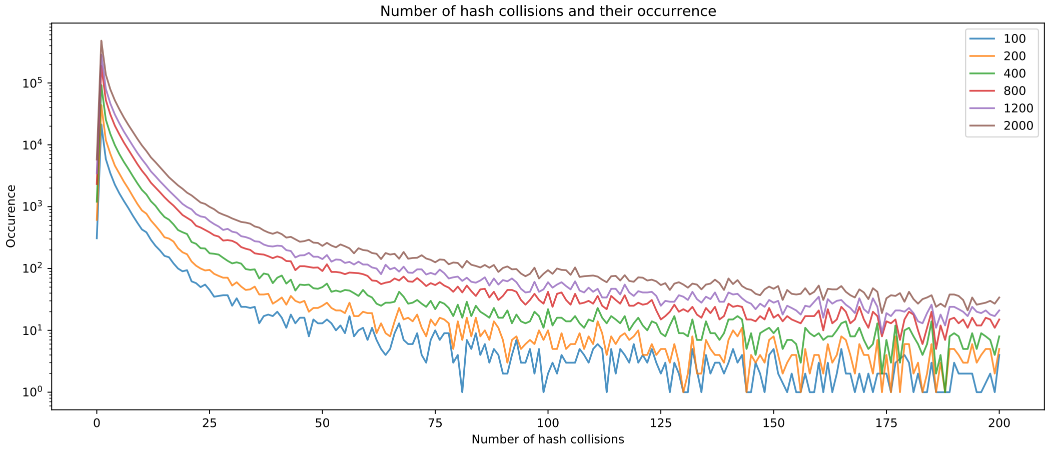

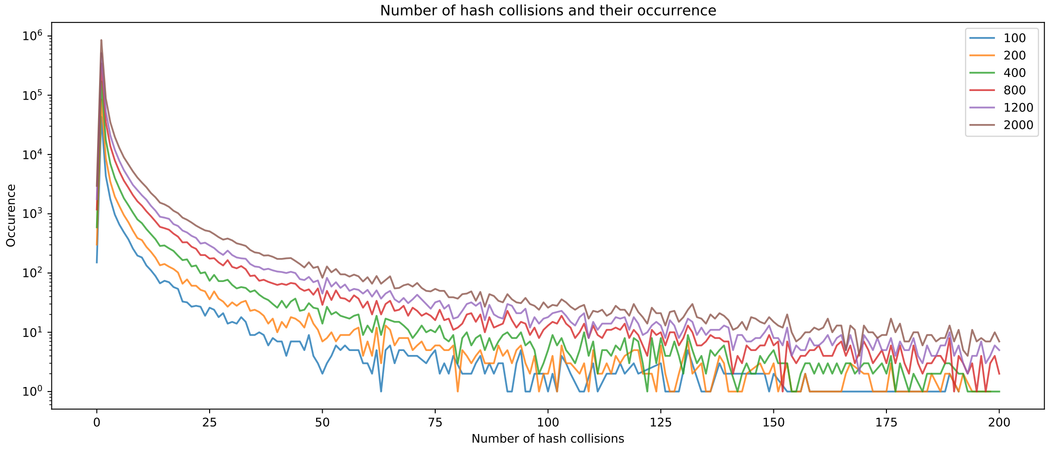

Figure S 11: Size of hash collisions (x-axis) and their collision occurrences (y-axis)for 1Mb (top) and 10kb (bottom) for 2472 cells from Nagano *et al.* (2017). The number of hash collisions is shown for 100, 200, 400, 800, 1200 and 2000 hash functions.

3.3 Number of hash values per hash function

The here shown collision statistics are for different interaction matrix resolutions from Nagano *et al.* (2017) and Gassler *et al.* (2017).

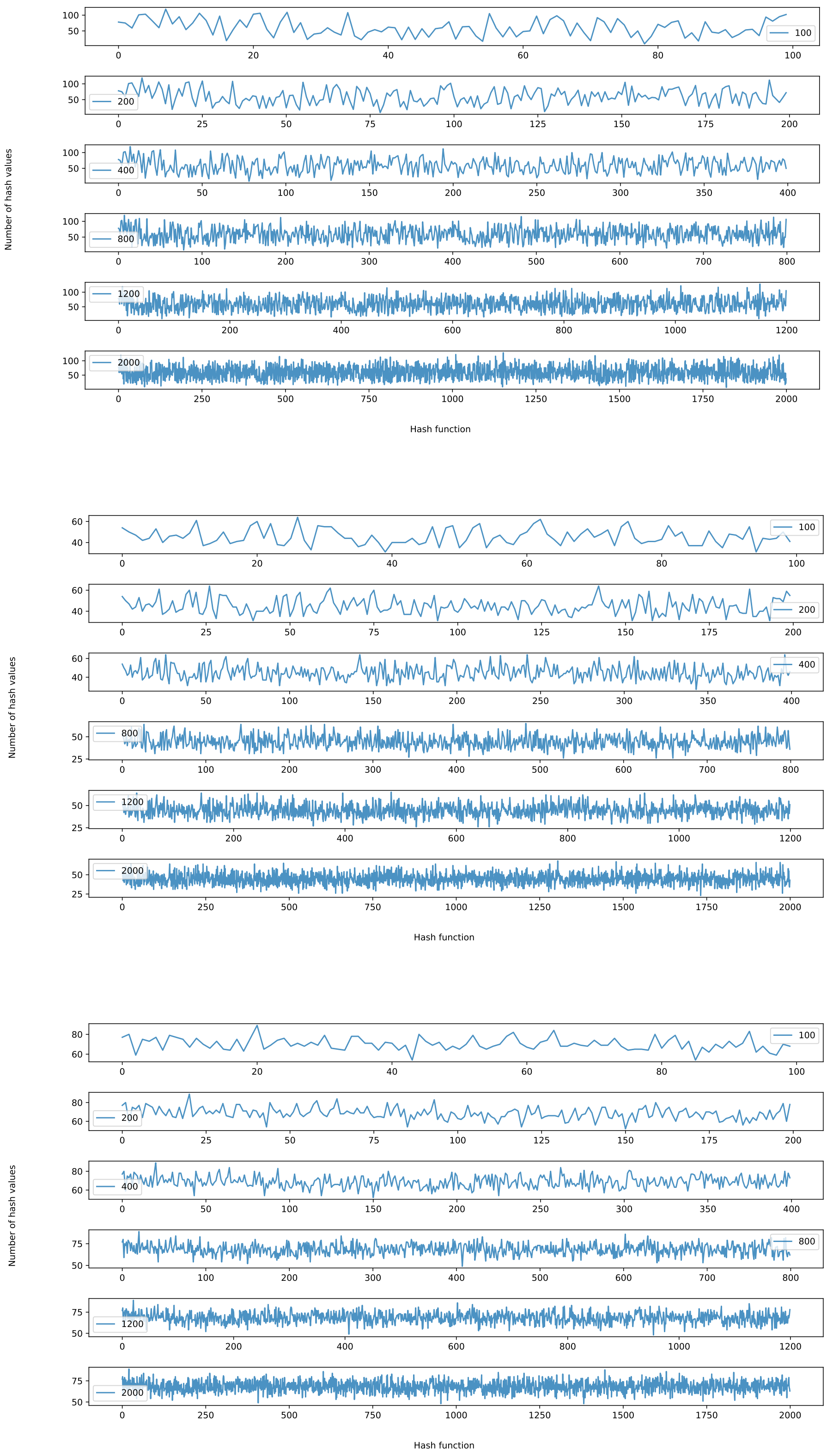

Figure S 12: Number of hash values per hash function for 100kb (top), 10kb (middle) and 1kb (bottom) for 144 cells from Gassler *et al.* (2017). The number of hash values per hash function is shown for 100, 200, 400, 800, 1200 and 2000 hash functions.

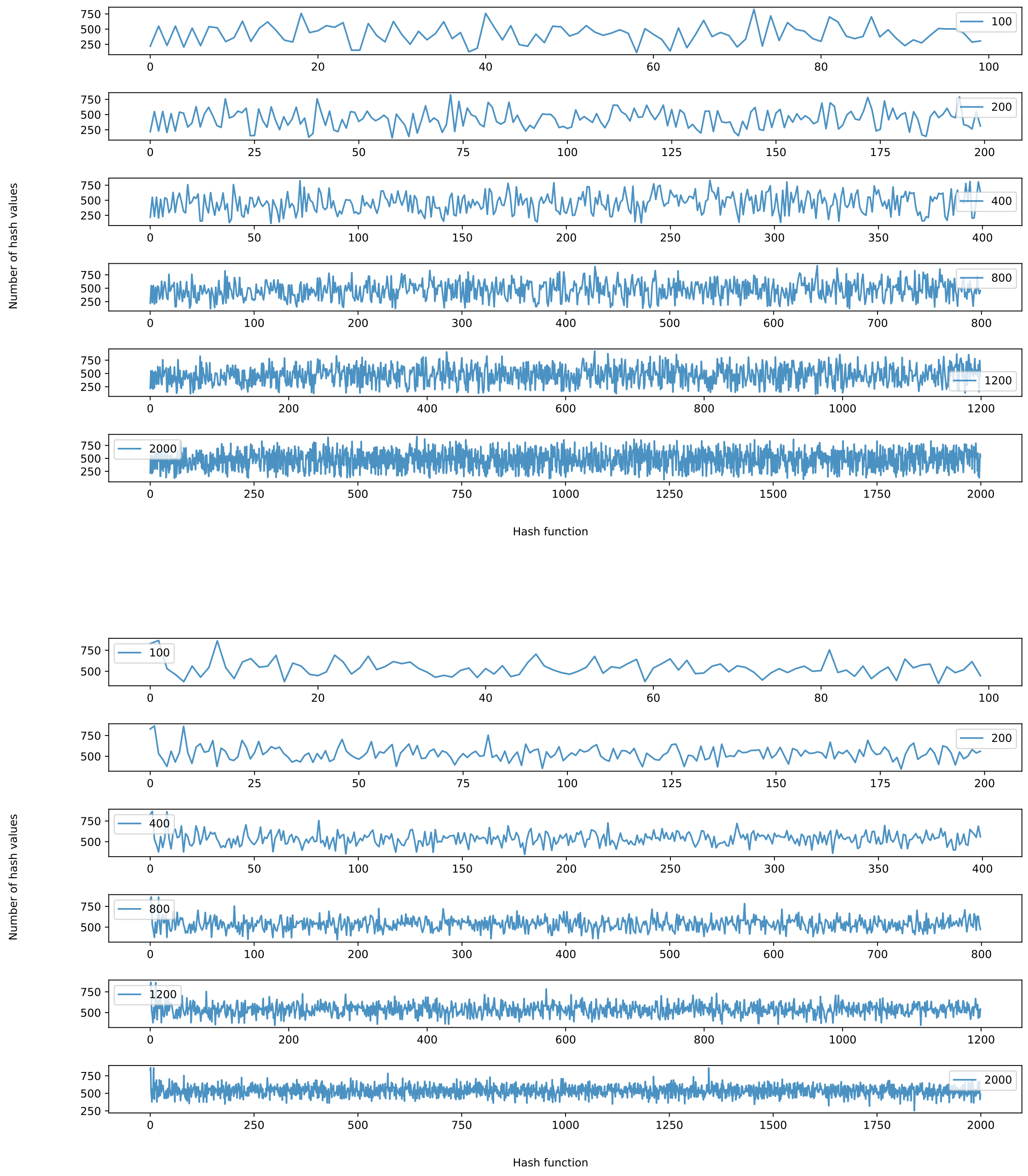

Figure S 13: Number of hash values per hash function for 1Mb (top) and 10kb (bottom) for 2472 cells from Nagano *et al.* (2017). The number of hash collisions is shown for 100, 200, 400, 800, 1200 and 2000 hash functions. One collision occurs if two cells have for one hash function the same hash value.

4 Cluster results

4.1 Cluster overlaps

Percentage values define the number of cells of a cluster which are associated with a specific cell cycle phase, i.e., in Table 1 cluster 1 has 166 cells, and 2 are associated with cell cycle stage G1; therefore, 2 / 166 or 1.2% of cluster 1 is from cell cycle stage G1. Correct identified: This measures how many percent of a cluster are unique identified with a cell phase or cell type. For example, 155 cells out of 300 G1 cells are unique, with a level of at least 80 % in their clusters. These are Cluster 2 with 84.1%, Cluster 4 with 95.8%, and 7 with each 97.9%.

4.1.1 Nagano 1MB

Cluster	G1 (300 cells)	early-S (573 cells)	late-S/G2 (362 cells)	post-M (17 cells)	pre-M (23 cells)
Cluster 0 (25 cells)	0 cells / 0.00 %	0 cells / 0.00 %	4 cells / 16.00 %	0 cells / 0.00 %	21 cells / 84.00 %
Cluster 1 (166 cells)	2 cells / 1.20 %	147 cells / 88.55 %	17 cells / 10.24 %	0 cells / 0.00 %	0 cells / 0.00 %
Cluster 2 (101 cells)	85 cells / 84.16 %	16 cells / 15.84 %	0 cells / 0.00 %	0 cells / 0.00 %	0 cells / 0.00 %
Cluster 3 (111 cells)	0 cells / 0.00 %	10 cells / 9.01 %	100 cells / 90.09 %	0 cells / 0.00 %	1 cell / 0.90 %
Cluster 4 (24 cells)	23 cells / 95.83 %	0 cells / 0.00 %	0 cells / 0.00 %	1 cell / 4.17 %	0 cells / 0.00 %
Cluster 5 (103 cells)	0 cells / 0.00 %	12 cells / 11.65 %	91 cells / 88.35 %	0 cells / 0.00 %	0 cells / 0.00 %
Cluster 6 (85 cells)	66 cells / 77.65 %	19 cells / 22.35 %	0 cells / 0.00 %	0 cells / 0.00 %	0 cells / 0.00 %
Cluster 7 (48 cells)	47 cells / 97.92 %	1 cell / 2.08 %	0 cells / 0.00 %	0 cells / 0.00 %	0 cells / 0.00 %
Cluster 8 (239 cells)	15 cells / 6.28 %	202 cells / 84.52 %	22 cells / 9.21 %	0 cells / 0.00 %	0 cells / 0.00 %
Cluster 9 (19 cells)	2 cells / 10.53 %	0 cells / 0.00 %	0 cells / 0.00 %	16 cells / 84.21 %	1 cell / 5.26 %
Cluster 10 (203 cells)	60 cells / 29.56 %	141 cells / 69.46 %	2 cells / 0.99 %	0 cells / 0.00 %	0 cells / 0.00 %
Cluster 11 (151 cells)	0 cells / 0.00 %	25 cells / 16.56 %	126 cells / 83.44 %	0 cells / 0.00 %	0 cells / 0.00 %
Correct identified > 70%	221 / 300 (73.67 %)	349 / 573 (60.91 %)	317 / 362 (87.57 %)	16 / 17 (94.12 %)	21 / 23 (91.30 %)
Correct identified > 80%	155 / 300 (51.67 %)	349 / 573 (60.91 %)	317 / 362 (87.57 %)	16 / 17 (94.12 %)	21 / 23 (91.30 %)
Correct identified > 90%	70 / 300 (23.33 %)	0 / 573 (0.00 %)	100 / 362 (27.62 %)	0 / 17 (0.00 %)	0 / 23 (0.00 %)

Table 1: Overlaps of detect clusters with known cell cycle stages from Nagano *et al.* (2017). Clustering with approximate k-nearest neighbors, 28000 hash functions, 55 principal components and UMAP k-neighbors 58, UMAP components 5 and UMAP min distance 0.2886.

Cluster	G1 (300 cells)	early-S (573 cells)	late-S/G2 (362 cells)	post-M (17 cells)	pre-M (23 cells)
Cluster 0 (95 cells)	0 cells / 0.00 %	1 cell / 1.05 %	94 cells / 98.95 %	0 cells / 0.00 %	0 cells / 0.00 %
Cluster 1 (158 cells)	33 cells / 20.89 %	124 cells / 78.48 %	1 cell / 0.63 %	0 cells / 0.00 %	0 cells / 0.00 %
Cluster 2 (88 cells)	72 cells / 81.82 %	16 cells / 18.18 %	0 cells / 0.00 %	0 cells / 0.00 %	0 cells / 0.00 %
Cluster 3 (125 cells)	5 cells / 4.00 %	31 cells / 24.80 %	82 cells / 65.60 %	0 cells / 0.00 %	7 cells / 5.60 %
Cluster 4 (104 cells)	13 cells / 12.50 %	89 cells / 85.58 %	2 cells / 1.92 %	0 cells / 0.00 %	0 cells / 0.00 %
Cluster 5 (117 cells)	70 cells / 59.83 %	47 cells / 40.17 %	0 cells / 0.00 %	0 cells / 0.00 %	0 cells / 0.00 %
Cluster 6 (94 cells)	0 cells / 0.00 %	39 cells / 41.49 %	55 cells / 58.51 %	0 cells / 0.00 %	0 cells / 0.00 %
Cluster 7 (91 cells)	84 cells / 92.31 %	7 cells / 7.69 %	0 cells / 0.00 %	0 cells / 0.00 %	0 cells / 0.00 %
Cluster 8 (53 cells)	21 cells / 39.62 %	0 cells / 0.00 %	0 cells / 0.00 %	17 cells / 32.08 %	15 cells / 28.30 %
Cluster 9 (99 cells)	1 cell / 1.01 %	94 cells / 94.95 %	4 cells / 4.04 %	0 cells / 0.00 %	0 cells / 0.00 %
Cluster 10 (158 cells)	1 cell / 0.63 %	110 cells / 69.62 %	46 cells / 29.11 %	0 cells / 0.00 %	1 cell / 0.63 %
Cluster 11 (93 cells)	0 cells / 0.00 %	15 cells / 16.13 %	78 cells / 83.87 %	0 cells / 0.00 %	0 cells / 0.00 %
Correct identified > 70%	156 / 300 (52.00 %)	307 / 573 (53.58 %)	172 / 362 (47.51 %)	0 / 17 (0.00 %)	0 / 23 (0.00 %)
Correct identified > 80%	156 / 300 (52.00 %)	183 / 573 (31.94 %)	172 / 362 (47.51 %)	0 / 17 (0.00 %)	0 / 23 (0.00 %)
Correct identified > 90%	84 / 300 (28.00 %)	94 / 573 (16.40 %)	94 / 362 (25.97 %)	0 / 17 (0.00 %)	0 / 23 (0.00 %)

Table 2: Overlaps of detect clusters with known cell cycle stages from Nagano *et al.* (2017). Clustering with approximate k-nearest neighbors, 28000 hash functions, 55 principal components and UMAP k-neighbors 58, UMAP components 2 and UMAP min distance 0.2886.

Cluster	G1 (300 cells)	early-S (573 cells)	late-S/G2 (362 cells)	post-M (17 cells)	pre-M (23 cells)
Cluster 0 (153 cells)	113 cells / 73.86 %	27 cells / 17.65 %	13 cells / 8.50 %	0 cells / 0.00 %	0 cells / 0.00 %
Cluster 1 (151 cells)	39 cells / 25.83 %	100 cells / 66.23 %	12 cells / 7.95 %	0 cells / 0.00 %	0 cells / 0.00 %
Cluster 2 (70 cells)	0 cells / 0.00 %	67 cells / 95.71 %	3 cells / 4.29 %	0 cells / 0.00 %	0 cells / 0.00 %
Cluster 3 (114 cells)	4 cells / 3.51 %	17 cells / 14.91 %	84 cells / 73.68 %	3 cells / 2.63 %	6 cells / 5.26 %
Cluster 4 (96 cells)	4 cells / 4.17 %	24 cells / 25.00 %	66 cells / 68.75 %	1 cell / 1.04 %	1 cell / 1.04 %
Cluster 5 (93 cells)	2 cells / 2.15 %	23 cells / 24.73 %	57 cells / 61.29 %	3 cells / 3.23 %	8 cells / 8.60 %
Cluster 6 (76 cells)	24 cells / 31.58 %	49 cells / 64.47 %	3 cells / 3.95 %	0 cells / 0.00 %	0 cells / 0.00 %
Cluster 7 (131 cells)	85 cells / 64.89 %	43 cells / 32.82 %	3 cells / 2.29 %	0 cells / 0.00 %	0 cells / 0.00 %
Cluster 8 (152 cells)	8 cells / 5.26 %	138 cells / 90.79 %	6 cells / 3.95 %	0 cells / 0.00 %	0 cells / 0.00 %
Cluster 9 (72 cells)	21 cells / 29.17 %	44 cells / 61.11 %	7 cells / 9.72 %	0 cells / 0.00 %	0 cells / 0.00 %
Cluster 10 (42 cells)	0 cells / 0.00 %	5 cells / 11.90 %	33 cells / 78.57 %	1 cell / 2.38 %	3 cells / 7.14 %
Cluster 11 (125 cells)	0 cells / 0.00 %	36 cells / 28.80 %	75 cells / 60.00 %	9 cells / 7.20 %	5 cells / 4.00 %
Correct identified > 70%	113 / 300 (37.67 %)	205 / 573 (35.78 %)	117 / 362 (32.32 %)	0 / 17 (0.00 %)	0 / 23 (0.00 %)
Correct identified > 80%	0 / 300 (0.00 %)	205 / 573 (35.78 %)	0 / 362 (0.00 %)	0 / 17 (0.00 %)	0 / 23 (0.00 %)
Correct identified > 90%	0 / 300 (0.00 %)	205 / 573 (35.78 %)	0 / 362 (0.00 %)	0 / 17 (0.00 %)	0 / 23 (0.00 %)

Table 3: Approximate nearest neighbors with MinHash to preselect a candidate set. On the candidate set the nearest neighbors for a cell are computed with the Euclidean distance.

Cluster	G1 (300 cells)	early-S (573 cells)	late-S/G2 (362 cells)	post-M (17 cells)	pre-M (23 cells)
Cluster 0 (36 cells)	0 cells / 0.00 %	12 cells / 33.33 %	24 cells / 66.67 %	0 cells / 0.00 %	0 cells / 0.00 %
Cluster 1 (199 cells)	0 cells / 0.00 %	164 cells / 82.41 %	35 cells / 17.59 %	0 cells / 0.00 %	0 cells / 0.00 %
Cluster 2 (130 cells)	0 cells / 0.00 %	117 cells / 90.00 %	13 cells / 10.00 %	0 cells / 0.00 %	0 cells / 0.00 %
Cluster 3 (44 cells)	23 cells / 52.27 %	21 cells / 47.73 %	0 cells / 0.00 %	0 cells / 0.00 %	0 cells / 0.00 %
Cluster 4 (62 cells)	0 cells / 0.00 %	3 cells / 4.84 %	41 cells / 66.13 %	0 cells / 0.00 %	18 cells / 29.03 %
Cluster 5 (258 cells)	0 cells / 0.00 %	75 cells / 29.07 %	183 cells / 70.93 %	0 cells / 0.00 %	0 cells / 0.00 %
Cluster 6 (129 cells)	99 cells / 76.74 %	30 cells / 23.26 %	0 cells / 0.00 %	0 cells / 0.00 %	0 cells / 0.00 %
Cluster 7 (48 cells)	46 cells / 95.83 %	1 cell / 2.08 %	0 cells / 0.00 %	1 cell / 2.08 %	0 cells / 0.00 %
Cluster 8 (193 cells)	55 cells / 28.50 %	137 cells / 70.98 %	1 cell / 0.52 %	0 cells / 0.00 %	0 cells / 0.00 %
Cluster 9 (70 cells)	0 cells / 0.00 %	5 cells / 7.14 %	65 cells / 92.86 %	0 cells / 0.00 %	0 cells / 0.00 %
Cluster 10 (21 cells)	0 cells / 0.00 %	0 cells / 0.00 %	0 cells / 0.00 %	16 cells / 76.19 %	5 cells / 23.81 %
Cluster 11 (85 cells)	77 cells / 90.59 %	8 cells / 9.41 %	0 cells / 0.00 %	0 cells / 0.00 %	0 cells / 0.00 %
Correct identified > 70%	222 / 300 (74.00 %)	418 / 573 (72.95 %)	248 / 362 (68.51 %)	16 / 17 (94.12 %)	0 / 23 (0.00 %)
Correct identified > 80%	123 / 300 (41.00 %)	281 / 573 (49.04 %)	65 / 362 (17.96 %)	0 / 17 (0.00 %)	0 / 23 (0.00 %)
Correct identified > 90%	123 / 300 (41.00 %)	117 / 573 (20.42 %)	65 / 362 (17.96 %)	0 / 17 (0.00 %)	0 / 23 (0.00 %)

Table 4: After the approximate nearest neighbors graph a principal component analysis but no UMAP embedding is computed before the data is clustered.

Cluster	G1 (300 cells)	early-S (573 cells)	late-S/G2 (362 cells)	post-M (17 cells)	pre-M (23 cells)
Cluster 0 (30 cells)	14 cells / 46.67 %	16 cells / 53.33 %	0 cells / 0.00 %	0 cells / 0.00 %	0 cells / 0.00 %
Cluster 1 (177 cells)	0 cells / 0.00 %	75 cells / 42.37 %	102 cells / 57.63 %	0 cells / 0.00 %	0 cells / 0.00 %
Cluster 2 (105 cells)	0 cells / 0.00 %	19 cells / 18.10 %	86 cells / 81.90 %	0 cells / 0.00 %	0 cells / 0.00 %
Cluster 3 (191 cells)	47 cells / 24.61 %	143 cells / 74.87 %	1 cell / 0.52 %	0 cells / 0.00 %	0 cells / 0.00 %
Cluster 4 (162 cells)	0 cells / 0.00 %	24 cells / 14.81 %	120 cells / 74.07 %	0 cells / 0.00 %	18 cells / 11.11 %
Cluster 5 (94 cells)	85 cells / 90.43 %	9 cells / 9.57 %	0 cells / 0.00 %	0 cells / 0.00 %	0 cells / 0.00 %
Cluster 6 (68 cells)	51 cells / 75.00 %	17 cells / 25.00 %	0 cells / 0.00 %	0 cells / 0.00 %	0 cells / 0.00 %
Cluster 7 (81 cells)	0 cells / 0.00 %	61 cells / 75.31 %	20 cells / 24.69 %	0 cells / 0.00 %	0 cells / 0.00 %
Cluster 8 (112 cells)	92 cells / 82.14 %	20 cells / 17.86 %	0 cells / 0.00 %	0 cells / 0.00 %	0 cells / 0.00 %
Cluster 9 (92 cells)	1 cell / 1.09 %	85 cells / 92.39 %	6 cells / 6.52 %	0 cells / 0.00 %	0 cells / 0.00 %
Cluster 10 (24 cells)	10 cells / 41.67 %	0 cells / 0.00 %	0 cells / 0.00 %	14 cells / 58.33 %	0 cells / 0.00 %
Cluster 11 (139 cells)	0 cells / 0.00 %	104 cells / 74.82 %	27 cells / 19.42 %	3 cells / 2.16 %	5 cells / 3.60 %
Correct identified > 70%	228 / 300 (76.00 %)	393 / 573 (68.59 %)	206 / 362 (56.91 %)	0 / 17 (0.00 %)	0 / 23 (0.00 %)
Correct identified > 80%	177 / 300 (59.00 %)	85 / 573 (14.83 %)	86 / 362 (23.76 %)	0 / 17 (0.00 %)	0 / 23 (0.00 %)
Correct identified > 90%	85 / 300 (28.33 %)	85 / 573 (14.83 %)	0 / 362 (0.00 %)	0 / 17 (0.00 %)	0 / 23 (0.00 %)

Table 5: After the approximate nearest neighbors graph no principal component analysis but an UMAP embedding is computed before the data is clustered.

Cluster	G1 (300 cells)	early-S (573 cells)	late-S/G2 (362 cells)	post-M (17 cells)	pre-M (23 cells)
Cluster 0 (104 cells)	86 cells / 82.69 %	18 cells / 17.31 %	0 cells / 0.00 %	0 cells / 0.00 %	0 cells / 0.00 %
Cluster 1 (187 cells)	1 cell / 0.53 %	155 cells / 82.89 %	31 cells / 16.58 %	0 cells / 0.00 %	0 cells / 0.00 %
Cluster 2 (67 cells)	0 cells / 0.00 %	4 cells / 5.97 %	46 cells / 68.66 %	0 cells / 0.00 %	17 cells / 25.37 %
Cluster 3 (143 cells)	1 cell / 0.70 %	124 cells / 86.71 %	18 cells / 12.59 %	0 cells / 0.00 %	0 cells / 0.00 %
Cluster 4 (248 cells)	0 cells / 0.00 %	75 cells / 30.24 %	173 cells / 69.76 %	0 cells / 0.00 %	0 cells / 0.00 %
Cluster 5 (85 cells)	60 cells / 70.59 %	25 cells / 29.41 %	0 cells / 0.00 %	0 cells / 0.00 %	0 cells / 0.00 %
Cluster 6 (48 cells)	46 cells / 95.83 %	1 cell / 2.08 %	0 cells / 0.00 %	1 cell / 2.08 %	0 cells / 0.00 %
Cluster 7 (41 cells)	23 cells / 56.10 %	18 cells / 43.90 %	0 cells / 0.00 %	0 cells / 0.00 %	0 cells / 0.00 %
Cluster 8 (42 cells)	37 cells / 88.10 %	5 cells / 11.90 %	0 cells / 0.00 %	0 cells / 0.00 %	0 cells / 0.00 %
Cluster 9 (24 cells)	2 cells / 8.33 %	0 cells / 0.00 %	0 cells / 0.00 %	16 cells / 66.67 %	6 cells / 25.00 %
Cluster 10 (120 cells)	0 cells / 0.00 %	27 cells / 22.50 %	93 cells / 77.50 %	0 cells / 0.00 %	0 cells / 0.00 %
Cluster 11 (166 cells)	44 cells / 26.51 %	121 cells / 72.89 %	1 cell / 0.60 %	0 cells / 0.00 %	0 cells / 0.00 %
Correct identified > 70%	229 / 300 (76.33 %)	400 / 573 (69.81 %)	93 / 362 (25.69 %)	0 / 17 (0.00 %)	0 / 23 (0.00 %)
Correct identified > 80%	169 / 300 (56.33 %)	279 / 573 (48.69 %)	0 / 362 (0.00 %)	0 / 17 (0.00 %)	0 / 23 (0.00 %)
Correct identified > 90%	46 / 300 (15.33 %)	0 / 573 (0.00 %)	0 / 362 (0.00 %)	0 / 17 (0.00 %)	0 / 23 (0.00 %)

Table 6: After the approximate nearest neighbors graph no principal component analysis and no UMAP embedding is computed before the data is clustered.

4.2 Intra- and inter-chromosomal contacts

Cluster	G1 (300 cells)	early-S (573 cells)	late-S/G2 (362 cells)	post-M (17 cells)	pre-M (23 cells)
Cluster 0 (177 cells)	22 cells / 12.43 %	100 cells / 56.50 %	55 cells / 31.07 %	0 cells / 0.00 %	0 cells / 0.00 %
Cluster 1 (205 cells)	14 cells / 6.83 %	127 cells / 61.95 %	64 cells / 31.22 %	0 cells / 0.00 %	0 cells / 0.00 %
Cluster 2 (169 cells)	109 cells / 64.50 %	60 cells / 35.50 %	0 cells / 0.00 %	0 cells / 0.00 %	0 cells / 0.00 %
Cluster 3 (83 cells)	0 cells / 0.00 %	17 cells / 20.48 %	64 cells / 77.11 %	0 cells / 0.00 %	2 cells / 2.41 %
Cluster 4 (141 cells)	0 cells / 0.00 %	9 cells / 6.38 %	132 cells / 93.62 %	0 cells / 0.00 %	0 cells / 0.00 %
Cluster 5 (16 cells)	1 cell / 6.25 %	0 cells / 0.00 %	0 cells / 0.00 %	15 cells / 93.75 %	0 cells / 0.00 %
Cluster 6 (220 cells)	4 cells / 1.82 %	174 cells / 79.09 %	42 cells / 19.09 %	0 cells / 0.00 %	0 cells / 0.00 %
Cluster 7 (30 cells)	29 cells / 96.67 %	0 cells / 0.00 %	0 cells / 0.00 %	1 cell / 3.33 %	0 cells / 0.00 %
Cluster 8 (55 cells)	51 cells / 92.73 %	1 cell / 1.82 %	1 cell / 1.82 %	0 cells / 0.00 %	2 cells / 3.64 %
Cluster 9 (102 cells)	42 cells / 41.18 %	60 cells / 58.82 %	0 cells / 0.00 %	0 cells / 0.00 %	0 cells / 0.00 %
Cluster 10 (22 cells)	0 cells / 0.00 %	0 cells / 0.00 %	2 cells / 9.09 %	1 cell / 4.55 %	19 cells / 86.36 %
Cluster 11 (55 cells)	28 cells / 50.91 %	25 cells / 45.45 %	2 cells / 3.64 %	0 cells / 0.00 %	0 cells / 0.00 %
Correct identified > 70%	80 / 300 (26.67 %)	174 / 573 (30.37 %)	196 / 362 (54.14 %)	15 / 17 (88.24 %)	19 / 23 (82.61 %)
Correct identified > 80%	80 / 300 (26.67 %)	0 / 573 (0.00 %)	132 / 362 (36.46 %)	15 / 17 (88.24 %)	19 / 23 (82.61 %)
Correct identified > 90%	80 / 300 (26.67 %)	0 / 573 (0.00 %)	132 / 362 (36.46 %)	15 / 17 (88.24 %)	0 / 23 (0.00 %)

Table 7: Computing the approximate nearest neighbors graph with all Hi-C contacts: intra- and inter-chromosomal contacts.

4.3 Number of hash functions

Cluster	G1 (300 cells)	early-S (573 cells)	late-S/G2 (362 cells)	post-M (17 cells)	pre-M (23 cells)
Cluster 0 (144 cells)	4 cells / 2.78 %	70 cells / 48.61 %	69 cells / 47.92 %	1 cell / 0.69 %	0 cells / 0.00 %
Cluster 1 (89 cells)	48 cells / 53.93 %	39 cells / 43.82 %	1 cell / 1.12 %	1 cell / 1.12 %	0 cells / 0.00 %
Cluster 2 (164 cells)	7 cells / 4.27 %	119 cells / 72.56 %	37 cells / 22.56 %	0 cells / 0.00 %	1 cell / 0.61 %
Cluster 3 (183 cells)	44 cells / 24.04 %	99 cells / 54.10 %	39 cells / 21.31 %	0 cells / 0.00 %	1 cell / 0.55 %
Cluster 4 (10 cells)	0 cells / 0.00 %	0 cells / 0.00 %	0 cells / 0.00 %	7 cells / 70.00 %	3 cells / 30.00 %
Cluster 5 (67 cells)	56 cells / 83.58 %	5 cells / 7.46 %	3 cells / 4.48 %	0 cells / 0.00 %	3 cells / 4.48 %
Cluster 6 (96 cells)	71 cells / 73.96 %	22 cells / 22.92 %	2 cells / 2.08 %	0 cells / 0.00 %	1 cell / 1.04 %
Cluster 7 (10 cells)	2 cells / 20.00 %	0 cells / 0.00 %	0 cells / 0.00 %	8 cells / 80.00 %	0 cells / 0.00 %
Cluster 8 (151 cells)	43 cells / 28.48 %	100 cells / 66.23 %	8 cells / 5.30 %	0 cells / 0.00 %	0 cells / 0.00 %
Cluster 9 (13 cells)	1 cell / 7.69 %	0 cells / 0.00 %	2 cells / 15.38 %	0 cells / 0.00 %	10 cells / 76.92 %
Cluster 10 (182 cells)	15 cells / 8.24 %	77 cells / 42.31 %	88 cells / 48.35 %	0 cells / 0.00 %	2 cells / 1.10 %
Cluster 11 (166 cells)	9 cells / 5.42 %	42 cells / 25.30 %	113 cells / 68.07 %	0 cells / 0.00 %	2 cells / 1.20 %
Correct identified > 70%	127 / 300 (42.33 %)	119 / 573 (20.77 %)	0 / 362 (0.00 %)	15 / 17 (88.24 %)	10 / 23 (43.48 %)
Correct identified > 80%	56 / 300 (18.67 %)	0 / 573 (0.00 %)	0 / 362 (0.00 %)	8 / 17 (47.06 %)	0 / 23 (0.00 %)
Correct identified > 90%	0 / 300 (0.00 %)	0 / 573 (0.00 %)	0 / 362 (0.00 %)	0 / 17 (0.00 %)	0 / 23 (0.00 %)

Table 8: 2000 hash functions.

Cluster	G1 (300 cells)	early-S (573 cells)	late-S/G2 (362 cells)	post-M (17 cells)	pre-M (23 cells)
Cluster 0 (125 cells)	3 cells / 2.40 %	101 cells / 80.80 %	21 cells / 16.80 %	0 cells / 0.00 %	0 cells / 0.00 %
Cluster 1 (153 cells)	58 cells / 37.91 %	93 cells / 60.78 %	2 cells / 1.31 %	0 cells / 0.00 %	0 cells / 0.00 %
Cluster 2 (163 cells)	1 cell / 0.61 %	14 cells / 8.59 %	147 cells / 90.18 %	0 cells / 0.00 %	1 cell / 0.61 %
Cluster 3 (189 cells)	3 cells / 1.59 %	98 cells / 51.85 %	88 cells / 46.56 %	0 cells / 0.00 %	0 cells / 0.00 %
Cluster 4 (66 cells)	65 cells / 98.48 %	0 cells / 0.00 %	1 cell / 1.52 %	0 cells / 0.00 %	0 cells / 0.00 %
Cluster 5 (76 cells)	0 cells / 0.00 %	6 cells / 7.89 %	69 cells / 90.79 %	0 cells / 0.00 %	1 cell / 1.32 %
Cluster 6 (22 cells)	3 cells / 13.64 %	0 cells / 0.00 %	0 cells / 0.00 %	16 cells / 72.73 %	3 cells / 13.64 %
Cluster 7 (17 cells)	0 cells / 0.00 %	0 cells / 0.00 %	0 cells / 0.00 %	0 cells / 0.00 %	17 cells / 100.00 %
Cluster 8 (106 cells)	61 cells / 57.55 %	44 cells / 41.51 %	1 cell / 0.94 %	0 cells / 0.00 %	0 cells / 0.00 %
Cluster 9 (23 cells)	22 cells / 95.65 %	0 cells / 0.00 %	0 cells / 0.00 %	1 cell / 4.35 %	0 cells / 0.00 %
Cluster 10 (147 cells)	78 cells / 53.06 %	68 cells / 46.26 %	1 cell / 0.68 %	0 cells / 0.00 %	0 cells / 0.00 %
Cluster 11 (188 cells)	6 cells / 3.19 %	149 cells / 79.26 %	32 cells / 17.02 %	0 cells / 0.00 %	1 cell / 0.53 %
Correct identified > 70%	87 / 300 (29.00 %)	250 / 573 (43.63 %)	216 / 362 (59.67 %)	16 / 17 (94.12 %)	17 / 23 (73.91 %)
Correct identified > 80%	87 / 300 (29.00 %)	101 / 573 (17.63 %)	216 / 362 (59.67 %)	0 / 17 (0.00 %)	17 / 23 (73.91 %)
Correct identified > 90%	87 / 300 (29.00 %)	0 / 573 (0.00 %)	216 / 362 (59.67 %)	0 / 17 (0.00 %)	17 / 23 (73.91 %)

Table 9: 10,000 hash functions.

4.4 K-neighbors

Cluster	G1 (300 cells)	early-S (573 cells)	late-S/G2 (362 cells)	post-M (17 cells)	pre-M (23 cells)
Cluster 0 (103 cells)	10 cells / 9.71 %	82 cells / 79.61 %	11 cells / 10.68 %	0 cells / 0.00 %	0 cells / 0.00 %
Cluster 1 (177 cells)	16 cells / 9.04 %	115 cells / 64.97 %	46 cells / 25.99 %	0 cells / 0.00 %	0 cells / 0.00 %
Cluster 2 (183 cells)	17 cells / 9.29 %	97 cells / 53.01 %	69 cells / 37.70 %	0 cells / 0.00 %	0 cells / 0.00 %
Cluster 3 (193 cells)	40 cells / 20.73 %	126 cells / 65.28 %	27 cells / 13.99 %	0 cells / 0.00 %	0 cells / 0.00 %
Cluster 4 (83 cells)	0 cells / 0.00 %	5 cells / 6.02 %	72 cells / 86.75 %	0 cells / 0.00 %	6 cells / 7.23 %
Cluster 5 (113 cells)	98 cells / 86.73 %	10 cells / 8.85 %	3 cells / 2.65 %	0 cells / 0.00 %	2 cells / 1.77 %
Cluster 6 (116 cells)	0 cells / 0.00 %	18 cells / 15.52 %	96 cells / 82.76 %	0 cells / 0.00 %	2 cells / 1.72 %
Cluster 7 (46 cells)	10 cells / 21.74 %	3 cells / 6.52 %	3 cells / 6.52 %	17 cells / 36.96 %	13 cells / 28.26 %
Cluster 8 (83 cells)	50 cells / 60.24 %	33 cells / 39.76 %	0 cells / 0.00 %	0 cells / 0.00 %	0 cells / 0.00 %
Cluster 9 (18 cells)	0 cells / 0.00 %	8 cells / 44.44 %	10 cells / 55.56 %	0 cells / 0.00 %	0 cells / 0.00 %
Cluster 10 (109 cells)	12 cells / 11.01 %	72 cells / 66.06 %	25 cells / 22.94 %	0 cells / 0.00 %	0 cells / 0.00 %
Cluster 11 (51 cells)	47 cells / 92.16 %	4 cells / 7.84 %	0 cells / 0.00 %	0 cells / 0.00 %	0 cells / 0.00 %
Correct identified > 70%	145 / 300 (48.33 %)	82 / 573 (14.31 %)	168 / 362 (46.41 %)	0 / 17 (0.00 %)	0 / 23 (0.00 %)
Correct identified > 80%	145 / 300 (48.33 %)	0 / 573 (0.00 %)	168 / 362 (46.41 %)	0 / 17 (0.00 %)	0 / 23 (0.00 %)
Correct identified > 90%	47 / 300 (15.67 %)	0 / 573 (0.00 %)	0 / 362 (0.00 %)	0 / 17 (0.00 %)	0 / 23 (0.00 %)

Table 10: Computations with a 100-nearest neighbors graph.

Cluster	G1 (300 cells)	early-S (573 cells)	late-S/G2 (362 cells)	post-M (17 cells)	pre-M (23 cells)
Cluster 0 (119 cells)	0 cells / 0.00 %	80 cells / 67.23 %	38 cells / 31.93 %	0 cells / 0.00 %	1 cell / 0.84 %
Cluster 1 (227 cells)	111 cells / 48.90 %	116 cells / 51.10 %	0 cells / 0.00 %	0 cells / 0.00 %	0 cells / 0.00 %
Cluster 2 (210 cells)	0 cells / 0.00 %	146 cells / 69.52 %	61 cells / 29.05 %	0 cells / 0.00 %	3 cells / 1.43 %
Cluster 3 (148 cells)	137 cells / 92.57 %	11 cells / 7.43 %	0 cells / 0.00 %	0 cells / 0.00 %	0 cells / 0.00 %
Cluster 4 (75 cells)	0 cells / 0.00 %	13 cells / 17.33 %	62 cells / 82.67 %	0 cells / 0.00 %	0 cells / 0.00 %
Cluster 5 (100 cells)	0 cells / 0.00 %	27 cells / 27.00 %	73 cells / 73.00 %	0 cells / 0.00 %	0 cells / 0.00 %
Cluster 6 (53 cells)	0 cells / 0.00 %	8 cells / 15.09 %	34 cells / 64.15 %	0 cells / 0.00 %	11 cells / 20.75 %
Cluster 7 (81 cells)	0 cells / 0.00 %	12 cells / 14.81 %	67 cells / 82.72 %	0 cells / 0.00 %	2 cells / 2.47 %
Cluster 8 (48 cells)	0 cells / 0.00 %	23 cells / 47.92 %	21 cells / 43.75 %	0 cells / 0.00 %	4 cells / 8.33 %
Cluster 9 (139 cells)	35 cells / 25.18 %	98 cells / 70.50 %	6 cells / 4.32 %	0 cells / 0.00 %	0 cells / 0.00 %
Cluster 10 (50 cells)	11 cells / 22.00 %	39 cells / 78.00 %	0 cells / 0.00 %	0 cells / 0.00 %	0 cells / 0.00 %
Cluster 11 (25 cells)	6 cells / 24.00 %	0 cells / 0.00 %	0 cells / 0.00 %	17 cells / 68.00 %	2 cells / 8.00 %
Correct identified > 70%	137 / 300 (45.67 %)	137 / 573 (23.91 %)	202 / 362 (55.80 %)	0 / 17 (0.00 %)	0 / 23 (0.00 %)
Correct identified > 80%	137 / 300 (45.67 %)	0 / 573 (0.00 %)	129 / 362 (35.64 %)	0 / 17 (0.00 %)	0 / 23 (0.00 %)
Correct identified > 90%	137 / 300 (45.67 %)	0 / 573 (0.00 %)	0 / 362 (0.00 %)	0 / 17 (0.00 %)	0 / 23 (0.00 %)

Table 11: Computations with a 500-nearest neighbors graph.

4.5 Cluster algorithms

Cluster	G1 (300 cells)	early-S (573 cells)	late-S/G2 (362 cells)	post-M (17 cells)	pre-M (23 cells)
Cluster 0 (126 cells)	1 cell / 0.79 %	112 cells / 88.89 %	13 cells / 10.32 %	0 cells / 0.00 %	0 cells / 0.00 %
Cluster 1 (114 cells)	97 cells / 85.09 %	17 cells / 14.91 %	0 cells / 0.00 %	0 cells / 0.00 %	0 cells / 0.00 %
Cluster 2 (131 cells)	0 cells / 0.00 %	9 cells / 6.87 %	122 cells / 93.13 %	0 cells / 0.00 %	0 cells / 0.00 %
Cluster 3 (104 cells)	0 cells / 0.00 %	70 cells / 67.31 %	34 cells / 32.69 %	0 cells / 0.00 %	0 cells / 0.00 %
Cluster 4 (107 cells)	23 cells / 21.50 %	83 cells / 77.57 %	1 cell / 0.93 %	0 cells / 0.00 %	0 cells / 0.00 %
Cluster 5 (59 cells)	59 cells / 100.00 %	0 cells / 0.00 %	0 cells / 0.00 %	0 cells / 0.00 %	0 cells / 0.00 %
Cluster 6 (51 cells)	13 cells / 25.49 %	0 cells / 0.00 %	0 cells / 0.00 %	17 cells / 33.33 %	21 cells / 41.18 %
Cluster 7 (118 cells)	44 cells / 37.29 %	73 cells / 61.86 %	1 cell / 0.85 %	0 cells / 0.00 %	0 cells / 0.00 %
Cluster 8 (142 cells)	1 cell / 0.70 %	123 cells / 86.62 %	18 cells / 12.68 %	0 cells / 0.00 %	0 cells / 0.00 %
Cluster 9 (101 cells)	0 cells / 0.00 %	16 cells / 15.84 %	83 cells / 82.18 %	0 cells / 0.00 %	2 cells / 1.98 %
Cluster 10 (112 cells)	62 cells / 55.36 %	50 cells / 44.64 %	0 cells / 0.00 %	0 cells / 0.00 %	0 cells / 0.00 %
Cluster 11 (110 cells)	0 cells / 0.00 %	20 cells / 18.18 %	90 cells / 81.82 %	0 cells / 0.00 %	0 cells / 0.00 %
Correct identified > 70%	156 / 300 (52.00 %)	318 / 573 (55.50 %)	295 / 362 (81.49 %)	0 / 17 (0.00 %)	0 / 23 (0.00 %)
Correct identified > 80%	156 / 300 (52.00 %)	235 / 573 (41.01 %)	295 / 362 (81.49 %)	0 / 17 (0.00 %)	0 / 23 (0.00 %)
Correct identified > 90%	59 / 300 (19.67 %)	0 / 573 (0.00 %)	122 / 362 (33.70 %)	0 / 17 (0.00 %)	0 / 23 (0.00 %)

Table 12: k-means

Cluster	G1 (300 cells)	early-S (573 cells)	late-S/G2 (362 cells)	post-M (17 cells)	pre-M (23 cells)
Cluster 0 (113 cells)	110 cells / 97.35 %	3 cells / 2.65 %	0 cells / 0.00 %	0 cells / 0.00 %	0 cells / 0.00 %
Cluster 1 (208 cells)	67 cells / 32.21 %	139 cells / 66.83 %	2 cells / 0.96 %	0 cells / 0.00 %	0 cells / 0.00 %
Cluster 2 (198 cells)	0 cells / 0.00 %	21 cells / 10.61 %	176 cells / 88.89 %	0 cells / 0.00 %	1 cell / 0.51 %
Cluster 3 (136 cells)	1 cell / 0.74 %	93 cells / 68.38 %	42 cells / 30.88 %	0 cells / 0.00 %	0 cells / 0.00 %
Cluster 4 (47 cells)	22 cells / 46.81 %	0 cells / 0.00 %	0 cells / 0.00 %	17 cells / 36.17 %	8 cells / 17.02 %
Cluster 5 (126 cells)	0 cells / 0.00 %	110 cells / 87.30 %	16 cells / 12.70 %	0 cells / 0.00 %	0 cells / 0.00 %
Cluster 6 (118 cells)	0 cells / 0.00 %	17 cells / 14.41 %	101 cells / 85.59 %	0 cells / 0.00 %	0 cells / 0.00 %
Cluster 7 (72 cells)	61 cells / 84.72 %	11 cells / 15.28 %	0 cells / 0.00 %	0 cells / 0.00 %	0 cells / 0.00 %
Cluster 8 (66 cells)	5 cells / 7.58 %	61 cells / 92.42 %	0 cells / 0.00 %	0 cells / 0.00 %	0 cells / 0.00 %
Cluster 9 (71 cells)	28 cells / 39.44 %	43 cells / 60.56 %	0 cells / 0.00 %	0 cells / 0.00 %	0 cells / 0.00 %
Cluster 10 (91 cells)	6 cells / 6.59 %	69 cells / 75.82 %	16 cells / 17.58 %	0 cells / 0.00 %	0 cells / 0.00 %
Cluster 11 (29 cells)	0 cells / 0.00 %	6 cells / 20.69 %	9 cells / 31.03 %	0 cells / 0.00 %	14 cells / 48.28 %
Correct identified > 70%	171 / 300 (57.00 %)	240 / 573 (41.88 %)	277 / 362 (76.52 %)	0 / 17 (0.00 %)	0 / 23 (0.00 %)
Correct identified > 80%	171 / 300 (57.00 %)	171 / 573 (29.84 %)	277 / 362 (76.52 %)	0 / 17 (0.00 %)	0 / 23 (0.00 %)
Correct identified > 90%	110 / 300 (36.67 %)	61 / 573 (10.65 %)	0 / 362 (0.00 %)	0 / 17 (0.00 %)	0 / 23 (0.00 %)

Table 13: agglomerative ward

Cluster	G1 (300 cells)	early-S (573 cells)	late-S/G2 (362 cells)	post-M (17 cells)	pre-M (23 cells)
Cluster 0 (102 cells)	0 cells / 0.00 %	72 cells / 70.59 %	30 cells / 29.41 %	0 cells / 0.00 %	0 cells / 0.00 %
Cluster 1 (90 cells)	0 cells / 0.00 %	12 cells / 13.33 %	78 cells / 86.67 %	0 cells / 0.00 %	0 cells / 0.00 %
Cluster 2 (221 cells)	0 cells / 0.00 %	25 cells / 11.31 %	193 cells / 87.33 %	0 cells / 0.00 %	3 cells / 1.36 %
Cluster 3 (59 cells)	22 cells / 37.29 %	0 cells / 0.00 %	0 cells / 0.00 %	17 cells / 28.81 %	20 cells / 33.90 %
Cluster 4 (160 cells)	0 cells / 0.00 %	137 cells / 85.62 %	23 cells / 14.37 %	0 cells / 0.00 %	0 cells / 0.00 %
Cluster 5 (158 cells)	49 cells / 31.01 %	108 cells / 68.35 %	1 cell / 0.63 %	0 cells / 0.00 %	0 cells / 0.00 %
Cluster 6 (159 cells)	121 cells / 76.10 %	38 cells / 23.90 %	0 cells / 0.00 %	0 cells / 0.00 %	0 cells / 0.00 %
Cluster 7 (120 cells)	69 cells / 57.50 %	51 cells / 42.50 %	0 cells / 0.00 %	0 cells / 0.00 %	0 cells / 0.00 %
Cluster 8 (36 cells)	36 cells / 100.00 %	0 cells / 0.00 %	0 cells / 0.00 %	0 cells / 0.00 %	0 cells / 0.00 %
Cluster 9 (37 cells)	0 cells / 0.00 %	23 cells / 62.16 %	14 cells / 37.84 %	0 cells / 0.00 %	0 cells / 0.00 %
Cluster 10 (85 cells)	1 cell / 1.18 %	61 cells / 71.76 %	23 cells / 27.06 %	0 cells / 0.00 %	0 cells / 0.00 %
Cluster 11 (48 cells)	2 cells / 4.17 %	46 cells / 95.83 %	0 cells / 0.00 %	0 cells / 0.00 %	0 cells / 0.00 %
Correct identified > 70%	157 / 300 (52.33 %)	316 / 573 (55.15 %)	271 / 362 (74.86 %)	0 / 17 (0.00 %)	0 / 23 (0.00 %)
Correct identified > 80%	36 / 300 (12.00 %)	183 / 573 (31.94 %)	271 / 362 (74.86 %)	0 / 17 (0.00 %)	0 / 23 (0.00 %)
Correct identified > 90%	36 / 300 (12.00 %)	46 / 573 (8.03 %)	0 / 362 (0.00 %)	0 / 17 (0.00 %)	0 / 23 (0.00 %)

Table 14: agglomerative complete

Cluster	G1 (300 cells)	early-S (573 cells)	late-S/G2 (362 cells)	post-M (17 cells)	pre-M (23 cells)
Cluster 0 (95 cells)	0 cells / 0.00 %	62 cells / 65.26 %	33 cells / 34.74 %	0 cells / 0.00 %	0 cells / 0.00 %
Cluster 1 (163 cells)	0 cells / 0.00 %	143 cells / 87.73 %	20 cells / 12.27 %	0 cells / 0.00 %	0 cells / 0.00 %
Cluster 2 (98 cells)	3 cells / 3.06 %	36 cells / 36.73 %	58 cells / 59.18 %	0 cells / 0.00 %	1 cell / 1.02 %
Cluster 3 (63 cells)	63 cells / 100.00 %	0 cells / 0.00 %	0 cells / 0.00 %	0 cells / 0.00 %	0 cells / 0.00 %
Cluster 4 (163 cells)	51 cells / 31.29 %	111 cells / 68.10 %	1 cell / 0.61 %	0 cells / 0.00 %	0 cells / 0.00 %
Cluster 5 (52 cells)	15 cells / 28.85 %	37 cells / 71.15 %	0 cells / 0.00 %	0 cells / 0.00 %	0 cells / 0.00 %
Cluster 6 (120 cells)	101 cells / 84.17 %	19 cells / 15.83 %	0 cells / 0.00 %	0 cells / 0.00 %	0 cells / 0.00 %
Cluster 7 (87 cells)	0 cells / 0.00 %	9 cells / 10.34 %	78 cells / 89.66 %	0 cells / 0.00 %	0 cells / 0.00 %
Cluster 8 (114 cells)	63 cells / 55.26 %	51 cells / 44.74 %	0 cells / 0.00 %	0 cells / 0.00 %	0 cells / 0.00 %
Cluster 9 (98 cells)	1 cell / 1.02 %	91 cells / 92.86 %	6 cells / 6.12 %	0 cells / 0.00 %	0 cells / 0.00 %
Cluster 10 (180 cells)	0 cells / 0.00 %	14 cells / 7.78 %	166 cells / 92.22 %	0 cells / 0.00 %	0 cells / 0.00 %
Cluster 11 (42 cells)	3 cells / 7.14 %	0 cells / 0.00 %	0 cells / 0.00 %	17 cells / 40.48 %	22 cells / 52.38 %
Correct identified > 70%	164 / 300 (54.67 %)	271 / 573 (47.29 %)	244 / 362 (67.40 %)	0 / 17 (0.00 %)	0 / 23 (0.00 %)
Correct identified > 80%	164 / 300 (54.67 %)	234 / 573 (40.84 %)	244 / 362 (67.40 %)	0 / 17 (0.00 %)	0 / 23 (0.00 %)
Correct identified > 90%	63 / 300 (21.00 %)	91 / 573 (15.88 %)	166 / 362 (45.86 %)	0 / 17 (0.00 %)	0 / 23 (0.00 %)

Table 15: agglomerative average

Cluster	G1 (300 cells)	early-S (573 cells)	late-S/G2 (362 cells)	post-M (17 cells)	pre-M (23 cells)
Cluster 0 (1263 cells)	297 cells / 23.52 %	567 cells / 44.89 %	359 cells / 28.42 %	17 cells / 1.35 %	23 cells / 1.82 %
Cluster 1 (2 cells)	0 cells / 0.00 %	1 cell / 50.00 %	1 cell / 50.00 %	0 cells / 0.00 %	0 cells / 0.00 %
Cluster 2 (1 cells)	0 cells / 0.00 %	0 cells / 0.00 %	1 cell / 100.00 %	0 cells / 0.00 %	0 cells / 0.00 %
Cluster 3 (1 cells)	0 cells / 0.00 %	1 cell / 100.00 %	0 cells / 0.00 %	0 cells / 0.00 %	0 cells / 0.00 %
Cluster 4 (1 cells)	1 cell / 100.00 %	0 cells / 0.00 %	0 cells / 0.00 %	0 cells / 0.00 %	0 cells / 0.00 %
Cluster 5 (1 cells)	1 cell / 100.00 %	0 cells / 0.00 %	0 cells / 0.00 %	0 cells / 0.00 %	0 cells / 0.00 %
Cluster 6 (1 cells)	0 cells / 0.00 %	0 cells / 0.00 %	1 cell / 100.00 %	0 cells / 0.00 %	0 cells / 0.00 %
Cluster 7 (1 cells)	0 cells / 0.00 %	1 cell / 100.00 %	0 cells / 0.00 %	0 cells / 0.00 %	0 cells / 0.00 %
Cluster 8 (1 cells)	0 cells / 0.00 %	1 cell / 100.00 %	0 cells / 0.00 %	0 cells / 0.00 %	0 cells / 0.00 %
Cluster 9 (1 cells)	1 cell / 100.00 %	0 cells / 0.00 %	0 cells / 0.00 %	0 cells / 0.00 %	0 cells / 0.00 %
Cluster 10 (1 cells)	0 cells / 0.00 %	1 cell / 100.00 %	0 cells / 0.00 %	0 cells / 0.00 %	0 cells / 0.00 %
Cluster 11 (1 cells)	0 cells / 0.00 %	1 cell / 100.00 %	0 cells / 0.00 %	0 cells / 0.00 %	0 cells / 0.00 %
Correct identified > 70%	3 / 300 (1.00 %)	5 / 573 (0.87 %)	2 / 362 (0.55 %)	0 / 17 (0.00 %)	0 / 23 (0.00 %)
Correct identified > 80%	3 / 300 (1.00 %)	5 / 573 (0.87 %)	2 / 362 (0.55 %)	0 / 17 (0.00 %)	0 / 23 (0.00 %)
Correct identified > 90%	3 / 300 (1.00 %)	5 / 573 (0.87 %)	2 / 362 (0.55 %)	0 / 17 (0.00 %)	0 / 23 (0.00 %)

Table 16: agglomerative single

Cluster	G1 (300 cells)	early-S (573 cells)	late-S/G2 (362 cells)	post-M (17 cells)	pre-M (23 cells)
Cluster 0 (195 cells)	3 cells / 1.54 %	35 cells / 17.95 %	119 cells / 61.03 %	17 cells / 8.72 %	21 cells / 10.77 %
Cluster 1 (93 cells)	0 cells / 0.00 %	70 cells / 75.27 %	23 cells / 24.73 %	0 cells / 0.00 %	0 cells / 0.00 %
Cluster 2 (206 cells)	0 cells / 0.00 %	19 cells / 9.22 %	185 cells / 89.81 %	0 cells / 0.00 %	2 cells / 0.97 %
Cluster 3 (76 cells)	5 cells / 6.58 %	66 cells / 86.84 %	5 cells / 6.58 %	0 cells / 0.00 %	0 cells / 0.00 %
Cluster 4 (87 cells)	18 cells / 20.69 %	68 cells / 78.16 %	1 cell / 1.15 %	0 cells / 0.00 %	0 cells / 0.00 %
Cluster 5 (52 cells)	15 cells / 28.85 %	37 cells / 71.15 %	0 cells / 0.00 %	0 cells / 0.00 %	0 cells / 0.00 %
Cluster 6 (114 cells)	1 cell / 0.88 %	100 cells / 87.72 %	13 cells / 11.40 %	0 cells / 0.00 %	0 cells / 0.00 %
Cluster 7 (100 cells)	74 cells / 74.00 %	26 cells / 26.00 %	0 cells / 0.00 %	0 cells / 0.00 %	0 cells / 0.00 %
Cluster 8 (121 cells)	2 cells / 1.65 %	103 cells / 85.12 %	16 cells / 13.22 %	0 cells / 0.00 %	0 cells / 0.00 %
Cluster 9 (77 cells)	74 cells / 96.10 %	3 cells / 3.90 %	0 cells / 0.00 %	0 cells / 0.00 %	0 cells / 0.00 %
Cluster 10 (99 cells)	53 cells / 53.54 %	46 cells / 46.46 %	0 cells / 0.00 %	0 cells / 0.00 %	0 cells / 0.00 %
Cluster 11 (55 cells)	55 cells / 100.00 %	0 cells / 0.00 %	0 cells / 0.00 %	0 cells / 0.00 %	0 cells / 0.00 %
Correct identified > 70%	203 / 300 (67.67 %)	444 / 573 (77.49 %)	185 / 362 (51.10 %)	0 / 17 (0.00 %)	0 / 23 (0.00 %)
Correct identified > 80%	129 / 300 (43.00 %)	269 / 573 (46.95 %)	185 / 362 (51.10 %)	0 / 17 (0.00 %)	0 / 23 (0.00 %)
Correct identified > 90%	129 / 300 (43.00 %)	0 / 573 (0.00 %)	0 / 362 (0.00 %)	0 / 17 (0.00 %)	0 / 23 (0.00 %)

Table 17: birch

4.6 Competing approaches

Cluster	G1 (300 cells)	early-S (573 cells)	late-S/G2 (362 cells)	post-M (17 cells)	pre-M (23 cells)
Cluster 0 (28 cells)	1 cell / 3.57 %	0 cells / 0.00 %	0 cells / 0.00 %	17 cells / 60.71 %	10 cells / 35.71 %
Cluster 1 (123 cells)	85 cells / 69.11 %	38 cells / 30.89 %	0 cells / 0.00 %	0 cells / 0.00 %	0 cells / 0.00 %
Cluster 2 (119 cells)	30 cells / 25.21 %	86 cells / 72.27 %	3 cells / 2.52 %	0 cells / 0.00 %	0 cells / 0.00 %
Cluster 3 (66 cells)	0 cells / 0.00 %	32 cells / 48.48 %	34 cells / 51.52 %	0 cells / 0.00 %	0 cells / 0.00 %
Cluster 4 (100 cells)	0 cells / 0.00 %	4 cells / 4.00 %	83 cells / 83.00 %	0 cells / 0.00 %	13 cells / 13.00 %
Cluster 5 (121 cells)	0 cells / 0.00 %	6 cells / 4.96 %	115 cells / 95.04 %	0 cells / 0.00 %	0 cells / 0.00 %
Cluster 6 (58 cells)	58 cells / 100.00 %	0 cells / 0.00 %	0 cells / 0.00 %	0 cells / 0.00 %	0 cells / 0.00 %
Cluster 7 (172 cells)	16 cells / 9.30 %	151 cells / 87.79 %	5 cells / 2.91 %	0 cells / 0.00 %	0 cells / 0.00 %
Cluster 8 (105 cells)	101 cells / 96.19 %	4 cells / 3.81 %	0 cells / 0.00 %	0 cells / 0.00 %	0 cells / 0.00 %
Cluster 9 (165 cells)	9 cells / 5.45 %	137 cells / 83.03 %	19 cells / 11.52 %	0 cells / 0.00 %	0 cells / 0.00 %
Cluster 10 (167 cells)	0 cells / 0.00 %	80 cells / 47.90 %	87 cells / 52.10 %	0 cells / 0.00 %	0 cells / 0.00 %
Cluster 11 (51 cells)	0 cells / 0.00 %	35 cells / 68.63 %	16 cells / 31.37 %	0 cells / 0.00 %	0 cells / 0.00 %
Correct identified > 70%	159 / 300 (53.00 %)	374 / 573 (65.27 %)	198 / 362 (54.70 %)	0 / 17 (0.00 %)	0 / 23 (0.00 %)
Correct identified > 80%	159 / 300 (53.00 %)	288 / 573 (50.26 %)	198 / 362 (54.70 %)	0 / 17 (0.00 %)	0 / 23 (0.00 %)
Correct identified > 90%	159 / 300 (53.00 %)	0 / 573 (0.00 %)	115 / 362 (31.77 %)	0 / 17 (0.00 %)	0 / 23 (0.00 %)

Table 18: Overlaps of detect clusters with known cell cycle stages from Nagano *et al.* (2017). Clustering with Zhou's scHiCluster.

Cluster	G1 (300 cells)	early-S (573 cells)	late-S/G2 (362 cells)	post-M (17 cells)	pre-M (23 cells)
Cluster 0 (769 cells)	177 cells / 23.02 %	352 cells / 45.77 %	216 cells / 28.09 %	10 cells / 1.30 %	14 cells / 1.82 %
Cluster 1 (1 cells)	0 cells / 0.00 %	1 cell / 100.00 %	0 cells / 0.00 %	0 cells / 0.00 %	0 cells / 0.00 %
Cluster 2 (1 cells)	0 cells / 0.00 %	0 cells / 0.00 %	1 cell / 100.00 %	0 cells / 0.00 %	0 cells / 0.00 %
Cluster 3 (1 cells)	0 cells / 0.00 %	0 cells / 0.00 %	1 cell / 100.00 %	0 cells / 0.00 %	0 cells / 0.00 %
Cluster 4 (1 cells)	0 cells / 0.00 %	0 cells / 0.00 %	1 cell / 100.00 %	0 cells / 0.00 %	0 cells / 0.00 %
Cluster 5 (1 cells)	0 cells / 0.00 %	0 cells / 0.00 %	1 cell / 100.00 %	0 cells / 0.00 %	0 cells / 0.00 %
Cluster 6 (477 cells)	112 cells / 23.48 %	210 cells / 44.03 %	139 cells / 29.14 %	7 cells / 1.47 %	9 cells / 1.89 %
Cluster 7 (13 cells)	4 cells / 30.77 %	7 cells / 53.85 %	2 cells / 15.38 %	0 cells / 0.00 %	0 cells / 0.00 %
Cluster 8 (1 cells)	1 cell / 100.00 %	0 cells / 0.00 %	0 cells / 0.00 %	0 cells / 0.00 %	0 cells / 0.00 %
Cluster 9 (4 cells)	2 cells / 50.00 %	1 cell / 25.00 %	1 cell / 25.00 %	0 cells / 0.00 %	0 cells / 0.00 %
Cluster 10 (4 cells)	4 cells / 100.00 %	0 cells / 0.00 %	0 cells / 0.00 %	0 cells / 0.00 %	0 cells / 0.00 %
Cluster 11 (2 cells)	0 cells / 0.00 %	2 cells / 100.00 %	0 cells / 0.00 %	0 cells / 0.00 %	0 cells / 0.00 %
Correct identified > 70%	5 / 300 (1.67 %)	3 / 573 (0.52 %)	4 / 362 (1.10 %)	0 / 17 (0.00 %)	0 / 23 (0.00 %)
Correct identified > 80%	5 / 300 (1.67 %)	3 / 573 (0.52 %)	4 / 362 (1.10 %)	0 / 17 (0.00 %)	0 / 23 (0.00 %)
Correct identified > 90%	5 / 300 (1.67 %)	3 / 573 (0.52 %)	4 / 362 (1.10 %)	0 / 17 (0.00 %)	0 / 23 (0.00 %)

Table 19: Scikit-learn k-nearest neighbor with k=1275, with spectral clustering.

Cluster	G1 (300 cells)	early-S (573 cells)	late-S/G2 (362 cells)	post-M (17 cells)	pre-M (23 cells)
Cluster 0 (25 cells)	5 cells / 20.00 %	20 cells / 80.00 %	0 cells / 0.00 %	0 cells / 0.00 %	0 cells / 0.00 %
Cluster 1 (21 cells)	1 cell / 4.76 %	18 cells / 85.71 %	2 cells / 9.52 %	0 cells / 0.00 %	0 cells / 0.00 %
Cluster 2 (1 cells)	0 cells / 0.00 %	0 cells / 0.00 %	1 cell / 100.00 %	0 cells / 0.00 %	0 cells / 0.00 %
Cluster 3 (1 cells)	0 cells / 0.00 %	1 cell / 100.00 %	0 cells / 0.00 %	0 cells / 0.00 %	0 cells / 0.00 %
Cluster 4 (9 cells)	3 cells / 33.33 %	6 cells / 66.67 %	0 cells / 0.00 %	0 cells / 0.00 %	0 cells / 0.00 %
Cluster 5 (1 cells)	0 cells / 0.00 %	1 cell / 100.00 %	0 cells / 0.00 %	0 cells / 0.00 %	0 cells / 0.00 %
Cluster 6 (1 cells)	0 cells / 0.00 %	1 cell / 100.00 %	0 cells / 0.00 %	0 cells / 0.00 %	0 cells / 0.00 %
Cluster 7 (1202 cells)	287 cells / 23.88 %	518 cells / 43.09 %	357 cells / 29.70 %	17 cells / 1.41 %	23 cells / 1.91 %
Cluster 8 (1 cells)	0 cells / 0.00 %	1 cell / 100.00 %	0 cells / 0.00 %	0 cells / 0.00 %	0 cells / 0.00 %
Cluster 9 (8 cells)	3 cells / 37.50 %	4 cells / 50.00 %	1 cell / 12.50 %	0 cells / 0.00 %	0 cells / 0.00 %
Cluster 10 (4 cells)	1 cell / 25.00 %	3 cells / 75.00 %	0 cells / 0.00 %	0 cells / 0.00 %	0 cells / 0.00 %
Cluster 11 (1 cells)	0 cells / 0.00 %	0 cells / 0.00 %	1 cell / 100.00 %	0 cells / 0.00 %	0 cells / 0.00 %
Correct identified > 70%	0 / 300 (0.00 %)	45 / 573 (7.85 %)	2 / 362 (0.55 %)	0 / 17 (0.00 %)	0 / 23 (0.00 %)
Correct identified > 80%	0 / 300 (0.00 %)	42 / 573 (7.33 %)	2 / 362 (0.55 %)	0 / 17 (0.00 %)	0 / 23 (0.00 %)
Correct identified > 90%	0 / 300 (0.00 %)	4 / 573 (0.70 %)	2 / 362 (0.55 %)	0 / 17 (0.00 %)	0 / 23 (0.00 %)

Table 20: PCA on raw data with spectral clustering.

Cluster	G1 (300 cells)	early-S (573 cells)	late-S/G2 (362 cells)	post-M (17 cells)	pre-M (23 cells)
Cluster 0 (216 cells)	79 cells / 36.57 %	101 cells / 46.76 %	31 cells / 14.35 %	1 cell / 0.46 %	4 cells / 1.85 %
Cluster 1 (58 cells)	0 cells / 0.00 %	33 cells / 56.90 %	25 cells / 43.10 %	0 cells / 0.00 %	0 cells / 0.00 %
Cluster 2 (108 cells)	24 cells / 22.22 %	58 cells / 53.70 %	26 cells / 24.07 %	0 cells / 0.00 %	0 cells / 0.00 %
Cluster 3 (106 cells)	0 cells / 0.00 %	82 cells / 77.36 %	24 cells / 22.64 %	0 cells / 0.00 %	0 cells / 0.00 %
Cluster 4 (226 cells)	82 cells / 36.28 %	110 cells / 48.67 %	30 cells / 13.27 %	2 cells / 0.88 %	2 cells / 0.88 %
Cluster 5 (99 cells)	2 cells / 2.02 %	13 cells / 13.13 %	77 cells / 77.78 %	0 cells / 0.00 %	7 cells / 7.07 %
Cluster 6 (197 cells)	112 cells / 56.85 %	64 cells / 32.49 %	16 cells / 8.12 %	0 cells / 0.00 %	5 cells / 2.54 %
Cluster 7 (80 cells)	0 cells / 0.00 %	6 cells / 7.50 %	70 cells / 87.50 %	0 cells / 0.00 %	4 cells / 5.00 %
Cluster 8 (92 cells)	1 cell / 1.09 %	84 cells / 91.30 %	7 cells / 7.61 %	0 cells / 0.00 %	0 cells / 0.00 %
Cluster 9 (15 cells)	0 cells / 0.00 %	0 cells / 0.00 %	0 cells / 0.00 %	14 cells / 93.33 %	1 cell / 6.67 %
Cluster 10 (3 cells)	0 cells / 0.00 %	0 cells / 0.00 %	3 cells / 100.00 %	0 cells / 0.00 %	0 cells / 0.00 %
Cluster 11 (75 cells)	0 cells / 0.00 %	22 cells / 29.33 %	53 cells / 70.67 %	0 cells / 0.00 %	0 cells / 0.00 %
Correct identified > 70%	0 / 300 (0.00 %)	166 / 573 (28.97 %)	203 / 362 (56.08 %)	14 / 17 (82.35 %)	0 / 23 (0.00 %)
Correct identified > 80%	0 / 300 (0.00 %)	84 / 573 (14.66 %)	73 / 362 (20.17 %)	14 / 17 (82.35 %)	0 / 23 (0.00 %)
Correct identified > 90%	0 / 300 (0.00 %)	84 / 573 (14.66 %)	3 / 362 (0.83 %)	14 / 17 (82.35 %)	0 / 23 (0.00 %)

Table 21: Clustering on raw interaction matrices with k-means clustering.

5 Runtimes on 10 kb resolution

Method	Runtime	Memory
Raw and K-Means	-	> 1 TB
Raw and Spectral	-	> 1 TB
PCA and K-Means	-	> 1 TB
PCA and Spectral	-	> 1 TB
scikit-learn k-nn k = 2633 and k-means	-	> 1 TB
scikit-learn k-nn k = 2633 and Spectral	-	> 1 TB
scHicClusterMinHash k = 2633 and k-means	06:17 min	40.1 GB
scHicClusterMinHash k = 2633 and Spectral	06:26 min	40.1 GB
scHicClusterMinHash eucl. k = 2633 and k-means	08:10 min	40.1 GB
scHicClusterMinHash eucl. k = 2633 and Spectral	08:08 min	40.1 GB
Zhou's scHiCluster CPU	- (*)	> 970 GB

Table 22: Runtimes and memory usage on 10 kb resolution, 2633 cells on a single-cell Hi-C matrix. Data from Nagano *et al.* (2017) Diploid cells, with 12 clusters. For clustering k-means and spectral clustering are used, scHicClusterMinHash with 800 hash functions, k=2472, applied PCA and 100 principal components for clustering. (*) Zhou's scHiCluster computed 97 hours the data for chromosome 10 and requested 970 GB of memory, the computation was canceled after this time. All results computed on 2x Intel XEON E5-2630 v4 @ 2.20GHz 2x 10 cores / 2x 20 threads, 1 TB memory.

Method	Runtime	Memory
Raw and K-Means	-	> 128 GB
Raw and Spectral	-	> 128 GB
PCA and K-Means	-	> 128 GB
PCA and Spectral	-	> 128 GB
scikit-learn k-nn k = 2632 and K-means	-	> 128 GB
scikit-learn k-nn k = 2632 and Spectral	-	> 128 GB
MinHash k = 2633 and K-means	03:39 min	40.1 GB
MinHash k = 2633 and Spectral	03:41 min	40.1 GB
MinHash k = 2633 and K-means (–saveMemory 1%)	12:53 min	12.5 GB
MinHash k = 2633 and K-means intra-chromosomal	08:26 min	35.8 GB
MinHash k = 2633 and Spectral intra-chromosomal	08:55 min	35.8 GB
MinHash euclidean k = 2633 and K-means	06:39 min	40.1 GB
MinHash euclidean k = 2633 and Spectral	06:39 min	40.1 GB
MinHash euclidean k = 2633 and K-means intra-chromosomal	11:47 min	35.8 GB
MinHash euclidean k = 2633 and Spectral intra-chromosomal	11:49 min	35.8 GB
Zhou's scHiCluster CPU	-	> 128 GB
Zhou's scHiCluster GPU	-	> 128 GB

Table 23: Runtimes and memory usage with 10 kb resolution on a single-cell Hi-C matrix with 2633 cells. Normalized to a read coverage of 100,000 reads and interaction values smaller 1 are *kept*. Data from Nagano *et al.* (2017) Diploid cells, with 12 clusters. For clustering K-means and spectral clustering are used, MinHash with 800 hash functions. All results computed on AMD Ryzen 3700X 8 cores / 16 threads, 128 GB memory; Nvidia GTX 1070 8 GB memory.

6 Nagano 2017 10 kb data

MinHash on 10 kb data from Nagano with 1088 cells. Parameters: differing number of hash functions, 44 principal components, spectral clustering. UMAP parameters: k-neighbors 36, components 9, min distance 0.05.

Cluster	G1 (249 cells)	early-S (448 cells)	late-S/G2 (341 cells)	post-M (17 cells)	pre-M (23 cells)
Cluster 0 (71 cells)	17 cells / 23.94 %	12 cells / 16.90 %	39 cells / 54.93 %	0 cells / 0.00 %	3 cells / 4.23 %
Cluster 1 (67 cells)	2 cells / 2.99 %	60 cells / 89.55 %	5 cells / 7.46 %	0 cells / 0.00 %	0 cells / 0.00 %
Cluster 2 (75 cells)	1 cell / 1.33 %	44 cells / 58.67 %	28 cells / 37.33 %	1 cell / 1.33 %	1 cell / 1.33 %
Cluster 3 (127 cells)	8 cells / 6.30 %	88 cells / 69.29 %	24 cells / 18.90 %	5 cells / 3.94 %	2 cells / 1.57 %
Cluster 4 (55 cells)	31 cells / 56.36 %	16 cells / 29.09 %	7 cells / 12.73 %	0 cells / 0.00 %	1 cell / 1.82 %
Cluster 5 (64 cells)	1 cell / 1.56 %	19 cells / 29.69 %	44 cells / 68.75 %	0 cells / 0.00 %	0 cells / 0.00 %
Cluster 6 (111 cells)	22 cells / 19.82 %	53 cells / 47.75 %	27 cells / 24.32 %	6 cells / 5.41 %	3 cells / 2.70 %
Cluster 7 (117 cells)	68 cells / 58.12 %	24 cells / 20.51 %	19 cells / 16.24 %	4 cells / 3.42 %	2 cells / 1.71 %
Cluster 8 (54 cells)	17 cells / 31.48 %	21 cells / 38.89 %	13 cells / 24.07 %	0 cells / 0.00 %	3 cells / 5.56 %
Cluster 9 (50 cells)	29 cells / 58.00 %	17 cells / 34.00 %	2 cells / 4.00 %	0 cells / 0.00 %	2 cells / 4.00 %
Cluster 10 (74 cells)	11 cells / 14.86 %	51 cells / 68.92 %	12 cells / 16.22 %	0 cells / 0.00 %	0 cells / 0.00 %
Cluster 11 (53 cells)	29 cells / 54.72 %	15 cells / 28.30 %	9 cells / 16.98 %	0 cells / 0.00 %	0 cells / 0.00 %
Cluster 12 (65 cells)	5 cells / 7.69 %	8 cells / 12.31 %	49 cells / 75.38 %	0 cells / 0.00 %	3 cells / 4.62 %
Cluster 13 (95 cells)	8 cells / 8.42 %	20 cells / 21.05 %	63 cells / 66.32 %	1 cell / 1.05 %	3 cells / 3.16 %
Correct identified > 70%	0 / 249 (0.00 %)	60 / 448 (13.39 %)	49 / 341 (14.37 %)	0 / 17 (0.00 %)	0 / 23 (0.00 %)
Correct identified > 80%	0 / 249 (0.00 %)	60 / 448 (13.39 %)	0 / 341 (0.00 %)	0 / 17 (0.00 %)	0 / 23 (0.00 %)
Correct identified > 90%	0 / 249 (0.00 %)	0 / 448 (0.00 %)	0 / 341 (0.00 %)	0 / 17 (0.00 %)	0 / 23 (0.00 %)

Table 24: 20000 hash functions.

Cluster	G1 (249 cells)	early-S (448 cells)	late-S/G2 (341 cells)	post-M (17 cells)	pre-M (23 cells)
Cluster 0 (71 cells)	0 cells / 0.00 %	24 cells / 33.80 %	47 cells / 66.20 %	0 cells / 0.00 %	0 cells / 0.00 %
Cluster 1 (80 cells)	6 cells / 7.50 %	57 cells / 71.25 %	15 cells / 18.75 %	1 cell / 1.25 %	1 cell / 1.25 %
Cluster 2 (114 cells)	45 cells / 39.47 %	50 cells / 43.86 %	14 cells / 12.28 %	0 cells / 0.00 %	5 cells / 4.39 %
Cluster 3 (93 cells)	11 cells / 11.83 %	20 cells / 21.51 %	56 cells / 60.22 %	0 cells / 0.00 %	6 cells / 6.45 %
Cluster 4 (104 cells)	77 cells / 74.04 %	16 cells / 15.38 %	9 cells / 8.65 %	0 cells / 0.00 %	2 cells / 1.92 %
Cluster 5 (32 cells)	8 cells / 25.00 %	4 cells / 12.50 %	4 cells / 12.50 %	16 cells / 50.00 %	0 cells / 0.00 %
Cluster 6 (82 cells)	0 cells / 0.00 %	34 cells / 41.46 %	48 cells / 58.54 %	0 cells / 0.00 %	0 cells / 0.00 %
Cluster 7 (66 cells)	31 cells / 46.97 %	23 cells / 34.85 %	12 cells / 18.18 %	0 cells / 0.00 %	0 cells / 0.00 %
Cluster 8 (63 cells)	8 cells / 12.70 %	37 cells / 58.73 %	17 cells / 26.98 %	0 cells / 0.00 %	1 cell / 1.59 %
Cluster 9 (58 cells)	12 cells / 20.69 %	26 cells / 44.83 %	16 cells / 27.59 %	0 cells / 0.00 %	4 cells / 6.90 %
Cluster 10 (69 cells)	9 cells / 13.04 %	5 cells / 7.25 %	52 cells / 75.36 %	0 cells / 0.00 %	3 cells / 4.35 %
Cluster 11 (100 cells)	4 cells / 4.00 %	80 cells / 80.00 %	16 cells / 16.00 %	0 cells / 0.00 %	0 cells / 0.00 %
Cluster 12 (75 cells)	1 cell / 1.33 %	52 cells / 69.33 %	22 cells / 29.33 %	0 cells / 0.00 %	0 cells / 0.00 %
Cluster 13 (71 cells)	37 cells / 52.11 %	20 cells / 28.17 %	13 cells / 18.31 %	0 cells / 0.00 %	1 cell / 1.41 %
Correct identified > 70%	77 / 249 (30.92 %)	137 / 448 (30.58 %)	52 / 341 (15.25 %)	0 / 17 (0.00 %)	0 / 23 (0.00 %)
Correct identified > 80%	0 / 249 (0.00 %)	80 / 448 (17.86 %)	0 / 341 (0.00 %)	0 / 17 (0.00 %)	0 / 23 (0.00 %)
Correct identified > 90%	0 / 249 (0.00 %)	0 / 448 (0.00 %)	0 / 341 (0.00 %)	0 / 17 (0.00 %)	0 / 23 (0.00 %)

Table 25: 40000 hash functions.

Cluster	G1 (249 cells)	early-S (448 cells)	late-S/G2 (341 cells)	post-M (17 cells)	pre-M (23 cells)
Cluster 0 (102 cells)	55 cells / 53.92 %	25 cells / 24.51 %	12 cells / 11.76 %	6 cells / 5.88 %	4 cells / 3.92 %
Cluster 1 (151 cells)	84 cells / 55.63 %	42 cells / 27.81 %	20 cells / 13.25 %	0 cells / 0.00 %	5 cells / 3.31 %
Cluster 2 (143 cells)	17 cells / 11.89 %	27 cells / 18.88 %	93 cells / 65.03 %	0 cells / 0.00 %	6 cells / 4.20 %
Cluster 3 (95 cells)	4 cells / 4.21 %	87 cells / 91.58 %	4 cells / 4.21 %	0 cells / 0.00 %	0 cells / 0.00 %
Cluster 4 (79 cells)	21 cells / 26.58 %	37 cells / 46.84 %	21 cells / 26.58 %	0 cells / 0.00 %	0 cells / 0.00 %
Cluster 5 (33 cells)	6 cells / 18.18 %	8 cells / 24.24 %	6 cells / 18.18 %	11 cells / 33.33 %	2 cells / 6.06 %
Cluster 6 (111 cells)	2 cells / 1.80 %	50 cells / 45.05 %	59 cells / 53.15 %	0 cells / 0.00 %	0 cells / 0.00 %
Cluster 7 (32 cells)	14 cells / 43.75 %	11 cells / 34.38 %	7 cells / 21.88 %	0 cells / 0.00 %	0 cells / 0.00 %
Cluster 8 (77 cells)	9 cells / 11.69 %	49 cells / 63.64 %	17 cells / 22.08 %	0 cells / 0.00 %	2 cells / 2.60 %
Cluster 9 (45 cells)	25 cells / 55.56 %	14 cells / 31.11 %	6 cells / 13.33 %	0 cells / 0.00 %	0 cells / 0.00 %
Cluster 10 (23 cells)	3 cells / 13.04 %	14 cells / 60.87 %	6 cells / 26.09 %	0 cells / 0.00 %	0 cells / 0.00 %
Cluster 11 (34 cells)	2 cells / 5.88 %	11 cells / 32.35 %	20 cells / 58.82 %	0 cells / 0.00 %	1 cell / 2.94 %
Cluster 12 (76 cells)	3 cells / 3.95 %	52 cells / 68.42 %	18 cells / 23.68 %	0 cells / 0.00 %	3 cells / 3.95 %
Cluster 13 (77 cells)	4 cells / 5.19 %	21 cells / 27.27 %	52 cells / 67.53 %	0 cells / 0.00 %	0 cells / 0.00 %
Correct identified > 70%	0 / 249 (0.00 %)	87 / 448 (19.42 %)	0 / 341 (0.00 %)	0 / 17 (0.00 %)	0 / 23 (0.00 %)
Correct identified > 80%	0 / 249 (0.00 %)	87 / 448 (19.42 %)	0 / 341 (0.00 %)	0 / 17 (0.00 %)	0 / 23 (0.00 %)
Correct identified > 90%	0 / 249 (0.00 %)	87 / 448 (19.42 %)	0 / 341 (0.00 %)	0 / 17 (0.00 %)	0 / 23 (0.00 %)

Table 26: 50000 hash functions.

7 Cluster results on Ramani 1MB

Cluster	HeLa (269 cells)	HAP1 (254 cells)
Cluster 0 (264 cells)	24 cells / 9.09 %	240 cells / 90.91 %
Cluster 1 (259 cells)	245 cells / 94.59 %	14 cells / 5.41 %
Correct identified > 70%	245 / 269 (91.08 %)	240 / 254 (94.49 %)
Correct identified > 80%	245 / 269 (91.08 %)	240 / 254 (94.49 %)
Correct identified > 90%	245 / 269 (91.08 %)	240 / 254 (94.49 %)

(a) ML1 with two clusters

Cluster	HeLa (269 cells)	HAP1 (254 cells)
Cluster 0 (244 cells)	8 cells / 3.28 %	236 cells / 96.72 %
Cluster 1 (265 cells)	258 cells / 97.36 %	7 cells / 2.64 %
Cluster 2 (14 cells)	3 cells / 21.43 %	11 cells / 78.57 %
Correct identified > 70%	258 / 269 (95.91 %)	247 / 254 (97.24 %)
Correct identified > 80%	258 / 269 (95.91 %)	236 / 254 (92.91 %)
Correct identified > 90%	258 / 269 (95.91 %)	236 / 254 (92.91 %)

(b) ML1 with three clusters

Table 27: Overlaps of detected clusters with known cell types from Ramani *et al.* (2017), ML1 batch. Approximate k-nn with MinHash, spectral clustering, 2000 hash functions, full-nearest neighbors graph, 7 principal components, intra-chromosomal contacts only, umap: n_neighbors 40, min_dist 0.25, n_components 2 for two clusters, n_components 6 for three clusters.

Cluster	HeLa (267 cells)	HAP1 (251 cells)
Cluster 0 (256 cells)	71 cells / 27.7%	185 cells / 72.3%
Cluster 1 (262 cells)	196 cells / 74.8%	66 cells / 25.2%
Correct identified > 70%	196 / 269 (72.86 %)	185 / 254 (72.83 %)
Correct identified > 80%	0 / 269 (0 %)	0 / 254 (0 %)
Correct identified > 90%	0 / 269 (0 %)	0 / 254 (0 %)

(a) ML1 with two clusters

Cluster	HeLa (267 cells)	HAP1 (251 cells)
Cluster 0 (231 cells)	8 cells / 3.4%	223 cells / 96.6%
Cluster 1 (258 cells)	258 cells / 100%	0 cells / 0%
Cluster 2 (29 cells)	1 cell / 3.4%	28 cells / 96.6%
Correct identified > 70%	258 / 267 (96.62 %)	251 / 251 (100 %)
Correct identified > 80%	258 / 267 (96.62 %	251 / 251 (100 %)
Correct identified > 90%	258 / 267 (96.62 %	251 / 251 (100 %)

(b) ML1 with three clusters

Table 28: Overlaps of detected clusters with known cell types from Ramani *et al.* (2017), ML1 batch. Results computed with Zhou's scHiCluster. Five cells had to be removed because they contained chromosomes with no interactions. Zhou's scHiCluster cannot handle this and crashes.

Cluster	K562 (304 cells)	GM12878 (502 cells)
Cluster 0 (372 cells)	241 cells / 64.78 %	131 cells / 35.22 %
Cluster 1 (434 cells)	63 cells / 14.52 %	371 cells / 85.48 %
Correct identified > 70%	0 / 304 (0.00 %)	371 / 502 (73.90 %)
Correct identified > 80%	0 / 304 (0.00 %)	371 / 502 (73.90 %)
Correct identified > 90%	0 / 304 (0.00 %)	0 / 502 (0.00 %)

(a) ML3 with two clusters

Cluster	K562 (304 cells)	GM12878 (502 cells)
Cluster 0 (177 cells)	23 cells / 12.99 %	154 cells / 87.01 %
Cluster 1 (223 cells)	23 cells / 10.31 %	200 cells / 89.69 %
Cluster 2 (208 cells)	184 cells / 88.46 %	24 cells / 11.54 %
Cluster 3 (58 cells)	54 cells / 93.10 %	4 cells / 6.90 %
Cluster 4 (140 cells)	20 cells / 14.29 %	120 cells / 85.71 %
Correct identified > 70%	238 / 304 (78.29 %)	474 / 502 (94.42 %)
Correct identified > 80%	238 / 304 (78.29 %)	474 / 502 (94.42 %)
Correct identified > 90%	54 / 304 (17.76 %)	0 / 502 (0.00 %)

(b) ML3 with five clusters

Table 29: Overlaps of detected clusters with known cell types from Ramani *et al.* (2017), ML3 batch. Approximate k-nn with MinHash, spectral clustering. More clusters can increase the accuracy of the detected cell types. Parameters: Spectral clustering. MinHash with 5000 hash functions, full-nearest neighbors graph, 13 principal components, inter and intra-chromosomal contacts, umap: n_neighbors 47, min_dist 0.33, n_components 2 for two clusters, n_components 9 for five clusters.

Cluster	K562 (301 cells)	GM12878 (501 cells)
Cluster 0 (384 cells)	248 cells / 64.6%	136 cells / 35.4%
Cluster 1 (418 cells)	53 cells / 12.6%	365 cells / 87.4%
Correct identified > 70%	0 / 301 (0 %)	365 / 501 (72.85 %)
Correct identified > 80%	0 / 301 (0 %)	365 / 501 (72.85 %)
Correct identified > 90%	0 / 301 (0 %)	0 / 501 (0.00 %)

(a) ML3 with two clusters

Cluster	K562 (301 cells)	GM12878 (501 cells)
Cluster 0 (168 cells)	0 cells / 0%	168 cells / 100%
Cluster 1 (134 cells)	16 cells / 11.9%	118 cells / 88.1%
Cluster 2 (201)	195 cells / 97%	6 cells / 3%
Cluster 3 (205)	0 cells / 0%	205 cells / 100%
Cluster 4 (94)	90 cells / 95.7%	4 cells / 4.3%
Correct identified > 70%	285 / 301 (94.68 %)	491 / 501 (98.00 %)
Correct identified > 80%	285 / 301 (94.68 %)	491 / 501 (98.00 %)
Correct identified > 90%	285 / 301 (94.68 %)	373 / 501 (74.45 %)

(b) ML3 with four clusters

Table 30: Overlaps of detected clusters with known cell types from Ramani *et al.* (2017), ML3 batch. Results computed with Zhou's scHiCluster. More clusters can increase the accuracy of the detected cell types. Four cells had to be removed because they contained chromosomes with no interactions. Zhou's scHiCluster cannot handle this and crashes.

8 Cluster results on Ramani 10 kb

Cluster	HeLa (269 cells)	HAP1 (254 cells)
Cluster 0 (278 cells)	121 cells / 43.53 %	157 cells / 56.47 %
Cluster 1 (245 cells)	148 cells / 60.41 %	97 cells / 39.59 %
Correct identified > 70%	0 / 269 (0.00 %)	0 / 254 (0.00 %)
Correct identified > 80%	0 / 269 (0.00 %)	0 / 254 (0.00 %)
Correct identified > 90%	0 / 269 (0.00 %)	0 / 254 (0.00 %)

Table 31: Ramani ML1 data with two clusters. 10 kb resolution.

Cluster	K562 (304 cells)	GM12878 (502 cells)
Cluster 0 (478 cells)	106 cells / 22.18 %	372 cells / 77.82 %
Cluster 1 (328 cells)	198 cells / 60.37 %	130 cells / 39.63 %
Correct identified > 70%	0 / 304 (0.00 %)	372 / 502 (74.10 %)
Correct identified > 80%	0 / 304 (0.00 %)	0 / 502 (0.00 %)
Correct identified > 90%	0 / 304 (0.00 %)	0 / 502 (0.00 %)

Table 32: Ramani ML3 data with two clusters, 10 kb resoltion.

9 Runtime and memory usage

The measurement of runtimes of algorithms with a high I/O and the requirement for a fast parallelization are very environment dependent. To give a broader overview, the here presented numbers are from a virtual machine with NFS storage and a state-of-the-art computer with a modern SSD. To show the impact of the number of hash functions, run times and memory usage are shown with a low number and a high number of hash function.

Method	Runtime	Memory
Raw and K-Means	39:15 min	7.2 GB
Raw and Spectral	01:29 min	4.5 GB
PCA and K-Means	05:37 min	170 GB
PCA and Spectral	05:35 min	170 GB
scikit-learn k-nn k = 2472 and k-means	01:19 min	4.5 GB
scikit-learn k-nn k = 2472 and Spectral	01:25 min	4.5 GB
scHicClusterMinHash k = 2472 and k-means	01:30 min	7.6 GB
scHicClusterMinHash k = 2472 and Spectral	01:35 min	7.6 GB
scHicClusterMinHash eucl. k = 2472 and k-means	02:04 min	7.6 GB
scHicClusterMinHash eucl. k = 2472 and Spectral	02:04 min	7.6 GB
Zhou's scHiCluster CPU	13:55 min	4.0 GB

Table 33: Runtimes and memory usage with 1 Mb 2472 cells on a single-cell Hi-C matrix. Data from Nagano *et al.* (2017) Diploid cells, with 12 clusters. For clustering k-means and spectral clustering are used, scHicClusterMinHash with 800 hash functions, k=2472, applied PCA and 100 principal components for clustering. All results computed on 2x Intel XEON E5-2630 v4 @ 2.20GHz 2x 10 cores / 2x 20 threads, 1 TB memory.

Method	Runtime	Memory
Raw and K-Means	12:22 min	7.2 GB
Raw and Spectral	1:37 min	4.0 GB
PCA and K-Means	-	> 128 GB
PCA and Spectral	-	> 128 GB
scikit-learn k-nn k = 2472 and K-means	1:24 min	4.0 GB
scikit-learn k-nn k = 2472 and Spectral	1:26 min	4.0 GB
MinHash k = 2472 and K-means	0:57 min	7.6 GB
MinHash k = 2472 and Spectral	0:59 min	7.6 GB
MinHash euclidean k = 2472 and K-means	1:55 min	7.6 GB
MinHash euclidean k = 2472 and Spectral	1:56 min	7.6 GB
Zhou's scHiCluster CPU	14:02 min	4.0 GB
Zhou's scHiCluster GPU	07:17 min	3.7 GB

Table 34: Runtimes and memory usage with 1 Mb on a single-cell Hi-C matrix with 2472 cells. Data from Nagano *et al.* (2017) Diploid cells, with 12 clusters. For clustering K-means and spectral clustering are used, MinHash with 800 hash functions and activated PCA. All results computed on AMD Ryzen 3700X 8 cores / 16 threads, 128 GB memory; Nvidia GTX 1070 8 GB memory.

9.1 High number of hash functions

Method	Runtime	Memory
MinHash h = 800	0:42 min	1.6 GB
MinHash h = 2000	0:47 min	1.6 GB
MinHash h = 8000	1:10 min	1.7 GB
MinHash h = 15000	1:36 min	1.9 GB
MinHash h = 20000	2:00 min	2 GB
Zhou's scHiCluster CPU	6:50 min	2.4 GB
Zhou's scHiCluster GPU	3:40 min	2.7 GB

Table 35: Runtimes and memory usage with 1 Mb resolution on a single-cell Hi-C matrix with 1275 cells. Normalized to a read coverage of 100,000 reads and interaction values smaller 1 are *kept.* Data from Nagano *et al.* (2017) Diploid cells, with 12 clusters. For clustering spectral clustering is used, MinHash with a different number of hash functions *h*. All results computed on AMD Ryzen 3700X 8 cores / 16 threads, 128 GB memory; Nvidia GTX 1070 8 GB memory.

Method	Runtime	Memory
MinHash h = 800	04:04 min	13.9 GB
MinHash h = 4000	05:42 min	14.0 GB
MinHash h = 8000	07:33 min	14.1 GB
MinHash h = 15000	11:05 min	14.3 GB
MinHash h = 20000	13:30 min	14.5 GB
MinHash h = 40000	23:27 min	15.2 GB

Table 36: Runtimes and memory usage with 10 kb resolution on a single-cell Hi-C matrix with 1088 cells. Normalized to a read coverage of 100,000 reads and interaction values smaller 1 are *kept.* Data from Nagano *et al.* (2017) Diploid cells, with 14 clusters. For clustering spectral clustering is used, MinHash with a different number of hash functions *h*. All results computed on AMD Ryzen 3700X 8 cores / 16 threads, 128 GB memory; Nvidia GTX 1070 8 GB memory.

10 Cluster profiles Nagano data

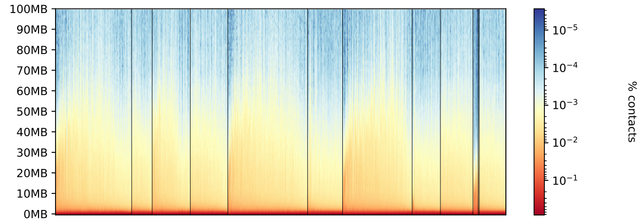

(a) Raw Matrices

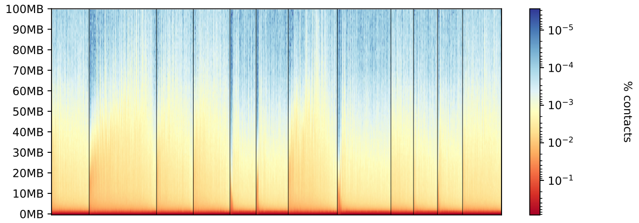

(b) MinHash Euclidean

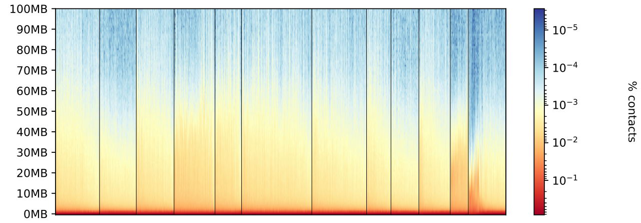

(c) MinHash k = 100

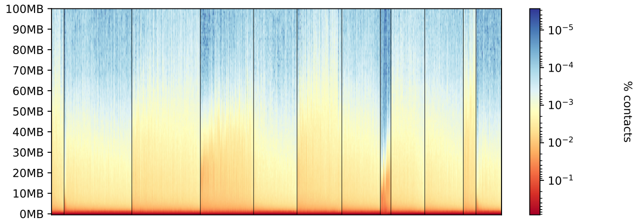

(d) MinHash no PCA

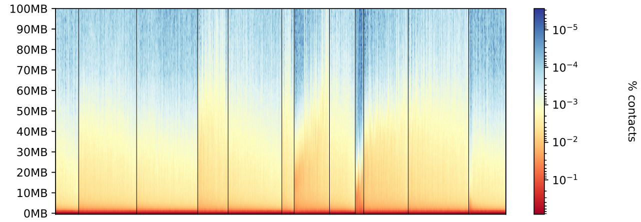

(e) MinHash k = 1275, no UMAP

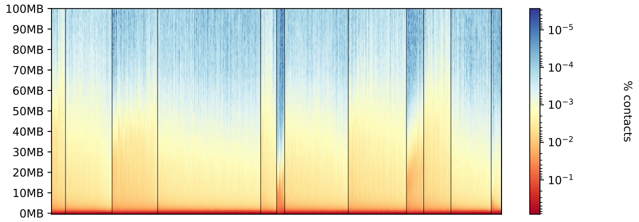

(f) MinHash k = 1275, no PCA, no UMAP

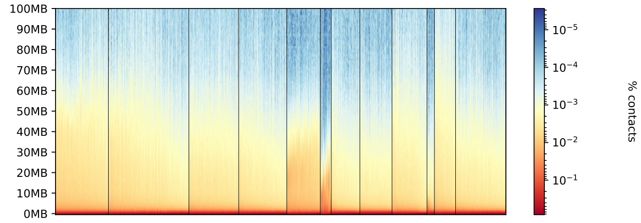

(g) Scikit-learn k-nn

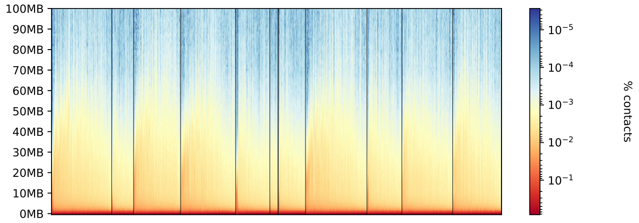

(h) Scikit-learn k-nn k = 1275 k-means, 100 PC

Figure S 14: Cluster profile of the different clusters on 1275 cells from Nagano *et al.* (2017) Diploid cells. K-Means clustering was applied on all datasets, MinHash with intra-chromosomal data, PCA, UMAP and a full k-nn if not defined otherwise. Clustering on raw single-cell Hi-C interaction matrix (S 14a, S 14b k-nn with MinHash and the additional euclidean distance computation; S 14c MinHash with 100 nearest neighbors; S 14d MinHash without an intermediate PCA on the k-nn, S 14e MinHash with a PCA but no UMAP, S 14f MinHash only, without PCA and UMAP. S 14g shows the results if inter- and intrachromosomal data are used to create the k-nn with MinHash; S 14h shows the result of k-means applied on a k-nn with k=1275 using Scikit-learns' k-nn implementation.

11 Consensus matrices Nagano data

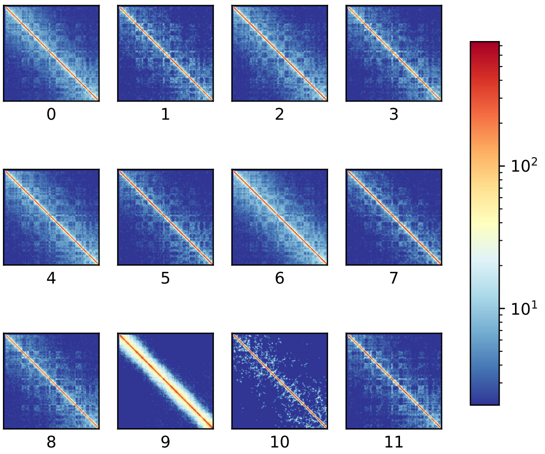

(a) Raw K-means

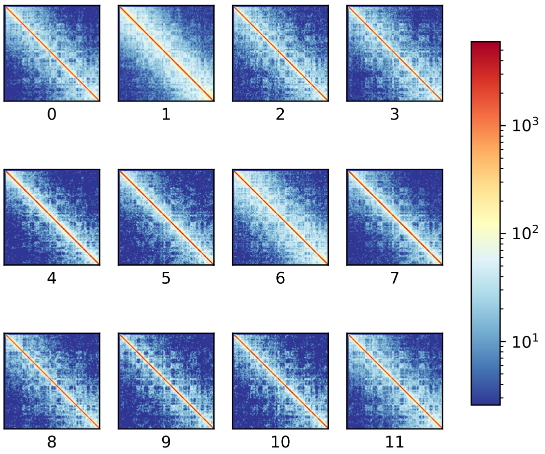

(b) MinHash euclidean

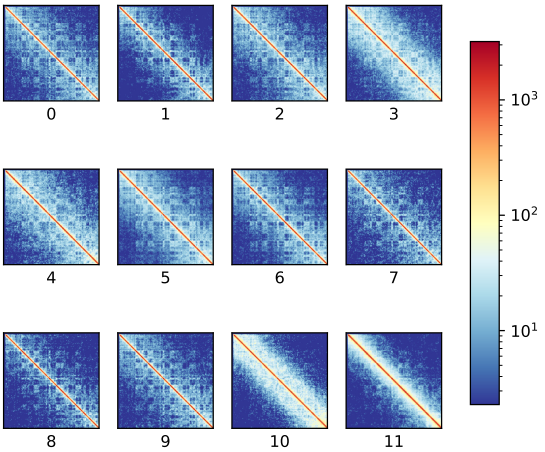

(c) MinHash k = 100

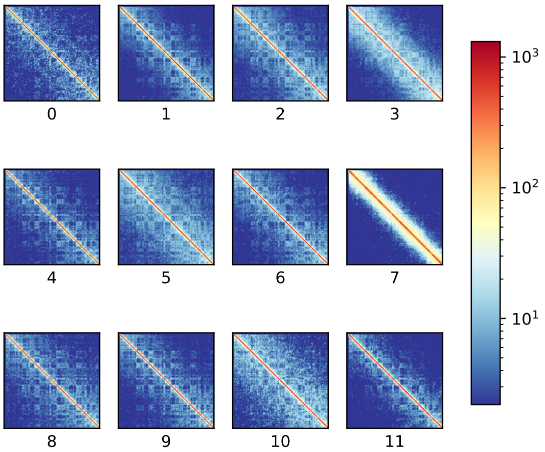

(d) MinHash, no PCA

Figure S 15: Consensus matrices of the different clusters on 1275 cells from Nagano *et al.* (2017) Diploid cells, chromosome 6. K-Means clustering was applied on all datasets, MinHash with intra-chromosomal data, PCA, UMAP and a full k-nn if not defined otherwise. Clustering on raw single-cell Hi-C interaction matrix (S 15a, S 15b k-nn with MinHash and the additional euclidean distance computation; S 15c MinHash with 100 nearest neighbors; S 15d MinHash without an intermediate PCA on the k-nn

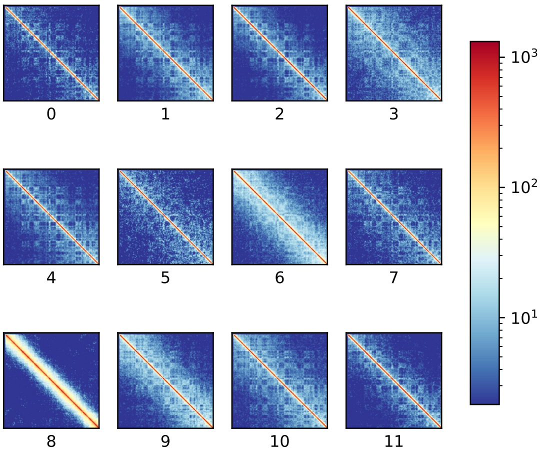

(a) MinHash, no UMAP

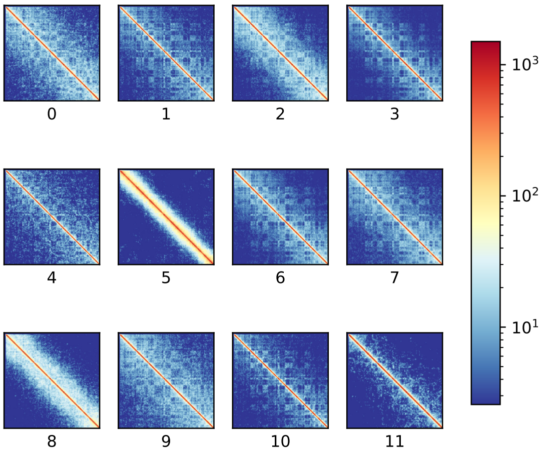

(b) MinHash. no PCA, no UMAP

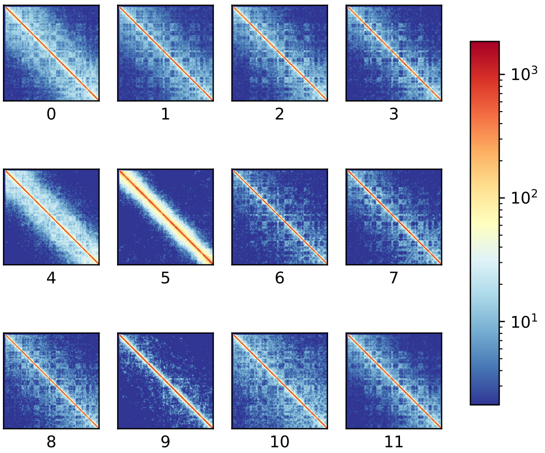

(c) inter- and intra-chromosomal contacts

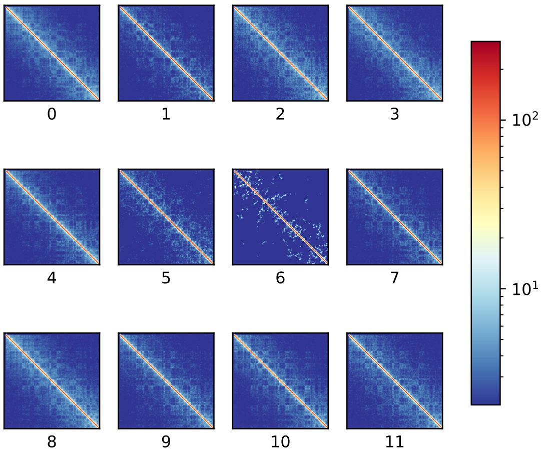

(d) Scikit-learn k-nn

Figure S 16: Consensus matrices of the different clusters on 1275 cells from Nagano *et al.* (2017) Diploid cells, chromosome 6. K-Means clustering was applied on all datasets, MinHash with intra-chromosomal data, PCA, UMAP and a full k-nn if not defined otherwise. S 16a MinHash with a PCA but no UMAP, S 16b MinHash only, without PCA and UMAP. S 16c shows the results if inter- and intrachromosomal data are used to create the k-nn with MinHash; S 16d shows the result of k-means applied on a k-nn with k=1275 using scikit-learns k-nn implementation.

12 Scatter plots cell labels

12.1 Nagano 1 Mb

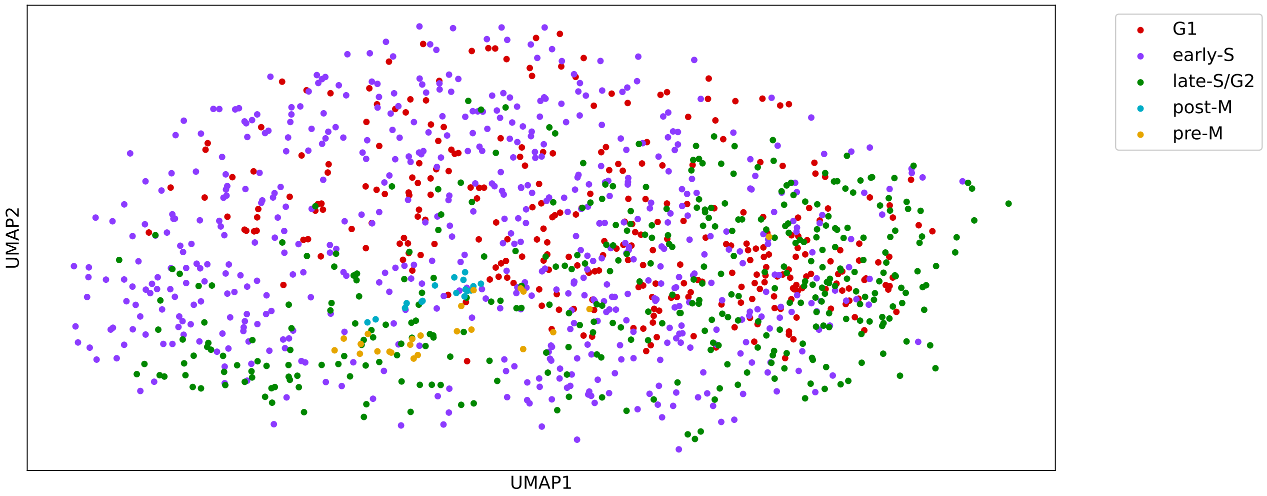

(a) k-nn MinHash on Nagano 1MB cell coloring UMAP dimensions 5

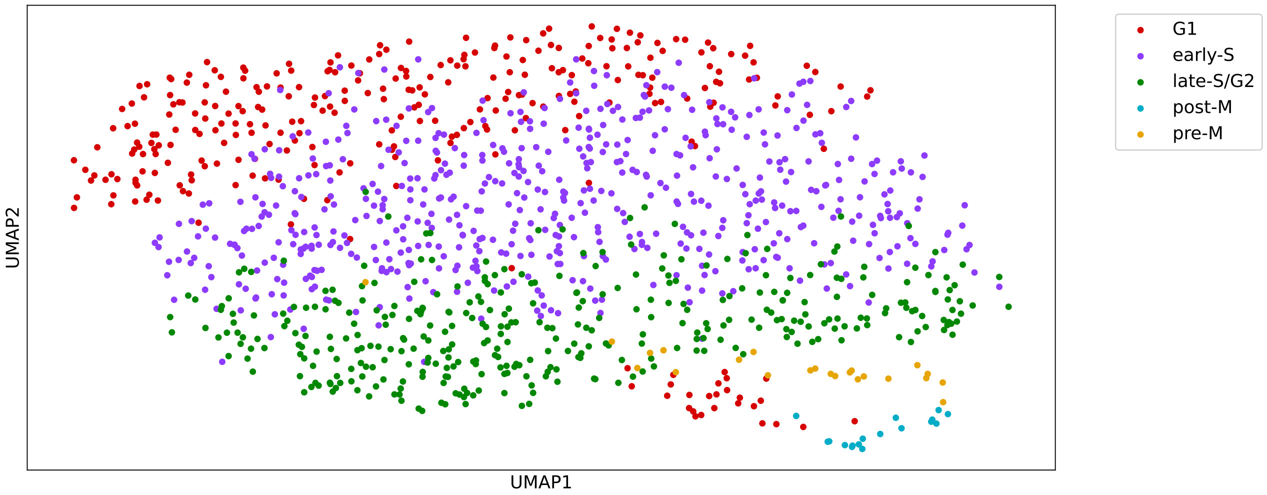

(b) k-nn MinHash on Nagano 1MB cell coloring UMAP dimensions 2

Figure S 17

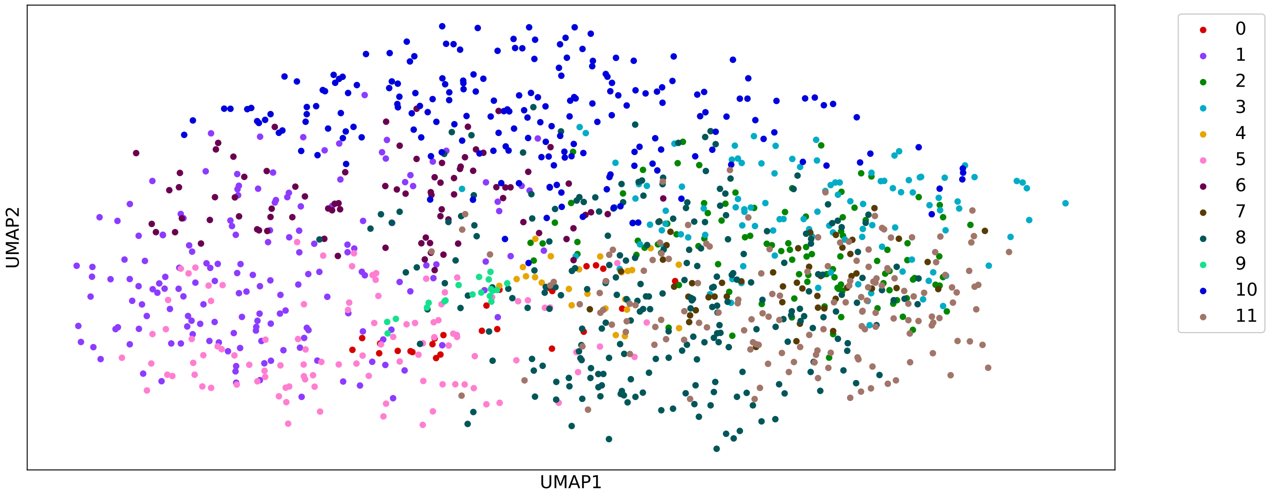

(c) k-nn MinHash on Nagano 1MB cell cluster result UMAP dimensions 5

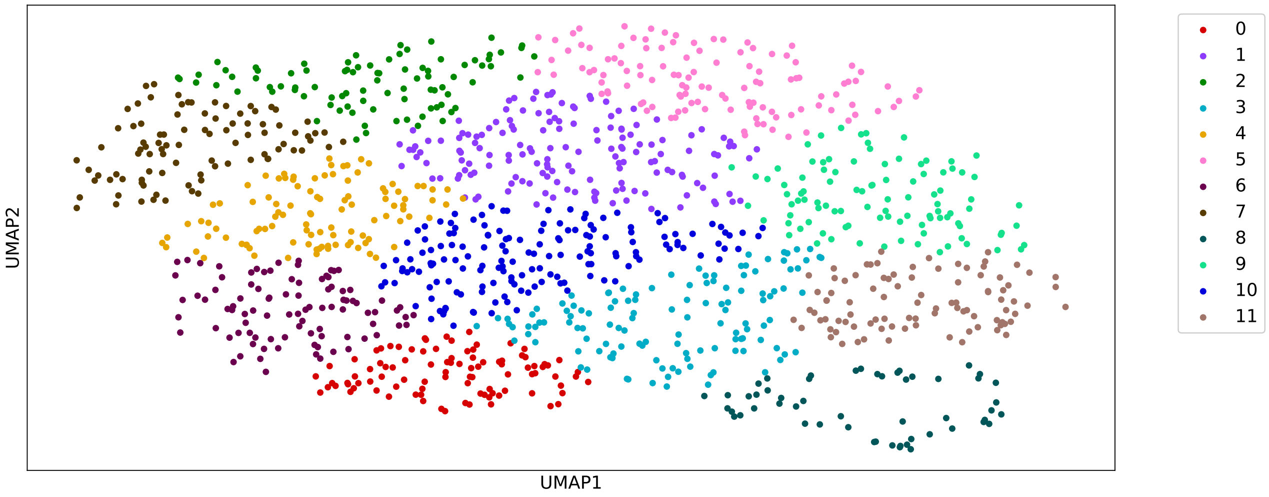

(d) k-nn MinHash on Nagano 1MB cell cluster result UMAP dimensions 2

Figure S 17

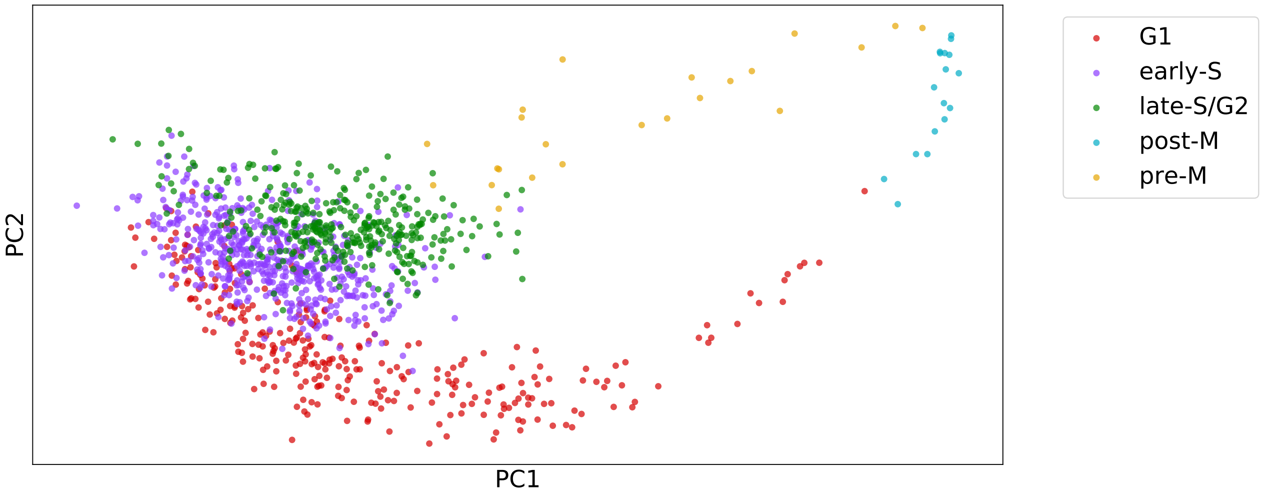

(a) Zhou's scHiCluster Nagano 1MB cell coloring

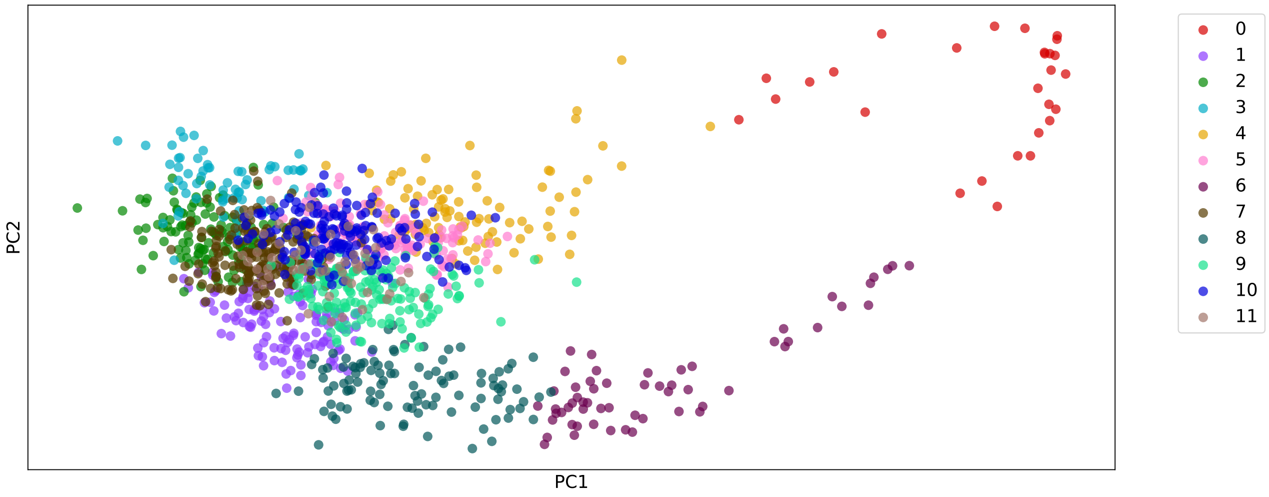

(b) Zhou's scHiCluster Nagano 1MB cell cluster result

Figure S 18

12.2 Ramani 1 Mb

12.2.1 approximate k-nn with MinHash

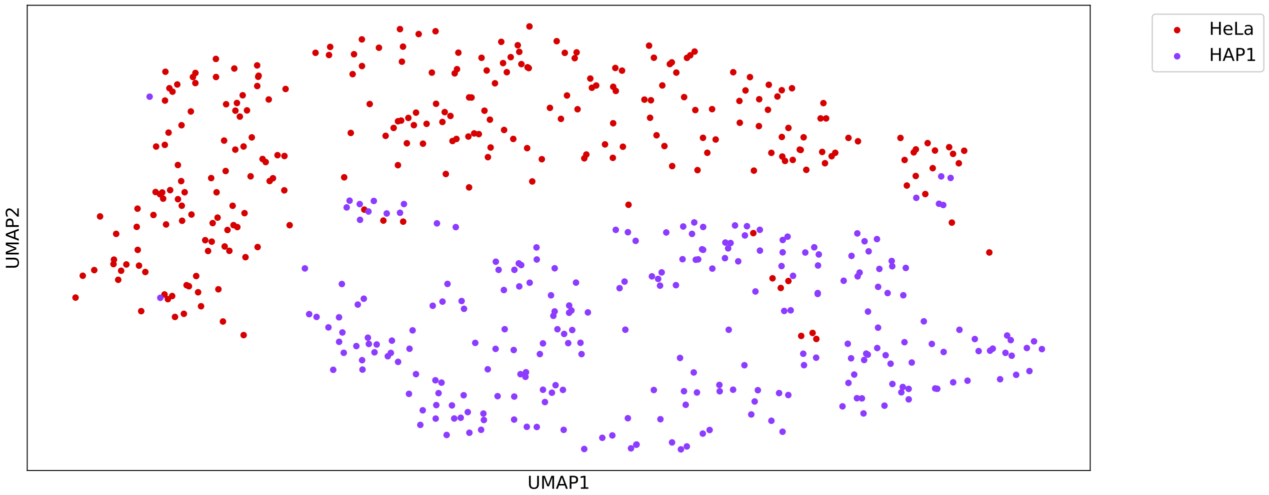

(a) ML1 cell types

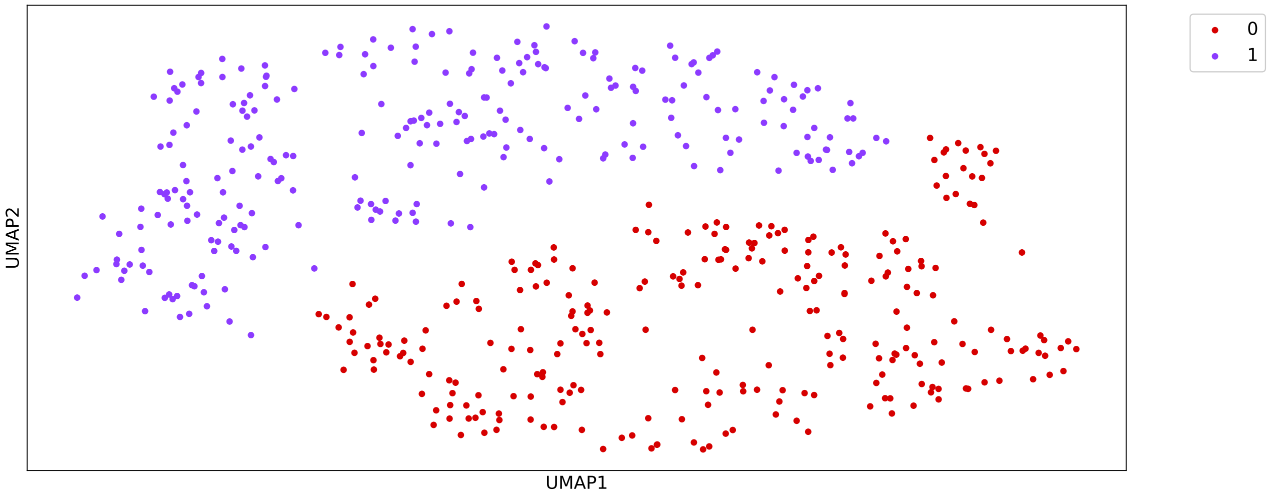

(b) ML1 detected cluster c = 2

Figure S 19: Embedding of Ramani cell type data. 1 MB resolution, ML1 with approximate k-nn based on MinHash, 8 principal components, UMAP embedding and spectral clustering.

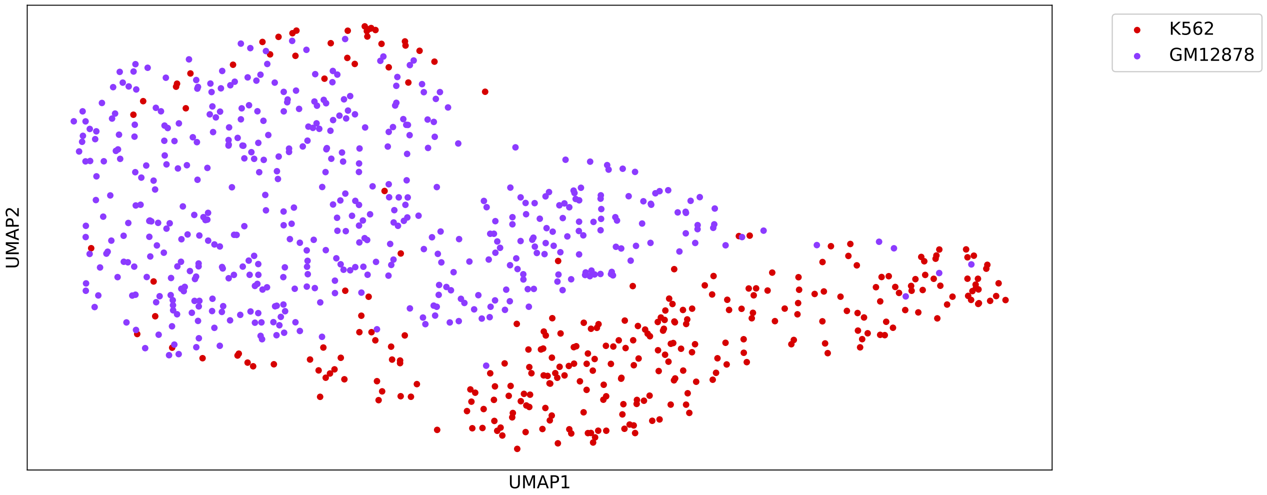

(a) ML3 cell types

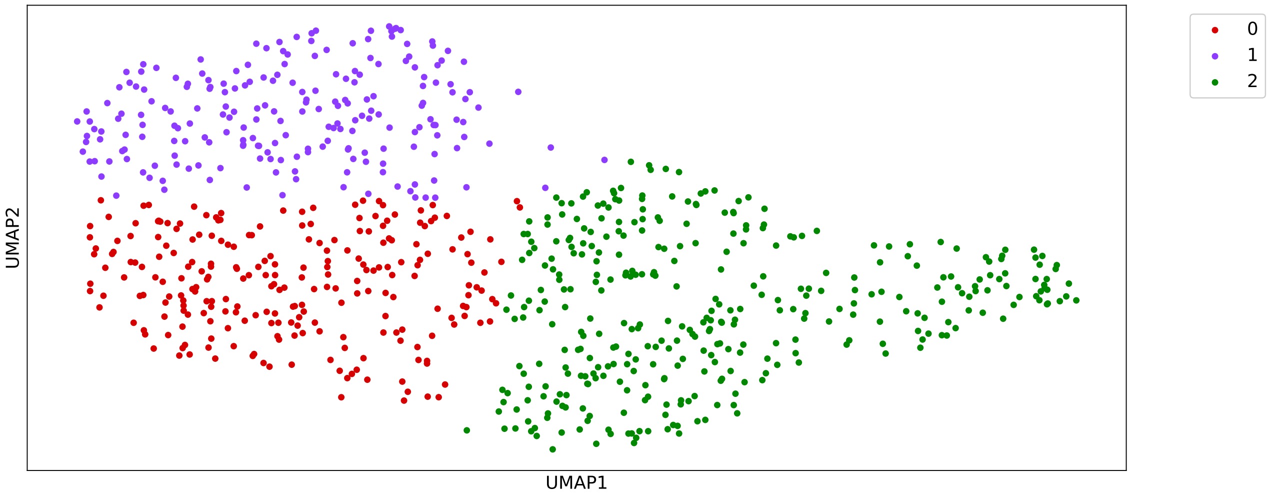

(b) ML3 detected cluster c = 3

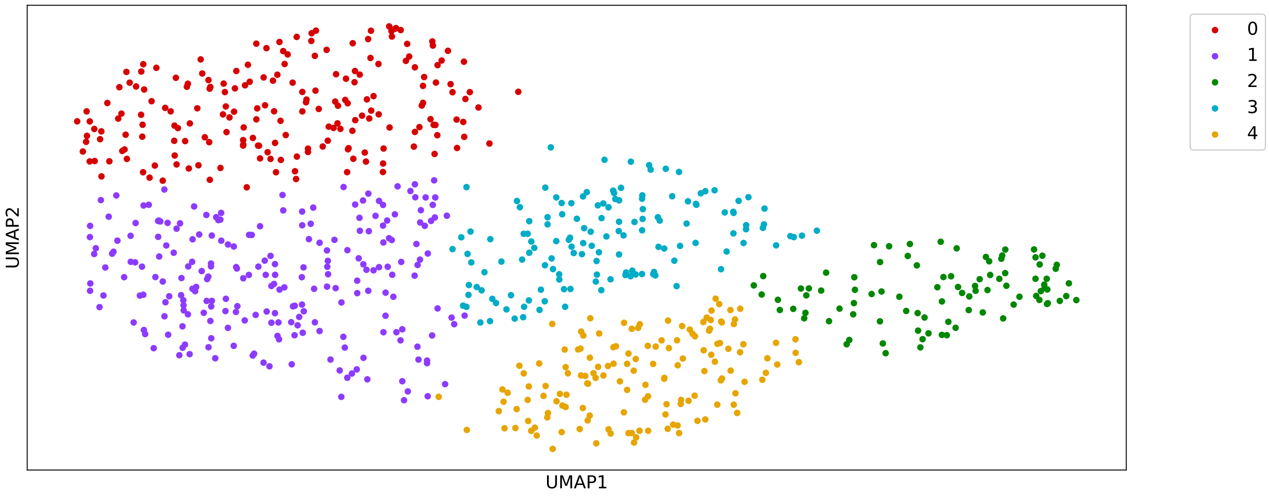

(c) ML3 detected cluster c = 5

Figure S 20: Embedding of Ramani cell type data. 1 MB resolution, ML3, with approximate k-nn based on MinHash, 8 principal components, UMAP embedding and spectral clustering. To detect more clusters than cell types can be beneficial.

12.2.2 Zhou's scHiCluster

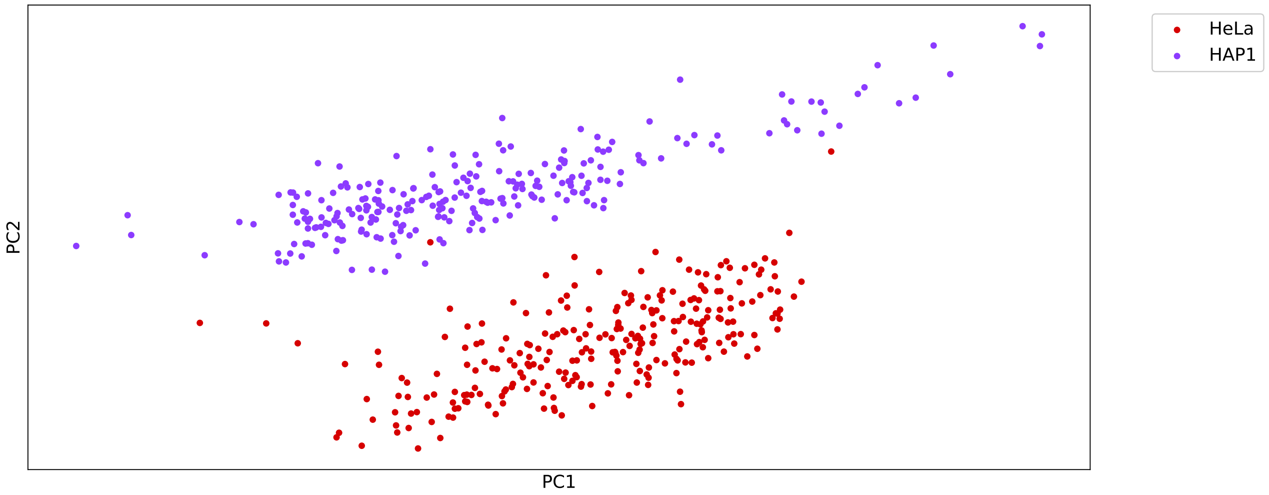

(a) ML1 cell types

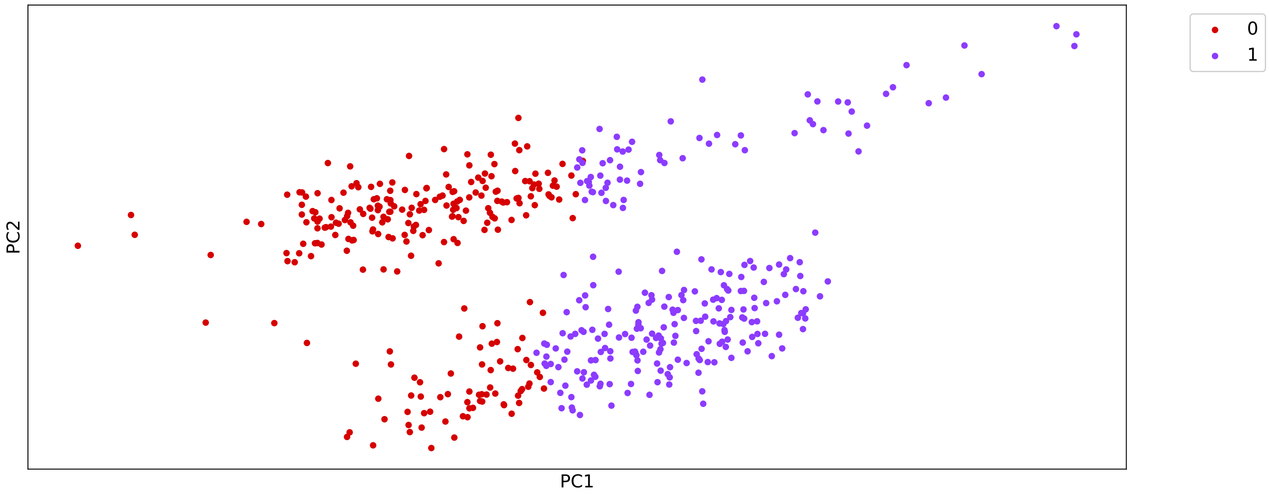

(b) ML1 detected cluster c = 2

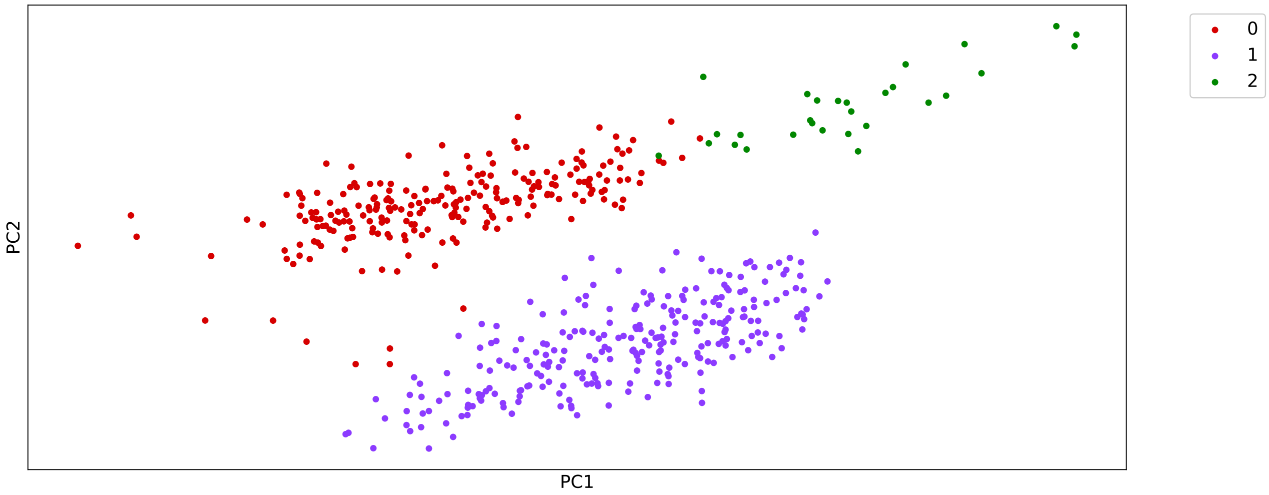

(c) ML1 detected cluster c = 3

Figure S 21: Embedding of Ramani cell type data. 1 MB resolution, ML1 with Zhou's scHiCluster. To detect more clusters than cell types can be beneficial.

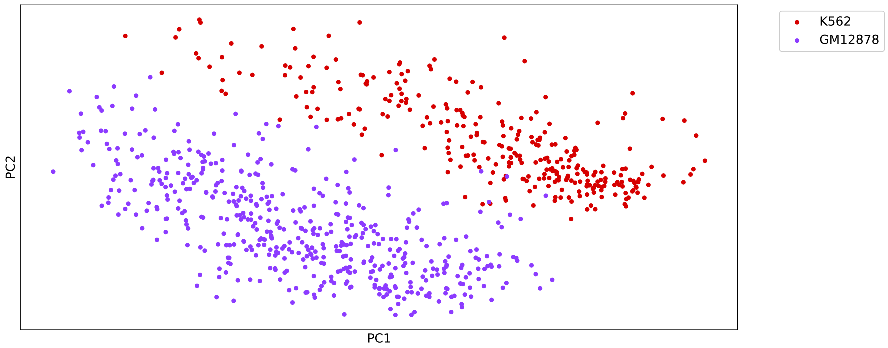

(a) ML3 cell types

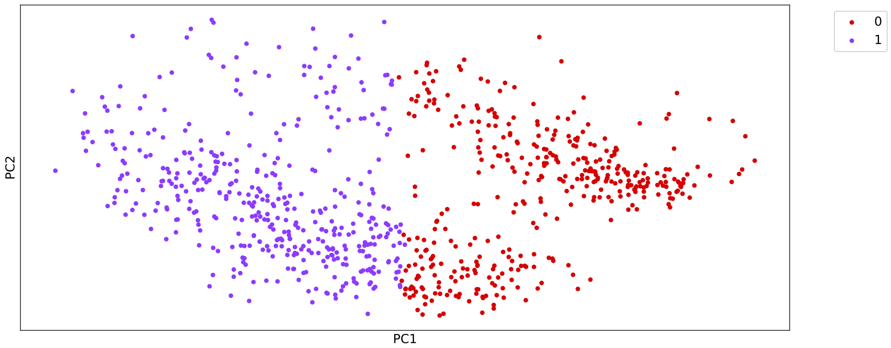

(b) ML3 detected cluster c = 2

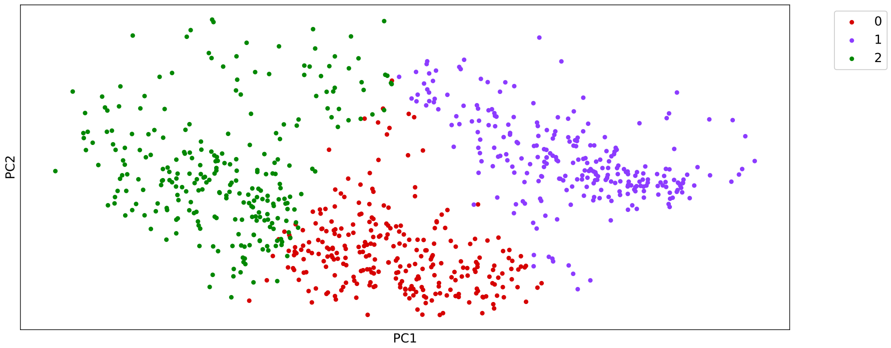

(c) ML3 detected cluster c = 3

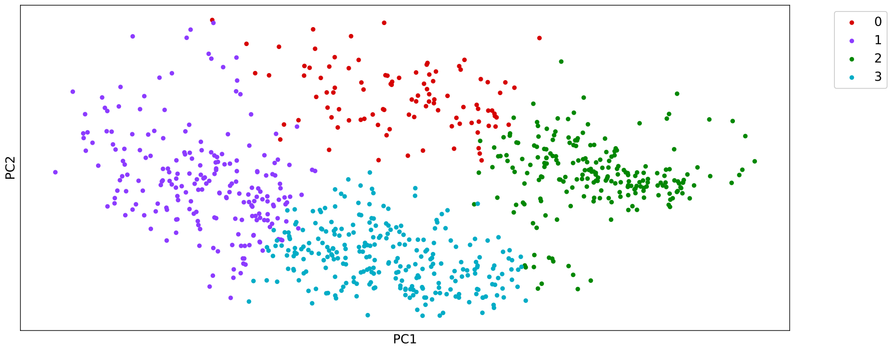

(d) ML3 detected cluster c = 4

Figure S 22: Embedding of Ramani cell type data. 1 MB resolution, ML3, with Zhou's scHiCluster. To detect more clusters than cell types can be beneficial.

13 Data collection and pre-processing

All used data is from Nagano *et al.* (2017): GEO94489; Gassler *et al.* (2017): GSE100569 and Ramani *et al.* (2017): GSE84920; and was pre-processed with scHiCExplorer (Wolff *et al.* (2020a)) version 7[*]. The raw data was quality controlled and read coverage normalized, it is available on Zenodo[†]. The single-cell Hi-C interaction matrices are stored in the *scool*[‡] file format (Wolff *et al.* (2020b)), available in the cooler (Abdennur and Mirny (2019)) package since version 0.8.9.

[*] https://github.com/joachimwolff/scHiCExplorer/tree/7
[†] https://doi.org/10.5281/zenodo.4308298
[‡] https://cooler.readthedocs.io/en/latest/schema.html#single-cell-single-resolution

References

Abdennur, N. and Mirny, L. A. (2019). Cooler: scalable storage for Hi-C data and other genomically labeled arrays. *Bioinformatics*, **36**(1), 311–316.

Gassler, J. *et al.* (2017). A mechanism of cohesin-dependent loop extrusion organizes zygotic genome architecture. *The EMBO journal*, **36**(24), 3600–3618.

Nagano, T. *et al.* (2017). Cell-cycle dynamics of chromosomal organization at single-cell resolution. *Nature*, **547**(7661), 61.

Ramani, V. *et al.* (2017). Massively multiplex single-cell hi-c. *Nature methods*, **14**(3), 263–266.

Wolff, J. *et al.* (2020a). Galaxy HiCExplorer 3: a web server for reproducible Hi-C, capture Hi-C and single-cell Hi-C data analysis, quality control and visualization. *Nucleic Acids Research*. gkaa220.

Wolff, J. *et al.* (2020b). Scool: a new data storage format for single-cell Hi-C data. *Bioinformatics*. btaa924.
